# Immune correlates of protection by mRNA-1273 vaccine against SARS-CoV-2 in nonhuman primates

**DOI:** 10.1126/science.abj0299

**Published:** 2021-09-17

**Authors:** Kizzmekia S. Corbett, Martha C. Nason, Britta Flach, Matthew Gagne, Sarah O’Connell, Timothy S. Johnston, Shruti N. Shah, Venkata Viswanadh Edara, Katharine Floyd, Lilin Lai, Charlene McDanal, Joseph R. Francica, Barbara Flynn, Kai Wu, Angela Choi, Matthew Koch, Olubukola M. Abiona, Anne P. Werner, Juan I. Moliva, Shayne F. Andrew, Mitzi M. Donaldson, Jonathan Fintzi, Dillon R. Flebbe, Evan Lamb, Amy T. Noe, Saule T. Nurmukhambetova, Samantha J. Provost, Anthony Cook, Alan Dodson, Andrew Faudree, Jack Greenhouse, Swagata Kar, Laurent Pessaint, Maciel Porto, Katelyn Steingrebe, Daniel Valentin, Serge Zouantcha, Kevin W. Bock, Mahnaz Minai, Bianca M. Nagata, Renee van de Wetering, Seyhan Boyoglu-Barnum, Kwanyee Leung, Wei Shi, Eun Sung Yang, Yi Zhang, John-Paul M. Todd, Lingshu Wang, Gabriela S. Alvarado, Hanne Andersen, Kathryn E. Foulds, Darin K. Edwards, John R. Mascola, Ian N. Moore, Mark G. Lewis, Andrea Carfi, David Montefiori, Mehul S. Suthar, Adrian McDermott, Mario Roederer, Nancy J. Sullivan, Daniel C. Douek, Barney S. Graham, Robert A. Seder

**Affiliations:** 1 Vaccine Research Center, National Institute of Allergy and Infectious Diseases, National Institutes of Health, Bethesda, MD 20892, USA.; 2 Biostatistics Research Branch, Division of Clinical Research, National Institute of Allergy and Infectious Diseases, National Institutes of Health, Bethesda, MD 20892, USA.; 3 Center for Childhood Infections and Vaccines of Children’s Healthcare of Atlanta, Department of Pediatrics, Department of Microbiology and Immunology, Emory Vaccine Center, Emory University, Atlanta, GA 30322, USA.; 4 Department of Surgery, Duke University Medical Center, Durham, NC 27708, USA.; 5 Moderna Inc., Cambridge, MA 02139, USA.; 6 Bioqual Inc., Rockville, MD 20850, USA.; 7 Infectious Disease Pathogenesis Section, National Institute of Allergy and Infectious Diseases, National Institutes of Health, Bethesda, MD 20892, USA.; 8 Department of Microbiology and Immunology, Atlanta, GA 30329, USA.

## Abstract

Immune correlates of protection can be used as surrogate endpoints for vaccine efficacy. Here, nonhuman primates (NHPs) received either no vaccine or doses ranging from 0.3 to 100 μg of the mRNA-1273 severe acute respiratory syndrome coronavirus 2 (SARS-CoV-2) vaccine. mRNA-1273 vaccination elicited circulating and mucosal antibody responses in a dose-dependent manner. Viral replication was significantly reduced in bronchoalveolar lavages and nasal swabs after SARS-CoV-2 challenge in vaccinated animals and most strongly correlated with levels of anti–S antibody and neutralizing activity. Lower antibody levels were needed for reduction of viral replication in the lower airway than in the upper airway. Passive transfer of mRNA-1273–induced immunoglobulin G to naïve hamsters was sufficient to mediate protection. Thus, mRNA-1273 vaccine–induced humoral immune responses are a mechanistic correlate of protection against SARS-CoV-2 in NHPs.

## Antibodies dovetail with protection

Immune correlates of protection are clinical end points used to gauge vaccine-induced immunogenicity and protection. Corbett *et al*. studied nonhuman primate (NHP) immune responses to various doses of the mRNA-1273 (Moderna) vaccine to provide a range of immune responses and protective outcomes. They determined that circulating spike protein–specific antibodies correlated with protection against severe acute respiratory syndrome coronavirus 2 (SARS-CoV-2) replication in the airways. Passively transferred NHP antibodies were sufficient to mediate protection against SARS-CoV-2 challenge in hamsters, emphasizing that antibodies are mechanistic correlates. Protection of the lower respiratory tract required lower serum antibody concentrations, possibly explaining why most current vaccines are highly effective against severe lower airway disease. The higher antibody threshold required for reducing upper airway infection has potential implications for boosting to limit transmission. —STS

## Abstract (structured)

**INTRODUCTION:**

Mass vaccination against severe acute respiratory syndrome coronavirus 2 (SARS-CoV-2) offers the most efficient public health intervention to control the COVID-19 pandemic. Two mRNA-based vaccines, Moderna’s mRNA-1273 and Pfizer/BioNTech’s BNT162b2, both of which encode the prefusion-stabilized spike glycoprotein S-2P, showed >94% efficacy against symptomatic COVID-19 in interim phase 3 analyses and are currently being administered globally. Several other vaccines have shown 60 to 80% efficacy against symptomatic COVID-19 in phase 3 trials, and a number of candidate vaccines are in earlier stages of clinical development. An immune correlate of protection can be used to inform potential dose reduction, advance approval of other vaccine candidates in lieu of phase 3 efficacy data, extend indications for use to other age groups, and provide insights into the immune mechanisms of protection.

**RATIONALE:**

In assessing immunogenicity and protection of vaccines against SARS-CoV-2, nonhuman primates (NHPs) have been a useful model for clinical translation. NHP innate immune responses and B and T cell repertoires show much greater similarity to humans than do those of rodents, allowing immune responses to be assessed using clinically applicable vaccine doses and regimens. After SARS-CoV-2 infection, NHPs have transient viral replication in the upper and lower airways and mild inflammation in the lung that recapitulates the features of mild infection in humans. Here, we used immunogenicity and protection assessments from a previous NHP mRNA-1273 vaccine study combined with new data from a dose de-escalation study. We evaluated how multiple measurements of humoral and cellular immunity correlate with the reduction of viral replication in the upper and lower airway after challenge. Antibodies in bronchoalveolar lavage (BAL) and nasal swabs (NS) after vaccination were analyzed to assess site-specific immune correlates. Finally, immunoglobulin G (IgG) from mRNA-immunized NHPs was passively transferred in a highly pathogenic Syrian hamster SARS-CoV-2 challenge model to determine whether these antibodies were sufficient for protection.

**RESULTS:**

NHPs received either no vaccine or doses ranging from 0.3 to 100 μg of mRNA-1273, an mRNA vaccine encoding S-2P, at weeks 0 and 4. mRNA-1273 vaccination elicited circulating and mucosal antibody responses in a dose-dependent manner. Using the World Health Organization standard for measuring S-specific IgG, a 10-fold increase in S-binding titers was associated with ~10-fold reductions in viral replication in BAL and NS after challenge. No animal with S-specific IgG >336 IU/ml had BAL subgenomic RNA (sgRNA) >10,000 copies/ml, and no animal with S-specific IgG >645 IU/ml had NS sgRNA >100,000 copies/swab, so these were chosen as the thresholds for protection. These reductions in viral replication in BAL were associated with limited inflammation and viral antigen detection in lung tissue. Finally, passive transfer of vaccine-induced IgG from NHPs to naïve hamsters was sufficient to mediate protection.

**CONCLUSION:**

mRNA-1273 vaccine–induced antibody responses are a mechanistic correlate of protection against SARS-CoV-2 infection in NHPs. Protection in the lower respiratory tract was achieved at lower serum antibody concentrations than in the upper respiratory tract. These data explain in part the consistent finding that vaccine efficacy against severe lower tract disease is greater than that against mild upper tract disease. These findings have potential implications for how additional boosting may sustain protection against severe disease in the lower respiratory tract and limit mild infection and transmission by enhancing the immunity required in the upper respiratory tract.

## Teaser

mRNA-1273 vaccine–induced antibody responses are a mechanistic correlate of protection against SARS-CoV-2 infection in nonhuman primates.

## Graphical abstract

**Figure:**
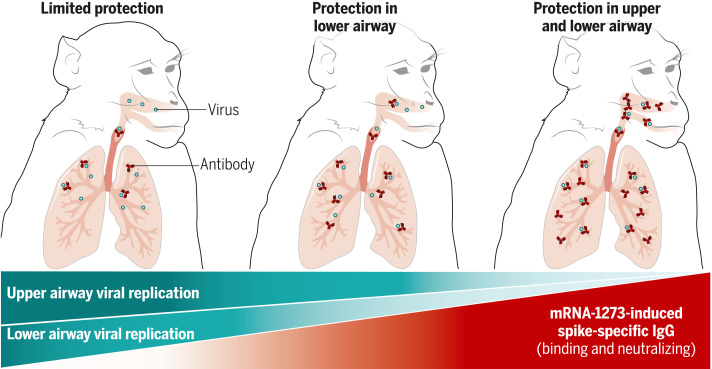
mRNA-1273 vaccine–induced humoral immune responses are a mechanistic correlate of protection against SARS-CoV-2. Levels of serum and mucosal spike-specific IgG in mRNA-1273–vaccinated NHPs were inversely correlated with the reduction of viral replication in the upper airway and lower airway after SARS-CoV-2 challenge.

Severe acute respiratory syndrome coronavirus 2 (SARS-CoV-2), the causative agent of COVID-19, has resulted in >180 million infections and 4 million deaths worldwide as of 13 July 2021 (*1*). Mass vaccination offers the most efficient public health intervention to control the pandemic. Two mRNA-based vaccines, Moderna’s mRNA-1273 and Pfizer/BioNTech’s BNT162b2, both produce a stabilized version of the spike glycoprotein (*2*, *3*), show >94% efficacy against symptomatic COVID-19 in interim phase 3 analyses (*4*, *5*), and are currently being administered globally. Several other vaccines have shown 60 to 80% efficacy against COVID-19 in phase 3 trials (*6*, *7*), and several candidate vaccines are in earlier stages of clinical development (*8*). A critical issue for optimizing the use of COVID-19 vaccines is defining an immune correlate of protection. This predictor of vaccine efficacy can be used to inform potential dose reduction, advance approval of other vaccine candidates in lieu of phase 3 efficacy data, extend indications for use to other age groups, and provide insights into durability of protection, necessity for booster vaccination, and immune mechanisms of protection (*9*).

The nonhuman primate (NHP) model has been used to demonstrate immunogenicity and protective efficacy against SARS-CoV-2 with several vaccine candidates (*10*–*13*). The high level of protection achieved with mRNA vaccines in NHPs using clinically relevant dose regimens parallels results from human trials. This model exhibits upper and lower airway infection and pathology similar to clinical presentations of mild COVID-19 in humans (*14*). Although immune responses associated with protection after primary infection have been assessed in NHPs (*15*), there are no studies to date that have specifically defined immune correlates of protection in upper and lower airways after vaccination with any COVID-19 vaccine approved for use in humans.

We used immunogenicity and protection assessments from our previous NHP mRNA-1273 vaccine study (*13*) to test the hypothesis that serum antibody serves as an immune correlate of protection. Here, in a dose de-escalation study, we evaluated how multiple measurements of humoral and cellular immunity correlate with the reduction of viral replication in the upper and lower airway after challenge. Antibody analyses were also performed on bronchoalveolar lavages (BALs) and nasal washes after vaccination to assess correlates relevant for clinical disease and transmission, respectively. Finally, we demonstrated the ability of passively transferred immunoglobulin G (IgG) from mRNA-immunized NHPs to protect against SARS-CoV-2 infection of animals. Thus, this work delineates spike (S)–specific antibodies as a correlate of protection, highlights the ability of localized mucosal antibodies to control upper and lower airway viral replication, and confirms that mRNA-1273–induced IgG is sufficient for protection against SARS-CoV-2 infection in nonclinical models.

## Results

### mRNA-1273 vaccination elicits antibody responses in a dose-dependent manner

We previously demonstrated dose dependency of serum antibody responses in NHPs after vaccination with 10 or 100 μg of mRNA-1273, with high-level protection against SARS-CoV-2 challenge in both dose groups (fig. S1A) (*13*). These and other immunogenicity outcomes from an additional NHP study in which animals were vaccinated with 30 μg of mRNA-1273 (fig. S1B) were used to design a study to evaluate immune correlates of protection after mRNA-1273 vaccination in the current study (fig. S1C). Doses of mRNA-1273 ranging from 0.3 to 30 μg were administered in the standard clinical regimen at weeks 0 and 4 to generate a range of immune responses and protective outcomes.

We first assessed temporal serum S–specific antibody binding, avidity, and neutralization responses after prime and after boost. Consistent with our previous report (*13*), S-specific binding antibody (*2*, *3*) was increased over baseline after each immunization, reaching 7900 and 64,000 median reciprocal end-point titers by 4 weeks after prime and after boost, respectively, after immunization with 30 μg of mRNA-1273 (fig. S2A). There was an 8- to 10-fold increase in S-specific binding antibodies after the boost in all dose groups except for the 0.3-μg dose, for which boosting elicited 300-fold more S-specific antibodies. The boost improved not only antibody quantity but also binding strength, as shown by S-specific antibody avidity, which increased twofold after the boost in all vaccine groups except for the 0.3-μg dose, with no differences between the vaccine groups (fig. S2B). We detected neutralizing antibody responses against D614G, a previously dominant variant, in the 10- and 30-mg dose groups as early as 4 weeks after prime [30 mg; median reciprocal dose (ID_50_) = 76]. These responses are also found against many current circulating variants worldwide. The boost elicited neutralizing antibodies in all but the lowest (0.3-μg) dose groups and increased by ~1 log_10_ after boost in the highest-dose group (fig. S2C).

**Fig. 1.**
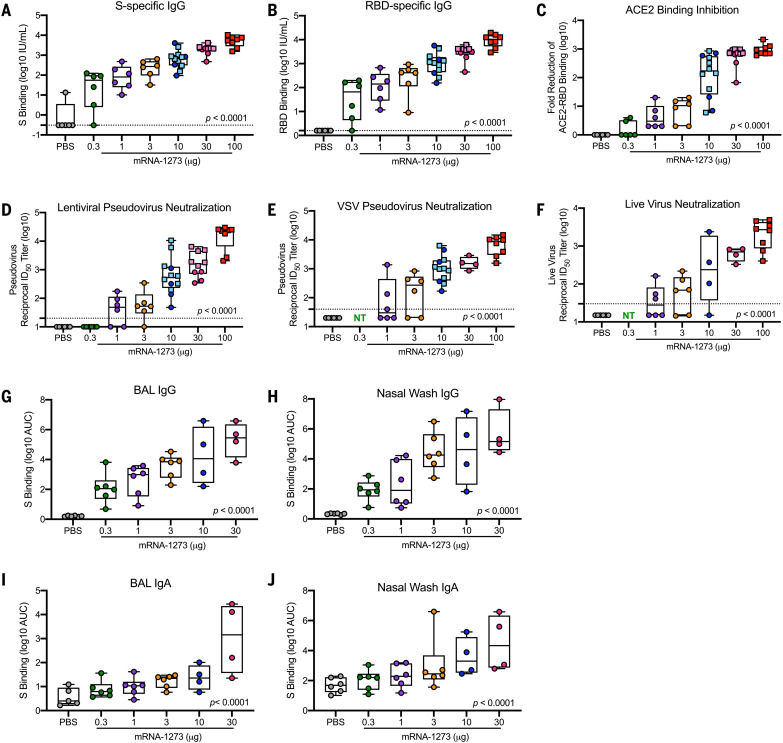
Antibody responses after mRNA-1273 immunization. (**A** to **F**) Rhesus macaques were immunized according to fig. S1 with PBS (gray) or mRNA-1273 (0.3 μg, green; 1 μg, purple; 3 μg, orange; 10 μg, blue; 30 μg, pink; 100 μg, red). Sera collected 4 weeks after boost, immediately before challenge, were assessed for SARS-CoV-2 S-specific (A) and RBD-specific (B) IgG by multi-array ELISA, inhibition of ACE2 binding to RBD (C), SARS-CoV-2 lentiviral-based pseudovirus neutralization (D), SARS-CoV-2 VSV-based pseudovirus neutralization (E), and SARS-CoV-2 EHC-83E focus reduction neutralization (F). (**G** to **J**) BAL [(G) and (I)] and nasal washes [(H) and (J)] collected 2 weeks after boost were assessed for SARS-CoV-2 S-specific IgG [(G) to (H)] and IgA [(I) to (J)] by multi-array ELISA. Squares represent NHPs in previous experiments (S1A, VRC-20-857.1; S1B, VRC-20-857.2) and circles represent individual NHPs in experiment S1C, VRC-20-857.4. Boxes and horizontal bars denote the interquartile range (IQR) and medians, respectively. Whisker end points are equal to the maximum and minimum values. Dotted lines indicate assay limits of detection, where applicable. NT, not tested. All measures were significantly correlated with dose (*P* < 0.0001), as determined by Spearman’s correlation test.

**Fig. 2.**
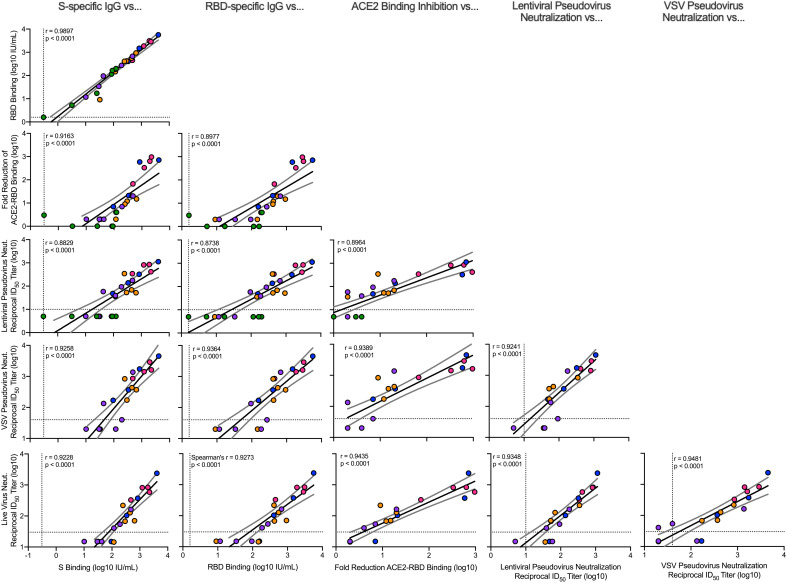
Correlations of humoral antibody analyses. Rhesus macaques were immunized according to fig. S1C. Plots show correlations between SARS-CoV-2 S-specific IgG, RBD-specific IgG, ACE2-binding inhibition, lentiviral-based pseudovirus neutralization, VSV-based pseudovirus neutralization, and EHC-83E focus reduction neutralization at 4 weeks after boost. Circles represent individual NHPs, and colors indicate the mRNA-1273 dose as defined in fig. S1C. Dotted lines indicate assay limits of detection. Black and gray lines indicate linear regression and 95% confidence interval, respectively. *r* is Spearman’s correlation coefficient and *P* is the corresponding *P* value.

For analyses of immune correlates, we used data from six different qualified antibody assays performed at the time of SARS-CoV-2 challenge 4 weeks after boost. Anti–S-specific (Fig. 1A) and anti–receptor binding domain (RBD) (Fig. 1B) antibodies, which are critical for mitigating SARS-CoV-2 infection, were assessed with the same techniques used to analyze sera from the phase 3 clinical SARS-CoV-2 vaccine trials and normalized to international units (IU) defined according to World Health Organization (WHO) standards. Binding antibody titers increased compared with control animals in a dose-dependent manner, ranging from a median of 55 to 5800 IU/ml at 0.3 and 100 μg, respectively, for S-specific IgG and 66 to 10,400 IU/ml for RBD-specific IgG (Fig. 1, A and B). There was also a dose-dependent reduction in median ACE2-binding inhibition comparing 100 μg with 1 μg of mRNA-1273 (Fig. 1C), reaching a maximum difference between these two dose groups of 270-fold. In vitro virus–neutralizing activity was determined using three different assays. First, a lentiviral-based D614G pseudovirus neutralization assay revealed a dose-dependent increase in neutralizing activity with a median reciprocal ID_50_ titer of 23,000 at the 100-μg dose and 49 after immunization with 1 μg of mRNA-1273 (Fig. 1D). Vesicular stomatitis virus (VSV)–based pseudovirus (Fig. 1E) and live-virus (Fig. 1F) neutralization followed the same significant trend of dose dependency. Assessments of antibody binding and neutralizing responses were highly correlated with one another, suggesting that mRNA-1273 immunization and elicitation of high-titer S-binding antibody responses predicts functional antibody responses (Fig. 2).

Given the increasing circulation of SAR-CoV-2 variants of concern, some of which have shown a significant reduction in neutralization sensitivity to vaccine-elicited and convalescent sera from subjects previously infected with SARS-CoV-2 (*16*–*20*), we assessed the ability of mRNA-1273 immune NHP sera to neutralize two different SAR-CoV-2 variants of concern. Live viral neutralization of the alpha (B.1.1.7) variant (*21*), which is highly transmissible and has previously been circulating worldwide (*22*), was not appreciably decreased compared with D614G (fig. S3A). The beta (B.1.351) variant, which contains multiple mutations in RBD and NTD and has been reported to show the greatest reduction of neutralization by vaccine-induced sera (*16*, *23*, *24*), exhibited a ninefold reduction compared with D614G in the 100-μg dose group. Nine of 12 animals immunized with 30 or 100 μg of mRNA-1273 had reciprocal ID_50_ titers >100, whereas only one of four animals in the 10-μg dose group had detectable neutralization activity to the beta variant (fig. S3B). The reduction in beta neutralization capacity of mRNA-1273–induced antibodies mirrors what has been previously shown in NHPs and humans using doses of only 30 or 100 μg (*16*, *20*). However, these data further suggest that the mRNA-1273 dose may have a profound effect on eliciting neutralizing antibodies against the beta variant.

### mRNA-1273 vaccination elicits upper and lower airway antibodies

To provide additional immune data on correlates of protection at the site of infection, antibody responses in the lower and upper airways were assessed from BAL and nasal wash samples, respectively, at 2 weeks after boost. There was a dose-dependent increase in BAL and nasal wash S-specific IgG and IgA after two doses of mRNA-1273 (Fig. 1, G to J). BAL S-specific IgG levels after 0.3 and 30 μg of mRNA-1273 ranged from a median of 110 to 280,000 area under the curve (AUC) (Fig. 1G). Nasal wash S-specific IgG titers ranged from 86 to 142,200 AUC (Fig. 1H). The dose-dependent trend for S-specific IgA was similar albeit at lower levels where 30 μg of mRNA-1273 elicited 1400 and 21,300 AUC IgA in BAL (Fig. 1I) and nasal washes (Fig. 1J), respectively. Additionally, upper and lower airway antibody responses correlated with one another and with S-specific IgG and serum-neutralizing activity. The one exception was that there was no correlation with BAL and nasal wash S-specific IgA (fig. S4). Thus, mRNA-1273 vaccination elicits S-specific IgG and IgA antibodies in both the upper and lower airways, which potentially provide immediate protection at the site of infection and limit transmission.

### mRNA-1273 vaccination elicits S-specific CD4 T cell responses

S-specific CD4 and CD8 T cell responses were assessed 2 weeks after boost (fig. S5). There was a direct correlation between dose and the proportion of T helper type 1 (T_H_1) cells [interleukin 2–positive (IL-2^+^), tumor necrosis factor–positive (TNF^+^), and/or interferon-γ–positive (IFN-γ^+^)] among peripheral blood memory CD4 T cells (*P* = 0.006), because all animals in the 30-μg dose group had T_H_1 responses (fig. S6A). By contrast, T_H_2 responses (IL-4^+^and/or IL-5^+^) were low to undetectable in all vaccine dose groups (fig. S6B). By contrast, CD8 T cell responses were also low to undetectable in all vaccine dose groups (fig. S6E). T follicular helper (T_FH_) cells found within secondary lymphoid organs play an important role in B cell responses because of their localization within germinal centers. Thus, we extended our analysis to S-specific T_FH_ cells that express the surface marker CD40L, which causes direct activation of B cells to secrete IgG, and the canonical cytokine IL-21, which is critical for developing robust long-term antibody responses. Most vaccinated animals exhibited S-specific CD40L^+^ T_FH_ cell responses, whose magnitude directly correlated with dose (*P* < 0.001) (fig. S6C). There was also a direct correlation between dose and magnitude of S-specific IL-21 T_FH_ cell responses (*P* = 0.010) (fig. S6D). Thus, in agreement with previous results (*13*, *25*, *26*), mRNA-1273 vaccination induces T_H_1- and T_FH_-skewed CD4 T cell responses.

### mRNA-1273 vaccination protects against upper and lower airway SARS-CoV-2 replication

**Fig. 3.**
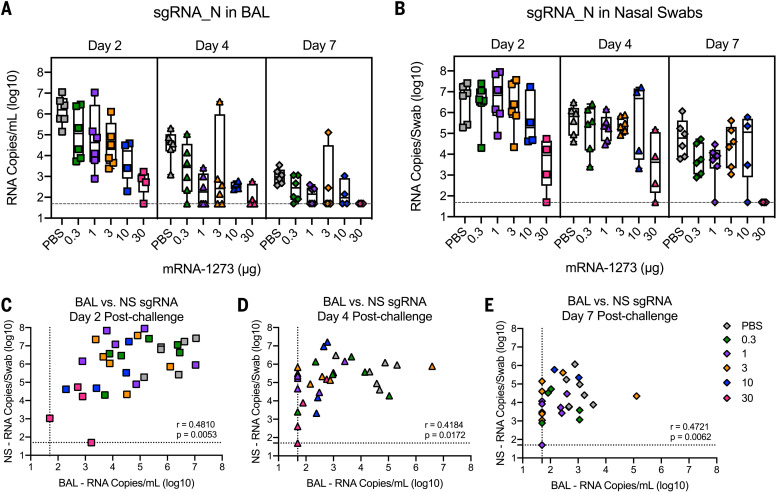
Efficacy of mRNA-1273 against upper and lower respiratory viral replication. (**A** and **B**) Rhesus macaques were immunized and challenged as described in fig. S1C. BAL (A) and nasal swabs (NS) (B) were collected on days 2 (squares), 4 (triangles), and 7 (diamonds) after challenge, and viral replication was assessed by detection of SARS-CoV-2 N-specific sgRNA. In (A) and (B), Boxes and horizontal bars denote the IQR and medians, respectively. Whisker end points are equal to the maximum and minimum values. (**C** to **E**) Correlations shown between BAL and NS sgRNA at days 2 (C), 4 (D), and 7 (E) after challenge are Spearman’s correlation coefficients (*r*) and corresponding *P* values. Symbols represent an individual NHP and may overlap, i.e., *n* = 6 animals plotted at assay limit (dotted line) for both BAL and NS in (E).

To evaluate the impact of mRNA-1273 vaccine dose on protection, animals were challenged 4 weeks after boost with a total dose of 8 × 10^5^ plaque-forming units (PFU) of a highly pathogenic stock of SARS-CoV-2 (USA-WA1/2020) by combined intranasal and intratracheal routes for upper and lower airway infection, respectively (fig. S1C). The challenge dose was chosen to induce viral loads similar to or higher than those detected in nasal secretions of humans after SARS-CoV-2 infection (*27*). The primary efficacy end-point analysis used subgenomic RNA (sgRNA) quantitative reverse transcription polymerase chain reaction for the nucleocapsid (N) gene (Fig. 3) because N sgRNA is the most highly expressed sgRNA species as a result of discontinuous transcription and thus provides greater sensitivity than the envelope (E) gene (fig. S7) (*28*), which is most commonly used in other NHP SARS-CoV-2 vaccine studies (*13*) to quantify replicating virus.

We observed a vaccine dose effect for protection against viral replication in the upper and lower airways. On days 2 and 4 after challenge, there were approximately two and five log_10_ reductions in sgRNA_N in BAL compared with control animals at doses of 1 and 30 μg, respectively (Fig. 3A). Moreover, by day 4 after challenge, most animals vaccinated with 1 μg or higher had low to undetectable sgRNA_E in BAL (fig. S7A). By contrast, the reduction in sgRNA in nasal swabs was primarily limited to animals receiving 30 μg of mRNA-1273 compared with control animals (Fig. 3B and fig. S7B). These data highlight differences in immune responses required for reduction in viral replication for upper and lower airway protection. The virus was more rapidly cleared from BAL compared with nasal swab samples. Although we observed a strong correlation between sgRNA in the upper and lower airways, there was a time-dependent loss of concordance in the correlations with upper and lower airways samples (Fig. 3, C to E), suggesting distinct mechanisms for viral clearance in the two compartments.

### mRNA-1273–vaccinated NHPs have limited virus and inflammation in the lungs

Animals in each of the dose groups were assessed for the presence of virus in the lung and histopathology 7 or 8 days after SARS-CoV-2 challenge. In the control animals, SARS-CoV-2 infection caused patchy, moderate to severe inflammation that often involved the small airways and the adjacent alveolar interstitium, consistent with previous reports (*29*–*31*). Alveolar air spaces occasionally contained inflammatory cell infiltrates, and alveolar capillary septa were moderately thickened. Moreover, moderate and diffuse type II pneumocyte hyperplasia was observed. Multiple pneumocytes in the lung sections from the control group were positive for SARS-CoV-2 viral antigen by immunohistochemistry (IHC) (fig. S8 and table S1). Viral antigen was detected in both control animals but only sporadically across vaccinated animals in various dose groups (table S1). These observations show that naïve NHP develop mild inflammation in the lung >1 week after SARS-CoV-2 infection, and that vaccination limits or completely prevents inflammation or detection of viral antigen in the lung tissue.

### Postchallenge anamnestic antibody responses are increased in low-dose vaccine groups

After SARS-CoV-2 challenge, we assessed antibody responses in blood, BAL, and nasal washes for up to 28 days to determine whether there were anamnestic or primary responses to S or N proteins, respectively (fig. S9). This analysis provides a functional immune assessment of whether the virus detected in the upper and lower airways by PCR after challenge is sufficient to boost vaccine-induced S-specific antibody responses or elicit primary N responses. In sera, there was no postchallenge increase in S-specific (fig. S9A), RBD-specific (fig. S9B), or neutralizing antibodies (fig. S9C) in the 3-, 10-, or 30-μg dose groups. By contrast, at doses below 1 μg, there were increased S-specific (fig. S9A), RBD-specific (fig. S9B), and neutralizing antibody responses (fig. S9C) at day 28 after challenge compared with before challenge. Similar primary S-specific antibody response trends were also apparent with BAL and nasal wash IgG and IgA responses (fig. S10). When prechallenge N-specific IgG responses were compared with postchallenge responses, we only observed seroconversion in the control animals and animals immunized with <3 μg of mRNA-1273 (fig. S9D).

The reduction of viral replication as determined by sgRNA coupled with limited pathology in the lung and no detectable anamnestic S responses or induction of primary responses to N provide three distinct measures suggesting that vaccine-elicited immune responses, particularly at high doses, were protective. To understand this further, and to establish immune correlates of protective immunity, we next explored relationships between immune parameters and viral load.

### Antibody responses correlate with protection against SARS-CoV-2 replication

Before conducting the dose–response study in NHPs (fig. S1C), we prespecified that our analysis to define a potential correlate would focus initially on the relationship between S-specific binding antibodies and sgRNA levels in nasal swabs (NS). Correlations with sgRNA levels in BAL served as an important secondary analysis. The predefined primary hypothesis of the study was that S-specific IgG at 4 weeks after boost at the time of challenge would inversely correlate with viral replication in the NS at day 2 after challenge. The secondary hypotheses were analogous for the relationship between S-specific IgG at 4 weeks after boost and day 2 BAL sgRNA.

**Fig. 4.**
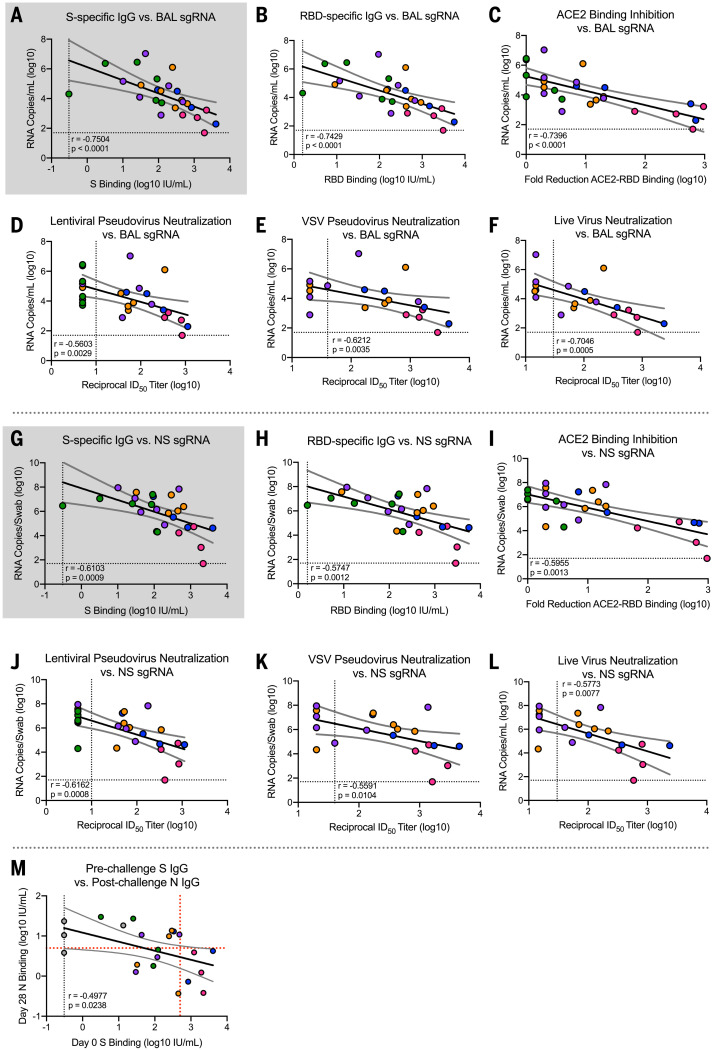
Antibody correlates of protection. Rhesus macaques were immunized and challenged as described in fig. S1C. Plots show correlations between SARS-CoV-2 N-specific sgRNA in BAL [(**A**) to (**F**)] and NS [(**G**) to (**L**)] at day 2 after challenge and before challenge (week 4 after boost) SARS-CoV-2 S-specific IgG [(A) and (G)], RBD-specific IgG [(B) and (H)], ACE2-binding inhibition [(C) and (I)], SARS-CoV-2 lentiviral-based pseudovirus neutralization [(D) and (J)], SARS-CoV-2 VSV-based pseudovirus neutralization [(E) and (K)], and SARS-CoV-2 EHC-83E focus reduction neutralization [(F) and (L)]. Gray shading for S-specific IgG represents the use of this assessment as primary predictor of protection outcome as stated in primary hypothesis. (**M**) Plot showing correlation between prechallenge (week 4 after boost) SARS-CoV-2 S-specific IgG with day 28 postchallenge SARS-CoV-2 N-specific IgG. Circles represent individual NHPs, and colors indicate the mRNA-1273 dose. Dotted lines indicate assay limits of detection. Black and gray lines indicate linear regression and 95% confidence interval, respectively. In (M), a red dotted horizontal line represents N-binding titers of 6, the maximum of all prechallenge values across all groups, and a red dotted vertical line represents a reciprocal S-specific IgG titer of 500, above which none of the animals had day 28 reciprocal N-binding titers >6.

Using a univariate (log) linear model, S-specific IgG at the time of challenge correlated strongly with sgRNA in both the NS (*P* = 0.003, adjusted *R*^2^ = 0.29) (Fig. 4G and table S2) and BAL (*P* = 0.001, adjusted *R*^2^ = 0.35) (Fig. 4A and table S2) at day 2. A 1-log_10_ change in S-specific IgG corresponded to a 1-log_10_ change in sgRNA at day 2 in the NS and a 0.9-log_10_ change in BAL sgRNA at day 2 (table S2). Once the S-specific IgG was included in a multivariate linear model predicting sgRNA, including dose in the model did not substantially increase the adjusted *R*^2^, nor was the coefficient significant (*P* = 0.115 for NS and *P* = 0.214 for BAL). Thus, the dose effect on day 2 sgRNA in NS and BAL appears to be fully captured by the adjustment for S-specific IgG. Moreover, in this model, S-specific IgG meets our prespecified criteria to be considered as a correlate of sgRNA levels in NS and BAL.

Because RBD-specific IgG, ACE2-binding inhibition, pseudovirus neutralization, and live virus neutralization all correlated with S-specific IgG (Fig. 2), we analyzed these endpoints as potential correlates of sgRNA. All six antibody measurements were highly correlated with one other (Fig. 2), with vaccine dose (Fig. 1), and with sgRNA in BAL (Fig. 4, A to F) and NS (Fig. 4, G to L). For all six antibody measurements, the dose was not significantly predictive of sgRNA in the BAL after adjusting for antibody levels (table S2A). For NS, the dose remained significantly predictive after adjusting for VSV-based pseudovirus neutralization and marginally significant after adjusting for live virus neutralization. In addition to S-specific IgG, RBD-specific IgG, ACE2-binding inhibition, and lentiviral-based pseudovirus neutralization appear to meet our criteria for potential correlates of protection. Furthermore, lower and upper airway S-specific antibodies in the BAL and NS negatively correlated with BAL (fig. S11, A and B) and NS sgRNA levels, respectively (fig. S11, C and D).

To assess the robustness of these findings, these analyses were repeated using logistic regression to model the probability that the sgRNA was below a threshold, defined as 10,000 sgRNA copies for BAL and 100,000 sgRNA copies for NS. These thresholds were chosen to lie below all of the sgRNA values in the control animals and within the range of the values for the mRNA-1273–vaccinated animals. The results of these analyses were similar to the primary analyses performed on the (log) linear models. No animal with S-specific IgG >336 IU/ml or >645 IU/ml had BAL (Fig. 4A) or NS (Fig. 4G) sgRNA, respectively, greater than the thresholds defining protection (>10,000 copies/ml BAL or >100,000 copies/swab NS). Finally, no animals with an S-binding titer of >488 IU/ml exhibited higher N-specific primary antibody responses after challenge above the background value at the time of challenge. Consistent with this, there was a strong negative correlation between prechallenge S-specific antibodies and postchallenge N-specific antibodies (Fig. 4M). Additionally, there was limited to no lung pathology or viral antigen detected in animals with <10,000 sgRNA copies/ml in BAL, providing additional evidence that mRNA-1273–vaccinated animals were protected from lower airway disease.

We also examined the correlations between T cell responses and sgRNA and found that CD40L^+^ T_FH_ cell and T_H_1 cell responses were each univariately associated with reduced sgRNA in both BAL and NS. After adjustment for S-specific IgG, neither of these remained significantly associated with sgRNA levels in the BAL, suggesting that these T cell measures do not predict sgRNA independently of the binding antibody measured in BAL. However, IL-21^+^ T_FH_, CD40L^+^ T_FH_, and T_H_1 cell responses remained significantly predictive of sgRNA levels in NS (table S2B). Thus, clearance of virus from BAL and NS have distinct immunological requirements (Fig. 3, C to E).

### Passively transferred mRNA-1273–induced IgG mediates protection against SARS-CoV-2

**Fig. 5.**
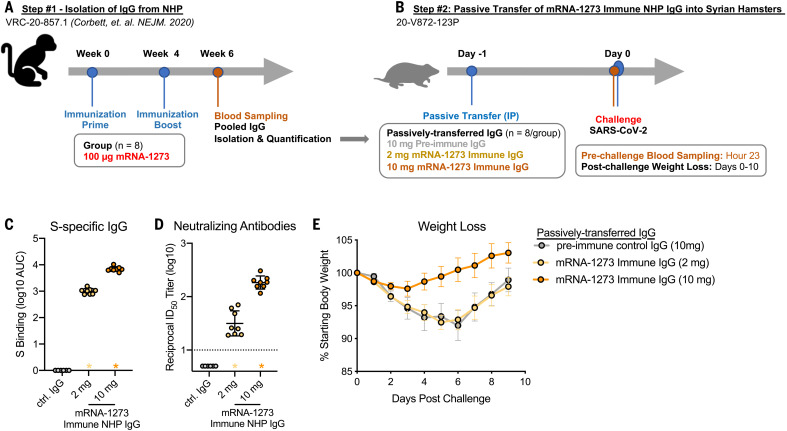
Passive transfer of mRNA-1273 immune NHP IgG into Syrian hamsters. (**A**) Sera were pooled from all NHPs that received 100 μg of mRNA-1273 in a primary vaccination series. (**B**) mRNA-1273 immune NHP IgG (2 μg, yellow; 10 μg, orange) or preimmune NHP IgG (10 μg, gray) was passively transferred to Syrian hamsters (*n* = 8/group) 24 hours before SARS-CoV-2 challenge. (**C** and **D**) Twenty-three hours after immunization, hamsters were bled to quantify circulating S-specific IgG (C) and SARS-CoV-2 pseudovirus-neutralizing antibodies (D). (**E**) After challenge, hamsters were monitored for weight loss. In (C) and (D), circles represent individual NHPs. Bars and error bars represent GMT and geometric SD, respectively. Asterisks at the axis represent animals that did not receive adequate IgG through passive transfer and were thus excluded from weight loss analyses. In (D), the dotted line indicates the neutralization assay limit of detection. In (E), circles and error bars represent means and SEM, respectively.

High-titer antibody responses in blood and upper and lower airways were associated with the rapid control of viral load and lower airway pathology in the lung. This suggested that antibody was the primary immunological mechanism of protection. To directly address whether vaccine-induced antibody was sufficient to mediate protection, IgG was purified from pooled sera from mRNA-1273–immunized NHPs (*13*) (Fig. 5A) and passively transferred into hamsters (Fig. 5B). Hamsters that received preimmune IgG or 2 μg of mRNA-1273–immune IgG showed ~10% weight loss by day 6 (*32*). By contrast, hamsters that received 10 μg of mRNA-1273–immune NHP IgG showed little to no weight loss after challenge (Fig. 5E). Thus, mRNA-1273 immune IgG alone is sufficient to mediate protection from disease in vivo against SARS-CoV-2 infection.

## Discussion

Defining immune correlates of protection is a critical aspect of vaccine development for extending the use of approved vaccines, facilitating the development of new candidate vaccines, and defining potential mechanisms of protection. For SARS-CoV-2, a primary goal of current vaccines is to prevent symptomatic COVID-19. This is achieved by reducing viral load in lower airways, which reduces moderate to severe disease, and reducing viral load in both lower and upper airways, preventing mild disease. An additional benefit of upper airway protection is that limiting nasal carriage of virus will also reduce the risk of transmission. Here, we established that the level of S-specific antibody elicited by mRNA-1273 vaccination correlates with control of upper and lower airway viral replication after SARS-CoV-2 challenge in NHPs and that vaccine-elicited antibodies are sufficient for protection against disease in the highly pathogenic golden Syrian hamster model of SARS-CoV-2 infection.

A key parameter to assess correlates of protection in NHPs is the amount of virus used for challenge. In this study, a challenge dose of 8 × 10^5^ PFUs of a well-characterized, pathogenic SARS-CoV-2 USA-WA1/2020 strain was used to achieve a level of viral replication comparable to or exceeding that observed from nasal swabs of humans with symptomatic infection measured by sgRNA (*33*) or genomic viral RNA (*34*–*36*). The sgRNA levels for N or E of 10^6^ to 10^7^ in control animals at day 2 after challenge are among the highest reported for NHP challenge studies and likely models viral load in humans at the upper end of inoculum size. We used the same qualified antibody binding and pseudovirus neutralization assays for assessing immune responses as in human phase 3 vaccine trials. In addition, the use of WHO standards to report binding titers in international units enables the comparison of immune responses and outcomes with other NHP vaccine studies and benchmarking to human vaccine clinical trials. We have shown that a 10-fold increase in S-binding titers was associated with ~10-fold reductions in viral replication in BAL and NS after challenge. No animal with S-specific IgG >336 IU/ml or >645 IU/ml had BAL or NS sgRNA, respectively, greater than the thresholds defining protection (>10,000 copies/ml BAL or >100,000 copies/swab NS). These reductions in viral replication compared with controls were associated with limited inflammation and viral antigen detection in the lung tissue and appeared to be sufficient to prevent moderate or severe lower airway infection. Even animals in the 1- and 3-μg dose groups, for which the elicited S-specific antibody levels were 81 and 272 IU/ml, respectively, and reciprocal pseudovirus neutralization titers were 49 and 53, respectively, exhibited ~2- to 4-log_10_ less viral replication in BAL compared with the control animals at day 2 after challenge (*34*). Thus, a lower antibody level is needed for the reduction of viral replication in the lower airway than in the upper airway. Moreover, these data highlight how immune correlates may differ in their ability to confer protection against severe disease in the lung versus mild infection in the upper airway, which has potential implications for determining whether higher immune responses will be required to limit transmission and if this will require additional boosting.

The antibody titers required in this high-dose challenge NHP model for a reduction in viral replication may be a conservative estimate for what is required to prevent clinical disease in humans. The strong correlation and proportional changes between S-specific binding titers with serum-neutralizing activity support using easy-to-measure binding titers as the primary metric for defining a correlate of protection in humans, at least for mRNA-based vaccines delivering similar antigens and eliciting similar patterns of immunogenicity.

Mucosal antibody responses are thought to be an important mechanism of protection against a variety of upper respiratory viral infections (*37*–*41*). Both BAL and nasal wash S-specific IgG and IgA were predictive for reducing sgRNA in these compartments. Serum antibody levels were a strong predictor of IgA and IgG responses in BAL and nasal washes, as well as for protection measured by viral replication in these sites. Because mRNA-1273 is administered intramuscularly, localized upper and lower airway antibodies may be transudated from serum, which suggests that serum antibody levels may be a surrogate for BAL and NS antibody levels after mRNA-1273 vaccination.

Additionally, we considered whether the infection could boost vaccine-induced antibodies. There were no anamnestic S-specific responses or increase in N-specific responses in blood or BAL within 28 days after infection in the >3-μg dose groups compared with prechallenge, consistent with these doses inducing higher antibody responses and preventing viral replication. By contrast, there were increased anamnestic S-binding antibody responses in the <1-μg dose groups. Thus, the boosting of vaccine-induced antibodies may occur after upper airway infection in animals that have minimal viral replication in the lower airway. Therefore, the necessity and timing of subsequent vaccine boosting will depend on the goal of the vaccination program. One goal would be to prevent severe disease and lower airway infection while allowing community exposure to provide mucosal immunity from upper airway infection and boosting of the vaccine response; another would be to achieve persistent high-level immunity against mild infection through vaccination to more rapidly reduce transmission.

There are limitations of this study for predicting the real-world human immune correlate(s) of protection against currently circulating virus variants. First, SARS-CoV2 infection of NHPs models mild disease with relatively little lung pathology. Second, this study used the benchmark WA-1 strain for challenge and the results will need to be extended to continually evolving circulating strains and variants. Third, this was a short-term study in which immune responses were assessed at the peak of the postimmunization response and challenge occurred 4 weeks after the boost. Thus, the relationship of serum antibody levels to protection against virus infection or disease using longer challenge intervals after vaccination remains to be determined.

In conclusion, this study establishes the critical role of antibodies as a correlate of protection against SARS-CoV-2 in the NHP model and shows that, for mRNA-1273, S-specific binding antibody is a surrogate marker of protection. Ongoing NHP studies will assess the durability of mRNA-1273–elicited protection and the efficacy of mRNA-1273 vaccination against global SARS-CoV-2 variants. These findings anticipate the correlates analysis comparing virus replication in NS with serum antibody being performed on samples from vaccinated subjects in phase 3 clinical trials who experienced breakthrough infection.

## Materials and Methods

### Preclinical mRNA-1273 mRNA and lipid nanoparticle production

A sequence-optimized mRNA encoding prefusion-stabilized SARS-CoV-2 S-2P protein (*2*, *3*) was synthesized in vitro and formulated as previously reported (*13*, *25*).

### Rhesus macaque model

Animal experiments were performed in compliance with all pertinent National Institutes of Health regulations and approval from the Animal Care and Use Committees of the Vaccine Research Center and Bioqual Inc. (Rockville, MD). Studies were conducted at Bioqual Inc. The experimental details of VRC-20-857.1 (fig. S1A) were published previously (*13*). For VRC-20-857.3 (fig. S1B) or VRC-20-857.4 (fig. S1C), 3- to 8-year-old rhesus macaques of Indian origin were sorted by sex, agem and weight and then stratified into groups. Animals were immunized intramuscularly at week 0 and at week 4 with doses ranging from 0.3 to 30 μg of mRNA-1273 in 1 ml of PBS into the right hindleg. Placebo-control animals were administered an equal volume of PBS. At week 8 (4 weeks after boost), all animals were challenged with a total dose of 8 × 10^5^ PFUs of SARS-CoV-2. The stock of 1.99 × 10^6^ TCID50 or 3×10^6^ PFU/ml SARS-CoV-2 USA-WA1/2020 strain (BEI: NR-70038893) was diluted and administered in 3-ml doses by the intratracheal route and in 1-ml doses by the intranasal route (0.5 ml per nostril). Pre- and postchallenge sample collection are detailed in fig. S1C.

### Passive transfer of purified IgG into golden Syrian hamsters

Sera from NHPs immunized with 100 μg of mRNA-1273 in VRC-20-857.1 (*13*) (fig. S1A) were collected 2 weeks after boost and pooled. Total IgG was purified from pooled sera using Protein G Sepharose 4 Fast Flow resin (Cytiva), according to the manufacturer’s instructions, and quantified by NanoDrop OneC Microvolume UV-Vis Spectrophotometer (ThermoFisher Scientific). The eluted protein dialyzed against PBS, pH 7.4 (Invitrogen), and concentrated to 10 mg/ml using Amicon Ultra centrifugal Filter (Millipore Sigma-Aldrich). Golden Syrian hamsters, aged 6 to 8 weeks, were randomized into groups of eight on the basis of weight, with each group containing a 1:1 ratio of males to females. Two or 10 mg of total mRNA-1273 immune IgG was passively transferred by intraperitoneal injection 1 day before challenge. Immediately before challenge, sera were collected for assessments of S-specific IgG (Fig. 5C) and neutralization titers (Fig. 5D) to confirm the relative antibody responses of the mRNA-1273 immune IgG in the hamsters. Hamsters were inoculated intranasally with 3 × 10^4^ PFUs of USA-WA1/2020 SARS-CoV-2 (BEI, NR-53780) in a final volume of 100 μl split between each nostril. Body weight and clinical observations were made daily after challenge.

### Quantification of SARS-CoV-2 sgRNA

Using previously published methods (*42*), specimens were processed, stored, and subgenomic SARS-CoV-2 E mRNA was quantified with reverse transcription-polymerase chain reaction (RT-PCR). Subgenomic SARS-CoV-2 N mRNA was quantified similarly (forward: 5′-CGATCTCTTGTAGATCTGTTCTC-3′, probe: 5′-FAM- TAACCAGAATGGAGAACGCAGTGGG-BHQ1-3′, reverse: 5′-GGTGAACCAAGACGCAGTAT-3′). The lower limit of quantification was 50 copies.

### Histopathology and IHC

As previously described (*13*), NHP lung tissue sections were stained with hematoxylin and eosin for routine histopathology and a rabbit polyclonal SARS-CoV-2 (GeneTex, GTX135357) for detection of SARS-CoV-2 virus antigen. All samples were evaluated by a board-certified veterinary pathologist.

### 4-Plex meso-scale ELISA

MSD SECTOR plates (MSD) were precoated with SARS-CoV proteins (S-2P, RBD, and N) and a bovine serum albumin control in each well and blocked for 60 min at room temperature (RT) with MSD blocker A solution. Plates were washed with 1× MSD wash buffer, and an MSD reference standard (calibrator), QC test sample (pool of COVID-19 convalescent sera), and heat-inactivated sera were serially diluted and added in duplicate. A reference standard and MSD control sera were added undiluted in triplicates. Samples were incubated at RT for 4 hours with shaking at 1500 rpm. After washing, MSD SULFO-TAG anti-human IgG detection antibody was added for 60 min at RT with shaking. Plates were washed and MSD GOLD read buffer was added. Detection was completed on a MESO Sector S 600 system, and analyses were performed using MSD Discovery Workbench software version 4.0. Calculated ECLIA parameters to measure binding antibody activities included interpolated concentrations or assigned arbitrary units per milliliter read from the standard curve.

### Defining IUs

Recently the arbitrary units were bridged to the WHO international standard and a conversion factor was calculated and confirmed. Parallelism between MSD reference standard and the WHO international standard was established for SARS-CoV proteins (S-2P, RBD, and N). Concentration assignments were performed and then confirmed by MSD and as part of a multisite confirmation study. Sample results reported here have been converted to international units per milliliter. S-specific IgG had a lower limit of detection of 0.3076 IU/ml; RBD-specific IgG had a lower limit of detection of 1.5936 IU/ml.

### ELISA for temporal NHP serum antibodies and hamster serum antibodies

SARS-CoV-2 S-specific IgG in serum was quantified by ELISA, and the methods used were similar to those previously published (*25*). Here, the only amendment to previously published methodology is the resulting data are depicted as end-point titers, which were calculated as the mean serum titer that reached 10× SD using GraphPad Prism version 9.0.2 software.

### Meso-scale ELISA for mucosal antibody responses

Total S-specific IgG and IgA were determined by multi-array ELISA using Meso Scale technology (Meso Scale Discovery, MSD) as previously described (*42*).

### Serum antibody avidity assay

Avidity was assessed using a sodium thiocyanate (NaSCN)–based avidity ELISA against the full-length SARS-CoV-2 S-2P antigen as previously described. The avidity index was calculated using the ratio of IgG binding to S-2P in the absence or presence of NaSCN. The reported avidity index is the average of two independent experiments, each containing duplicate samples.

### ACE2-binding inhibition assay

ACE2-binding inhibition was completed, as previously described (*43*), on 1:40 diluted sera samples using Mesoscale Discovery 384-well, 4-Spot Custom Serology SECTOR plates precoated with SARS-CoV-2 RBD. Binding was detected using SULFO-TAGTM–labeled ACE2. Both reagents were generously supplied by the manufacturer.

### Lentiviral pseudovirus neutralization assay

Pseudotyped lentiviral reporter viruses were produced by the cotransfection of plasmids encoding S proteins from Wuhan-1 strain (GenBank no. MN908947.3) with a D614G mutation, a luciferase reporter, lentivirus backbone, and human transmembrane protease serine 2 (TMPRSS2) genes into HEK293T/17 cells (ATCC CRL-11268) as previously described (*43*). Sera, in duplicate, were tested for neutralizing activity against the D614G pseudoviruses by quantification of luciferase activity in relative light units. Percent neutralization was normalized considering uninfected cells as 100% neutralization and cells infected with pseudovirus alone as 0% neutralization. ID_50_ titers were determined using a log(agonist) versus normalized response (variable slope) nonlinear regression model in GraphPad Prism version 9.0.2 software. The lower limit of quantification was 1:40 ID_50_.

### VSV pseudovirus neutralization assay

To make SARS-CoV-2 pseudotyped recombinant VSV-ΔG-firefly luciferase virus, BHK21/WI-2 cells (Kerafast, EH1011) were transfected with the S plasmid expressing full-length S with a D614G mutation and subsequently infected with VSV∆G-firefly-luciferase as previously described (*44*). Neutralization assays were completed on A549-ACE2-TMPRSS2 cells with serially diluted serum samples as previously described (*16*). The lower limit of quantification was 1:40 ID_50_.

### Focus reduction neutralization test (FRNT)

VeroE6 cells (ATCC, #CRL-1586) were cultured, viral stocks [EHC-083E (D614G SARS-CoV-2) (*18*), B.1.1.7 (PMID 33739374), and B.1.351 (PMID 33972938)] were propagated and titered, and FRNT assays were performed as previously described (*45*). Antibody neutralization was quantified by counting the number of foci for each sample using the Viridot program (*46*). The neutralization titers were calculated as follows: 1 – [ratio of the mean number of foci in the presence of sera and foci at the highest dilution of respective sera sample]. Each specimen was tested in duplicate. The FRNT-50 titers were interpolated using a four-parameter nonlinear regression in GraphPad Prism version 9.0.2.4.3 software. Samples that do not neutralize at the limit of detection at 50% are plotted at 5 and was used for geometric mean calculations.

### Intracellular cytokine staining

Analysis of spike-specific T cell immune responses from cryopreserved peripheral blood mononuclear cells after vaccination was performed as previously described (*13*)*.* The antibody clones, conjugates, commercial source, and analysis purpose for each reagent is shown in table S5. Data were collected on FACS A5 Analyzers (BD Biosciences) and analyzed using FlowJo versions 9.9.6 and 10.7 software (BD Biosciences).

### Correlations and statistical analysis

Graphs show data from individual animals, with dotted lines indicating assay limits of detection. Correlations between antibody measurements and between antibody measurements and dose of vaccine were estimated and tested against zero using Spearman’s nonparametric correlation. Univariate and multivariate linear models were used to evaluate the potential role of antibodies as correlates of protection. Following the Prentice criteria (*47*), we categorized antibody measures as potential correlates of protection if they were significantly univariately predictive of log10 sgRNA as a measure of viral replication or if the addition of log10 dose to the linear model did not significantly improve prediction, as assessed by a likelihood ratio test. Univariate and multivariate linear models were also used to explore the combination of T cell responses and antibody responses in prediction of sgRNA. Comparisons before and after challenge were assessed using paired *t* tests and were based on the last measurement (day 28 after challenge when available; day 14 when not). Intracellular cytokines likely response labels were derived using the MIMOSA (*48*) package. Analyses were performed in R version 4.0.2 and GraphPad Prism version 9.0.2 software.

## Supplementary Material

20210729-1

## Supplementary Materials

### Other Supplementary Material for this manuscript includes the following:

MDAR Reproducibility Checklist

## Notes

View/request a protocol for this paper from *Bio-protocol*.
