# Supplementary material for: Immune correlates of protection by mRNA-1273 vaccine against SARS-CoV-2 in nonhuman primates
Source: Science. 2021 Jul 29;373(6561):eabj0299. doi: 10.1126/science.abj0299 (PMC8449013; doi:10.1126/science.abj0299)
Supplement: 20210729-1 [file science.abj0299.v1.pdf]

Cite as: K. S. Corbett *et al.*, *Science* 10.1126/science.abj0299 (2021).

Immune correlates of protection by mRNA-1273 vaccine against SARS-CoV-2 in nonhuman primates

Kizzmekia S. Corbett[1]†‡, Martha C. Nason[2]†, Britta Flach[1], Matthew Gagne[1], Sarah O'Connell[1], Timothy S. Johnston[1], Shruti N. Shah[1], Venkata Viswanadh Edara[3], Katharine Floyd[3], Lilin Lai[3], Charlene McDanal[4], Joseph R. Francica[1]§, Barbara Flynn[1], Kai Wu[5], Angela Choi[5], Matthew Koch[5], Olubukola M. Abiona[1], Anne P. Werner[1], Juan I. Moliva[1], Shayne F. Andrew[1], Mitzi M. Donaldson[1], Jonathan Fintzi[1], Dillon R. Flebbe[1], Evan Lamb[1], Amy T. Noe[1], Saule T. Nurmukhambetova[1], Samantha J. Provost[1], Anthony Cook[6], Alan Dodson[6], Andrew Faudree[6], Jack Greenhouse[6], Swagata Kar[6], Laurent Pessaint[6], Maciel Porto[6], Katelyn Steingrebe[6], Daniel Valentin[6], Serge Zouantcha[6], Kevin W. Bock[7], Mahnaz Minai[7], Bianca M. Nagata[7], Renee van de Wetering[1], Seyhan Boyoglu-Barnum[1], Kwanyee Leung[1], Wei Shi[1], Eun Sung Yang[1], Yi Zhang[1], John-Paul M. Todd[1], Lingshu Wang[1], Gabriela S. Alvarado[1], Hanne Andersen[6], Kathryn E. Foulds[1], Darin K. Edwards[5], John R. Mascola[1], Ian N. Moore[7], Mark G. Lewis[6], Andrea Carfi[5], David Montefiori[4], Mehul S. Suthar[3,8], Adrian McDermott[1], Mario Roederer[1], Nancy J. Sullivan[1], Daniel C. Douek[1], Barney S. Graham[1]*, Robert A. Seder[1]*

[1]Vaccine Research Center, National Institute of Allergy and Infectious Diseases, National Institutes of Health, Bethesda, MD 20892, USA. [2]Biostatistics Research Branch, Division of Clinical Research, National Institute of Allergy and Infectious Diseases, National Institutes of Health, Bethesda, MD 20892, USA. [3]Center for Childhood Infections and Vaccines of Children's Healthcare of Atlanta, Department of Pediatrics, Department of Microbiology and Immunology, Emory Vaccine Center, Emory University, Atlanta, GA 30322, USA. [4]Department of Surgery, Duke University Medical Center, Durham, NC 27708, USA. [5]Moderna Inc., Cambridge, MA 02139, USA. [6]Bioqual Inc., Rockville, MD 20850, USA. [7]Infectious Disease Pathogenesis Section, National Institute of Allergy and Infectious Diseases, National Institutes of Health, Bethesda, MD 20892, USA. [8]Department of Microbiology and Immunology, Atlanta, GA 30329, USA.

†These authors contributed equally to this work.

‡Present address: Department of Immunology and Infectious Diseases, Harvard T. H. Chan School of Public Health, Boston, MA 02124, USA.

§Present address: AstraZeneca, Gaithersburg, MD 20878, USA..

*Corresponding author. Email: rseder@mail.nih.gov (R.A.S.); bgraham@nih.gov (B.S.G.)

Immune correlates of protection can be used as surrogate endpoints for vaccine efficacy. Here, nonhuman primates (NHPs) received either no vaccine or doses ranging from 0.3 to 100 µg of SARS-CoV-2 vaccine, mRNA-1273. mRNA-1273 vaccination elicited robust circulating and mucosal antibody responses in a dose-dependent manner. Viral replication was significantly reduced in bronchoalveolar lavages and nasal swabs following SARS-CoV-2 challenge in vaccinated animals and most strongly correlated with levels of anti-S antibody and neutralizing activity. Lower antibody levels are needed for reduction of viral replication in the lower airway than in the upper airway. Passive transfer of mRNA-1273-induced IgG to naïve hamsters was sufficient to mediate protection. Thus, mRNA-1273 vaccine-induced humoral immune responses are a mechanistic correlate of protection against SARS-CoV-2 in NHPs.

Severe acute respiratory syndrome coronavirus 2 (SARS-CoV-2), the causative agent of coronavirus disease 2019 (COVID-19), has resulted in more than 180 million infections and 4 million deaths worldwide as of July 13, 2021 (*1*). Mass vaccination offers the most efficient public health intervention to control the pandemic. Two mRNA-based vaccines—Moderna's mRNA-1273 and Pfizer/BioNTech's BNT162b2—both produce a stabilized version of the spike glycoprotein (*2*, *3*), show >94% efficacy against symptomatic COVID-19 in interim Phase 3 analyses (*4*, *5*), and are currently being administered globally. Several other vaccines have shown 60 to 80% efficacy against COVID-19 in Phase 3 trials (*6*, *7*) and a number of candidate vaccines are in earlier stages of clinical development (*8*). A critical issue for optimizing the use of COVID-19 vaccines is defining an immune correlate of protection. This predictor of vaccine efficacy can be used to inform potential dose reduction, advance approval of other vaccine candidates in lieu of Phase 3 efficacy data, extend indications for use to other age groups, and provide insights into durability of protection, necessity for booster vaccination and immune mechanisms of protection (*9*).

The nonhuman primate (NHP) model has been used to demonstrate immunogenicity and protective efficacy against SARS-CoV-2 with several vaccine candidates (*10*–*13*). The high level of protection achieved with mRNA vaccines in NHPs using clinically relevant dose regimens parallels results from human trials. This model exhibits upper and lower airway infection and pathology similar to clinical presentations

of mild COVID-19 in humans (*14*). Although immune responses associated with protection after primary infection have been assessed in NHPs (*15*), there are no studies to date that have specifically defined immune correlates of protection in upper and lower airways after vaccination with any COVID-19 vaccine approved for use in humans.

We used immunogenicity and protection assessments from our previous NHP mRNA-1273 vaccine study (*13*) to test the hypothesis that serum antibody serves as an immune correlate of protection. Here, in a dose de-escalation study, we evaluated how multiple measurements of humoral and cellular immunity correlate with the reduction of viral replication in the upper and lower airway following challenge. Antibody analyses were also performed on bronchoalveolar lavages (BAL) and nasal washes after vaccination to assess correlates relevant for clinical disease and transmission, respectively. Finally, we demonstrated the ability of passively transferred IgG from mRNA-immunized NHP to protect against SARS-CoV-2 infection of animals. Thus, this work delineates spike (S)-specific antibodies as a correlate of protection, highlights the ability of localized mucosal antibodies to control upper and lower airway viral replication, and confirms that mRNA-1273-induced IgG is sufficient for protection against SARS-CoV-2 infection in preclinical models.

Results

mRNA-1273 vaccination elicits antibody responses in a dose-dependent manner

We previously demonstrated dose-dependency of serum antibody responses in NHP following vaccination with 10 or 100 μg of mRNA-1273, with high-level protection against SARS-CoV-2 challenge in both dose groups (fig. S1A) (*13*). These and other immunogenicity outcomes from an additional NHP study in which animals were vaccinated with 30 μg of mRNA-1273 (fig. S1B) were used to design a study to evaluate immune correlates of protection following mRNA-1273 vaccination in the current study (fig. S1C). Doses of mRNA-1273 ranging from 0.3 to 30 μg were administered in the standard clinical regimen at weeks 0 and 4 to generate a range of immune responses and protective outcomes.

We first assessed temporal serum S-specific antibody binding, avidity, and neutralization responses post-prime and -boost. Consistent with our previous report (*13*), S-specific binding antibody (*2*, *3*) was increased over baseline after each immunization, reaching 7900 and 64,000 median reciprocal endpoint titers by 4 weeks post-prime and -boost, respectively, following immunization with 30 μg of mRNA-1273 (fig. S2A). There was an 8–10-fold increase in S-specific binding antibodies after the boost in all dose groups, except for the 0.3-μg dose for which boosting elicited 300-fold more S-specific antibodies. The boost improved not only antibody quantity, but also binding strength as shown by S-specific antibody avidity that increased twofold after the boost in all vaccine groups except for the 0.3-μg dose, with no differences between the vaccine groups (fig. S2B). Neutralizing antibody responses against D614G, a dominant variant circulating worldwide, were detectable in the 10- and 30-μg dose groups as early as 4 weeks post-prime (30 μg, median reciprocal ID_{50}=76), The boost elicited neutralizing antibodies in all but the lowest (0.3 μg) dose groups, and increased by ~1 log_{10} post-boost in the highest dose group (fig. S2C).

For analyses of immune correlates, we used data from six different qualified antibody assays performed at the time of SARS-CoV-2 challenge, 4 weeks post-boost. Anti-S-specific (Fig. 1A) and anti-receptor binding domain (RBD) antibodies, which are critical for mitigating SARS-CoV-2 infection (Fig. 1B), were assessed utilizing the same techniques employed to analyze serum from the Phase 3 clinical SARS-CoV-2 vaccine trials and normalized to international units (IU) defined according to World Health Organization (WHO) standards. Binding antibody titers increased compared to control animals in a dose-dependent manner ranging from a median of 55 to 5800 IU/ml at 0.3 and 100 μg, respectively, for S-specific IgG, and 66 to 10,400 IU/ml for RBD-specific IgG (Fig. 1, A and B). There was also a dose-dependent reduction in median ACE2 binding inhibition comparing 100 μg to 1 μg of mRNA-1273 (Fig. 1C), reaching a maximum difference between these two dose groups of 270-fold. In vitro virus neutralizing activity was determined using three different assays. First, a lentiviral-based D614G pseudovirus neutralization assay revealed a dose-dependent increase in neutralizing activity with a median reciprocal ID_{50} titer of 23,000 at the 100-μg dose and 49 following immunization with 1 μg of mRNA-1273 (Fig. 1D). VSV-based pseudovirus (Fig. 1E) and live-virus (Fig. 1F) neutralization followed the same significant trend of dose dependency. Assessments of antibody binding and neutralizing responses were highly correlated with one another, suggesting mRNA-1273 immunization and elicitation of high titer S-binding antibody responses predicts functional antibody responses (Fig. 2).

Given the increasing circulation of SAR-CoV-2 variants of concern, some of which have shown a significant reduction in neutralization sensitivity to vaccine-elicited and convalescent sera from subjects previously infected with SARS-CoV-2 (*16*–*20*), we assessed the ability of mRNA-1273 immune NHP sera to neutralize two different SAR-CoV-2 variants of concern. Live viral neutralization of the alpha (B.1.1.7) variant (*21*), which is highly transmissible and has previously been circulating worldwide (*22*), was not appreciably decreased compared to D614G (fig. S3A). The beta (B.1.351) variant, which contains multiple mutations in RBD and NTD and has been reported to show the greatest reduction of neutralization by vaccine-induced sera (*16*, *23*, *24*), exhibited a ninefold reduction compared to D614G in the 100-μg-dose group.

Notably, 9 of 12 animals immunized with 30 or 100 μg of mRNA-1273 had reciprocal ID_{50} titers >100, whereas only 1 of 4 animals in the 10-μg-dose group had detectable neutralization activity to the beta variant (fig. S3B). The reduction in beta neutralization capacity of mRNA-1273-induced antibodies mirrors what has been previously shown in NHPs and humans using only 30 or 100 μg (*16*, *20*). However, these data further suggest that the mRNA-1273 dose may have a profound effect on eliciting neutralizing antibodies against the beta variant.

mRNA-1273 vaccination elicits upper and lower airway antibodies

To provide additional immune data on correlates of protection at the site of infection, antibody responses in the lower and upper airways were assessed from BAL and nasal wash samples, respectively, at 2 weeks post-boost. There was a dose-dependent increase in BAL and nasal wash S-specific IgG and IgA following two doses of mRNA-1273 (Fig. 1, G to J). BAL S-specific IgG levels following 0.3 and 30 μg of mRNA-1273 ranged from a median of 110 to 280,000 area under the curve (AUC) (Fig. 1G). Nasal wash S-specific IgG titers ranged from 86 to 142,200 AUC (Fig. 1H). The dose-dependent trend for S-specific IgA was similar albeit at lower levels where 30 μg of mRNA-1273 elicited 1400 and 21,300 AUC IgA in BAL (Fig. 1I) and nasal washes (Fig. 1J), respectively. Additionally, upper and lower airway antibody responses correlated with one another and with S-specific IgG and serum neutralizing activity. The one exception was that there was no correlation with BAL and nasal wash S-specific IgA (fig. S4). Thus, mRNA-1273 vaccination elicits S-specific IgG and IgA antibodies in both the upper and lower airways, which potentially provide immediate protection at the site of infection and limit transmission.

mRNA-1273 vaccination elicits S-specific CD4 T cell responses

S-specific CD4 and CD8 T cell responses were assessed 2 weeks post-boost (fig. S5). There was a direct correlation between dose and the proportion of type 1-helper (Th1) cells ($IL\text{-}2^+$, TNF^+, and/or $IFN\text{-}\gamma^+$) among peripheral blood memory CD4 T cells (P=0.006), as all animals in the 30-μg-dose group had Th1 responses (fig. S6A). By contrast, type 2 T helper (Th2) responses ($IL\text{-}4^+$and/or $IL\text{-}5^+$) were low to undetectable in all vaccine dose groups (fig. S6B). By contrast, CD8 T cell responses were also low to undetectable in all vaccine dose groups (fig. S6E). T follicular helper (Tfh) cells found within secondary lymphoid organs play an important role in B cell-responses due to their localization within germinal centers. Thus, we extended our analysis to S-specific Tfh cells that express the surface marker CD40L, which causes direct activation of B cells to secrete IgG, and the canonical cytokine IL-21, which is critical for developing robust long-term antibody responses. Most vaccinated animals exhibited S-specific $CD40L^+$ Tfh cell responses, whose magnitude directly correlated with dose ($P<0.001$) (fig. S6C). There was also a direct correlation between dose and magnitude of S-specific IL-21 Tfh cell responses ($P=0.010$) (fig. S6D). Thus, in agreement with previous results (*13*, *25*, *26*), mRNA-1273 vaccination induces Th1- and Tfh-skewed CD4 T cell responses.

mRNA-1273 vaccination protects against upper and lower airway SARS-CoV-2 replication

To evaluate the impact of mRNA-1273 vaccine dose on protection, animals were challenged 4 weeks post-boost with a total dose of 8×10^5 PFU of a highly pathogenic stock of SARS-CoV-2 (USA-WA1/2020) by combined intranasal and intratracheal routes for upper and lower airway infection, respectively (fig. S1C). The challenge dose was chosen to induce viral loads similar to or higher than those detected in nasal secretions of humans following SARS-CoV-2 infection (*27*). The primary efficacy endpoint analysis used subgenomic RNA (sgRNA) qRT-PCR for the nucleocapsid (N) gene (Fig. 3) since N sgRNA is the most highly expressed sgRNA species as a result of discontinuous transcription and thus provides greater sensitivity than the envelope (E) gene (fig. S7) (*28*), which is most commonly used in other NHP SARS-CoV-2 vaccine studies (*13*) to quantify replicating virus.

We observed a vaccine dose effect for protection against viral replication in the upper and lower airways. On days 2 and 4 post challenge, there were approximately two and five $\log_{10}$ reductions in sgRNA_N in BAL compared to control animals at doses of 1 μg and 30 μg, respectively (Fig. 3A). Moreover, by day 4 post-challenge, most animals vaccinated with 1 μg or higher had low to undetectable sgRNA_E in BAL (fig. S7A). By contrast, the reduction in sgRNA in nasal swabs was primarily limited to animals receiving 30 μg of mRNA-1273 as compared to control animals (Fig. 3B and fig. S7B). These data highlight differences in immune responses required for reduction in viral replication for upper and lower airway protection. The virus was more rapidly cleared from BAL compared to nasal swab samples. Although we observed a strong correlation between sgRNA in the upper and lower airways, there was a time-dependent loss of concordance in the correlations with upper and lower airways samples (Fig. 3, C to E), suggesting distinct mechanisms for viral clearance in the two compartments.

mRNA-1273-vaccinated NHP have limited virus and inflammation in the lungs

Animals in each of the dose groups were assessed for the presence of virus in the lung and histopathology 7- or 8-days post SARS-CoV-2 challenge. In the control animals, SARS-CoV-2 infection caused patchy moderate-to-severe

inflammation that often involved the small airways and the adjacent alveolar interstitium, consistent with previous reports (*29–31*). Alveolar air spaces occasionally contained inflammatory cell infiltrates and alveolar capillary septa were moderately thickened. Moreover, moderate and diffuse type II pneumocyte hyperplasia was observed. Multiple pneumocytes in the lung sections from the control group were positive for SARS-CoV-2 viral antigen by immunohistochemistry (IHC) (fig. S8 and table S1). Viral antigen was detected in both control animals but only sporadically across vaccinated animals in various dose groups (table S1). These observations show that naïve NHP develop mild inflammation in the lung over 1 week following SARS-CoV-2 infection and that vaccination limits or completely prevents inflammation or detection of viral antigen in the lung tissue.

Post-challenge anamnestic antibody responses are increased in low-dose vaccine groups

Following SARS-CoV-2 challenge, we assessed antibody responses in blood, BAL, and nasal washes for up to 28 days to determine if there were anamnestic or primary responses to S or N proteins, respectively (fig. S9). This analysis provides a functional immune assessment of whether the virus detected in the upper and lower airways by PCR following challenge is sufficient to boost vaccine-induced S-specific antibody responses or elicit primary N responses. In sera, there was no post-challenge increase in S-specific (fig. S9A), RBD-specific (fig. S9B), or neutralizing antibodies (fig. S9C) in the 3-, 10-, or 30-μg-dose groups. By contrast, at doses below 1 μg, there were increased S-specific (fig. S9A), RBD-specific (fig. S9B), and neutralizing antibody responses (fig. S9C) at day 28 post-challenge compared to pre-challenge. Similar primary S-specific antibody response trends were also apparent with BAL and nasal wash IgG and IgA responses (fig. S10). When pre-challenge N-specific IgG responses were compared to post-challenge responses, we only observed seroconversion in the control animals and animals immunized with <3 μg of mRNA-1273 (fig. S9D).

The reduction of viral replication as determined by sgRNA coupled with limited pathology in the lung and no detectable anamnestic S responses or induction of primary responses to N provide three distinct measures suggesting that vaccine-elicited immune responses, particularly at high doses, were protective. To understand this further, and to establish immune correlates of protective immunity, we next explored relationships between immune parameters and viral load.

Antibody responses correlate with protection against SARS-CoV-2 replication

Before conducting the dose-response study in NHP (fig. S1C), we prespecified that our analysis to define a potential correlate would focus initially on the relationship between S-specific binding antibodies and sgRNA levels in nasal swabs (NS). Correlations with sgRNA levels in BAL served as an important secondary analysis. The predefined primary hypothesis of the study was that S-specific IgG at 4 weeks post-boost at the time of challenge would inversely correlate with viral replication in the NS at day 2 post-challenge. The secondary hypotheses were analogous for the relationship between S-specific IgG at 4 weeks post-boost and day 2 BAL sgRNA.

Using a univariate (log) linear model, S-specific IgG at the time of challenge correlated strongly with sgRNA in both the NS (P=0.003, adjusted R^2=0.29) (Fig. 4G and table S2) and BAL (P=0.001, adjusted R^2=0.35) (Fig. 4A and table S2) at day 2. A one-$\log_{10}$ change in S-specific IgG corresponded to a one-$\log_{10}$ change in sgRNA at day 2 in the NS and a 0.9-$\log_{10}$ change in BAL sgRNA at day 2 (table S2). Once the S-specific IgG was included in a multivariate linear model predicting sgRNA, including dose in the model did not substantially increase the adjusted R^2, nor was the coefficient significant (P=0.115 for NS and P=0.214 for BAL). Thus, the dose effect on day 2 sgRNA in NS and BAL appears to be fully captured by the adjustment for S-specific IgG. Moreover, in this model, S-specific IgG meets our prespecified criteria to be considered as a correlate of sgRNA levels in NS and BAL.

Since RBD-specific IgG, ACE2-binding inhibition, pseudovirus neutralization, and live-virus neutralization all correlated with S-specific IgG (Fig. 2), we analyzed these endpoints as potential correlates of sgRNA. All six antibody measurements were highly correlated with one other (Fig. 2), with vaccine dose (Fig. 1), and with sgRNA in BAL (Fig. 4, A to F) and NS (Fig. 4, G to L). For all six antibody measurements, the dose was not significantly predictive of sgRNA in the BAL after adjusting for antibody levels (table S2A). For NS, the dose remained significantly predictive after adjusting for VSV-based pseudovirus neutralization and marginally significant after adjusting for live-virus neutralization. In addition to S-specific IgG, RBD-specific IgG, ACE2-binding inhibition, and lentiviral-based pseudovirus neutralization appear to meet our criteria for potential correlates of protection. Furthermore, lower and upper airway S-specific antibodies in the BAL and NS negatively correlated with BAL (fig. S11, A and B) and NS sgRNA levels, respectively (fig. S11, C and D).

To assess the robustness of these findings, these analyses were repeated using logistic regression to model the probability that the sgRNA was below a threshold, defined as 10,000 sgRNA copies for BAL and 100,000 sgRNA copies for NS. These thresholds were chosen to lie below all of the sgRNA values in the control animals and within the range of the values for the mRNA-1273-vaccinated animals. The results of these analyses were similar to the primary analyses performed on the (log) linear models. No animal with S-specific IgG >336 IU/ml or >645 IU/ml had BAL (Fig. 4A) or NS (Fig. 4G) sgRNA, respectively, greater than the thresholds

defining protection (>10,000 copies/ml BAL or >100,000 copies/swab NS). Finally, no animals with an S-binding binding titer of >488 IU/ml exhibited higher N-specific primary antibody responses post-challenge above the background value at the time of challenge. Consistent with this, there was a strong negative correlation between pre-challenge S-specific antibodies and post-challenge N-specific antibodies (Fig. 4M). Additionally, there was limited-to-no lung pathology or viral antigen detected in animals with <10,000 sgRNA copies/ml in BAL, providing additional evidence that mRNA-1273-vaccinated animals were protected from lower airway disease.

We also examined the correlations between T cell responses and sgRNA and found that CD40L$^+$ Tfh cell and Th1 cell responses were each univariately associated with reduced sgRNA in both BAL and NS. After adjustment for S-specific IgG, neither of these remained significantly associated with sgRNA levels in the BAL, suggesting that these T cell measures do not predict sgRNA independently of the binding antibody measured in BAL. However, IL-21$^+$ Tfh cell, CD40L$^+$ Tfh cell, and Th1 cell responses remained significantly predictive of sgRNA levels in NS (table S2B). Thus, clearance of virus from BAL and NS have distinct immunological requirements (Fig. 3, C to E).

Passively transferred mRNA-1273-induced IgG mediates protection against SARS-CoV-2

High-titer antibody responses in blood and upper and lower airways were associated with the rapid control of viral load and lower airway pathology in the lung. This suggested that antibody was the primary immunological mechanism of protection. To directly address whether vaccine-induced antibody was sufficient to mediate protection, IgG was purified from pooled sera from mRNA-1273-immunized NHPs (*13*) (Fig. 5A) and passively transferred into hamsters (Fig. 5B). Hamsters that received pre-immune IgG or 2 μg of mRNA-1273-immune IgG showed ~10% weight loss by day 6 (*32*). By contrast, hamsters that received 10 μg of mRNA-1273-immune NHP IgG showed little-to-no weight loss post-challenge (Fig. 5E). Thus, mRNA-1273 immune IgG alone is sufficient to mediate protection from disease in vivo against SARS-CoV-2 infection.

Discussion

Defining immune correlates of protection is a critical aspect of vaccine development for extending the use of approved vaccines and facilitating the development of new candidate vaccines, as well as defining potential mechanisms of protection. For SARS-CoV-2, a primary goal of current vaccines is to prevent symptomatic COVID-19. This is achieved by reducing viral load in lower airways, which reduces moderate-to-severe disease, and reducing viral load in both lower and upper airways preventing mild disease. An additional benefit of upper airway protection is that limiting nasal carriage of virus will also reduce the risk of transmission. Here, we established that the level of S-specific antibody elicited by mRNA-1273 vaccination correlates with control of upper and lower airway viral replication following SARS-CoV-2 challenge in NHP and that vaccine-elicited antibodies are sufficient for protection against disease in the highly pathogenic golden Syrian hamster model of SARS-CoV-2 infection.

A key parameter to assess correlates of protection in NHP is the amount of virus used for challenge. In this study, a challenge dose of 8×10^5 PFU of a well characterized, pathogenic SARS-CoV-2 USA-WA1/2020 strain was used to achieve a level of viral replication comparable to or exceeding that observed from nasal swabs of humans with symptomatic infection, measured by sgRNA (*33*) or genomic vRNA (*34*–*36*). The sgRNA levels for N or E of 10^6-10^7 in control animals at day 2 post-challenge are among the highest reported for NHP challenge studies and likely models viral load in humans at the upper end of inoculum size. We used the same qualified antibody binding and pseudovirus neutralization assays for assessing immune responses as in human Phase 3 vaccine trials. Additionally, the use of WHO standards to report binding titers in IU enables the comparison of immune responses and outcomes with other NHP vaccine studies and benchmarking to human vaccine clinical trials. We show that a 10-fold increase in S-binding titers was associated with approximately 10-fold reductions in viral replication in BAL and NS post-challenge. No animal with S-specific IgG >336 IU/ml or >645 IU/ml had BAL or NS sgRNA, respectively, greater than the thresholds defining protection (>10,000 copies/ml BAL or >100,000 copies/swab NS). These reductions in viral replication compared to controls were associated with limited inflammation and viral antigen detection in the lung tissue and appear to be sufficient to prevent moderate or severe lower airway infection. Notably, even animals in the 1- and 3-μg-dose groups, for which the elicited S-specific antibody levels were 81 and 272 IU/ml and reciprocal pseudovirus neutralization titers of 49 and 53, exhibited ~2–4 $\log_{10}$ less viral replication in BAL compared to the control animals at day 2 post-challenge (*34*). Thus, a lower antibody level is needed for the reduction of viral replication in the lower airway than in the upper airway. Moreover, these data highlight how immune correlates may differ in their ability to confer protection against severe disease in the lung versus mild infection in the upper airway, which has potential implications for whether higher immune responses will be required to limit transmission and if this will require additional boosting.

The antibody titers required in this high-dose challenge NHP model for a reduction in viral replication may be a conservative estimate for what is required to prevent clinical disease in humans. The strong correlation and proportional changes between S-specific binding titers with serum

neutralizing activity support using easy-to-measure binding titers as the primary metric for defining a correlate of protection in humans, at least for mRNA-based vaccines delivering similar antigens and eliciting similar patterns of immunogenicity.

Mucosal antibody responses are thought to be an important mechanism of protection against a variety of upper respiratory viral infections (*37–41*). Both BAL and nasal wash S-specific IgG and IgA were predictive for reducing sgRNA in these compartments. Serum antibody levels were a strong predictor of IgA and IgG responses in BAL and nasal washes as well as for protection measured by viral replication in these sites. As mRNA-1273 is administered via intramuscular delivery, localized upper and lower airway antibodies may be transudated from serum, which suggests that serum antibody levels may be a surrogate for BAL and NS antibody levels following mRNA-1273 vaccination.

Additionally, we considered whether the infection could boost vaccine-induced antibodies. There were no anamnestic S-specific responses or increase in N-specific responses in blood or BAL within 28 days post infection in the >3-μg-dose groups compared to pre-challenge, consistent with these doses inducing higher antibody responses and preventing viral replication. By contrast, there were increased anamnestic S-binding antibody responses in the <1-μg-dose groups. Thus, the boosting of vaccine-induced antibodies may occur following upper airway infection in animals that have minimal viral replication in the lower airway. Consequently, the necessity and timing of subsequent vaccine boosting will depend on the goal of the vaccination program. One goal would be to prevent severe disease and lower airway infection while allowing community exposure to provide mucosal immunity from upper airway infection and boosting of the vaccine response. Another would be to achieve persistent high-level immunity against mild infection through vaccination to more rapidly reduce transmission.

There are limitations of this study for predicting the real-world human immune correlate(s) of protection against currently circulating virus variants. First, SARS-CoV2 infection of NHPs models mild disease with relatively little lung pathology. Second, this study used the benchmark WA-1 strain for challenge and the results will need to be extended to continually evolving circulating strains and variants. Third, this was a short-term study in which immune responses were assessed at the peak of the post-immunization response and challenge occurred 4 weeks after the boost. Thus, the relationship of serum antibody levels to protection against virus infection or disease using longer challenge intervals after vaccination remains to be determined.

In conclusion, this study establishes the critical role of antibodies as a correlate of protection against SARS-CoV-2 in the NHP model and that, for mRNA-1273, S-specific binding antibody is a surrogate marker of protection. Ongoing NHP studies will assess durability of mRNA-1273-elicited protection and efficacy of mRNA-1273 vaccination against global SARS-CoV-2 variants. These findings anticipate the correlates analysis comparing virus replication in NS with serum antibody being performed on samples from vaccinated subjects in Phase 3 clinical trials who experienced breakthrough infection.

Materials and Methods

Preclinical mRNA-1273 mRNA and lipid nanoparticle production

A sequence-optimized mRNA encoding prefusion-stabilized SARS-CoV-2 S-2P protein (*2*, *3*) was synthesized in vitro and formulated as previously reported (*13*, *25*).

Rhesus macaque model

Animal experiments were carried out in compliance with all pertinent US National Institutes of Health regulations and approval from the Animal Care and Use Committees of the Vaccine Research Center and Bioqual, Inc. (Rockville, MD). Studies were conducted at Bioqual, Inc. The experimental details of VRC-20-857.1 (fig. S1A) are previously published (*13*). For VRC-20-857.3 (fig. S1B) or VRC-20-857.4 (fig. S1C), 3–8-year-old rhesus macaques of Indian origin were sorted by sex, age and weight and then stratified into groups. Animals were immunized intramuscularly (IM) at week 0 and week 4 with doses ranging from 0.3–30 μg of mRNA-1273 in 1 ml of PBS into the right hindleg. Placebo-control animals were administered an equal volume of PBS. At week 8 (4 weeks post-boost), all animals were challenged with a total dose of 8×10^5 PFU SARS-CoV-2. The stock of 1.99×10^6 TCID50 or 3×10^6 PFU/ml SARS-CoV-2 USA-WA1/2020 strain (BEI: NR-70038893) was diluted and administered in 3 ml by the intratracheal route and 1 ml by the intranasal route (0.5 ml per nostril). Pre- and post-challenge sample collection is detailed in fig. S1C.

Passive transfer of purified IgG into golden Syrian hamsters

Sera from NHP immunized with 100 μg of mRNA-1273 in VRC-20-857.1 (*13*) (fig. S1A) were collected 2 weeks post-boost and pooled. Total IgG was purified from pooled sera using Protein G Sepharose 4 Fast Flow resin (Cytiva), according to manufacturer's instructions, and quantified by NanoDrop OneC Microvolume UV-Vis Spectrophotometer (Thermo Scientific). The eluted protein dialyzed against PBS pH 7.4 (GIBCO) and concentrated to 10 mg/ml using Amicon® Ultra centrifugal Filter (Millipore Sigma). Golden Syrian hamsters, aged 6–8 weeks, were randomized into groups of eight based on weight, with each group containing a 1:1 ratio of males to females. Two or ten milligrams of total mRNA-1273 immune

IgG was passively transferred by intraperitoneal (IP) injection 1 day prior to challenge. Immediately prior to challenge, sera were collected for assessments of S-specific IgG (Fig. 5C) and neutralization titers (Fig. 5D) to confirm the relative antibody responses of the mRNA-1273 immune IgG in the hamsters. Hamsters were inoculated intranasally with 3×10^4 PFU USA-WA1/2020 SARS-CoV-2 (BEI, NR-53780) in a final volume of 100 μl and split between each nostril. Body weight and clinical observations were made daily post-challenge.

Quantification of SARS-CoV-2 subgenomic RNA (sgRNA)

Using previously published methods, specimens were processed and stored, and subgenomic SARS-CoV-2 E mRNA was quantified via reverse transcription-polymerase chain reaction (RT-PCR). Subgenomic SARS-CoV-2 N mRNA was quantified similarly (Forward: 5′-CGATCTCTTGTAGATCTGTT CTC-3′, Probe: 5′-FAM- TAACCAGAATGGAGAACGCAGT GGG-BHQ1-3′, Reverse: 5′-GGTGAACCAAGACGCAGTAT-3′). The lower limit of quantification was 50 copies.

Histopathology and immunohistochemistry (IHC)

As previously described (*13*), NHP lung tissue sections were stained with hematoxylin and eosin (H&E) for routine histopathology and a rabbit polyclonal SARS-CoV-2 (GeneTex, GTX135357) for detection of SARS-CoV-2 virus antigen. All samples were evaluated by a board-certified veterinary pathologist.

4-Plex meso-scale ELISA

MSD SECTOR® plates (MSD) were precoated with SARS-CoV proteins (S-2P, RBD, and N) and a bovine serum albumin (BSA) control in each well and blocked for 60 min at room temperature (RT) with MSD blocker A solution. Plates were washed with 1X MSD Wash Buffer, and an MSD reference standard (calibrator), QC test sample (pool of COVID-19 convalescent sera), and heat-inactivated sera were serially diluted and added in duplicate. A reference standard and MSD control sera were added undiluted in triplicates. Samples were incubated at RT for 4 hours shaking at 1500 rpm. Following washing, MSD SULFO-TAG anti-human IgG detection antibody was added for 60 min at RT with shaking. Plates were washed, and MSD GOLD read buffer was added. Detection was completed on a MESO Sector S 600 system, and analyses were performed using MSD Discovery Workbench software v.4.0. Calculated ECLIA parameters to measure binding antibody activities included interpolated concentrations or assigned arbitrary units (AU/ml) read from the standard curve.

Defining international units (IU)

Recently the arbitrary units were bridged to the WHO international standard and a conversion factor was calculated and confirmed. Parallelism between MSD reference standard and the WHO international standard was established for SARS-CoV proteins (S-2P, RBD, and N). Concentration assignments were performed and then confirmed by MSD and as part of a multi-site confirmation study. Sample results reported here have been converted to international units (IU/ml). S-specific IgG lower limit of detection = 0.3076 IU/ml. RBD-specific IgG lower limit of detection = 1.5936 IU/ml.

ELISA for temporal NHP serum antibodies and hamster serum antibodies

SARS-CoV-2 S-specific IgG in serum was quantified by ELISA; the methods used were similar to those previously published (*25*). Here, the only amendment to previously published methodology is the resulting data are depicted as endpoint titers, which were calculated as the mean serum titer that reached 10X SD using Prism v9.0.2 (GraphPad).

Meso-scale ELISA for mucosal antibody responses

Total S-specific IgG and IgA were determined by MULTI-ARRAY ELISA using Meso Scale technology (Meso Scale Discovery, MSD) as previously described (*42*).

Serum antibody avidity assay

Avidity was assessed using a sodium thiocyanate (NaSCN)-based avidity ELISA against the full-length SARS-CoV-2 S-2P antigen as previously described. The avidity index (AI) was calculated using the ratio of IgG binding to S-2P in the absence or presence of NaSCN. The reported AI is the average of two independent experiments, each containing duplicate samples.

ACE2-binding inhibition assay

ACE2-binding inhibition was completed, as previously described (*43*), on 1:40 diluted sera samples using Mesoscale Discovery 384-well, 4-Spot Custom Serology SECTOR® plates pre-coated with SARS-CoV-2 RBD. Binding was detected using SULFO-TAGTM labeled ACE2. Both reagents were generously supplied by the manufacturer.

Lentiviral pseudovirus neutralization assay

Pseudotyped lentiviral reporter viruses were produced by the co-transfection of plasmids encoding S proteins from Wuhan-1 strain (GenBank #: MN908947.3) with a D614G mutation, a luciferase reporter, lentivirus backbone, and human transmembrane protease serine 2 (TMPRSS2) genes into HEK293T/17 cells (ATCC CRL-11268) as previously described (*43*). Sera, in duplicate, were tested for neutralizing activity against the D614G pseudoviruses by quantification of luciferase activity in relative light units (RLU). Percent neutralization was normalized considering uninfected cells as 100% neutralization and cells infected with pseudovirus alone as

0% neutralization. ID50 titers were determined using a log(agonist) versus normalized-response (variable slope) nonlinear regression model in Prism v9.0.2 (GraphPad). The lower limit of quantification was 1:40 ID50.

VSV pseudovirus neutralization assay

To make SARS-CoV-2 pseudotyped recombinant VSV-ΔG-firefly luciferase virus, BHK21/WI-2 cells (Kerafast, EH1011) were transfected with the S plasmid expressing full-length S with D614G mutation and subsequently infected with VSVΔG-firefly-luciferase as previously described (*44*). Neutralization assays were completed on A549-ACE2-TMPRSS2 cells with serially diluted serum samples as previously described (*16*). The lower limit of quantification was 1:40 ID50.

Focus reduction neutralization test (FRNT)

VeroE6 cells (ATCC, #CRL-1586) were cultured, viral stocks [EHC-083E (D614G SARS-CoV-2) (*18*), B.1.1.7 (PMID 33739374), and B.1.351 (PMID 33972938)] were propagated and titered, and FRNT assays were performed as previously described (*45*). Antibody neutralization was quantified by counting the number of foci for each sample using the Viridot program (*46*). The neutralization titers were calculated as follows: 1 – [ratio of the mean number of foci in the presence of sera and foci at the highest dilution of respective sera sample]. Each specimen was tested in duplicate. The FRNT-50 titers were interpolated using a four-parameter nonlinear regression in GraphPad Prism v9.0.2.4.3. Samples that do not neutralize at the limit of detection at 50% are plotted at 5 and was used for geometric mean calculations.

Intracellular cytokine staining

Analysis of spike specific T cell immune responses from cryopreserved peripheral-blood mononuclear cells following vaccination was performed as previously described (*13*). The antibody clones, conjugates, commercial source, and analysis purpose for each reagent is shown in table S5. Data were collected on FACS A5 Analyzers (Becton Dickenson), and analyzed using FlowJo versions 9.9.6 and 10.7 (Becton Dickenson).

Correlations and statistical analysis

Graphs show data from individual animals with dotted lines indicating assay limits of detection. Correlations between antibody measurements, and between antibody measurements and dose of vaccine, are estimated and tested against 0 using Spearman's nonparametric correlation. Univariate and multivariate linear models are used to evaluate the potential role of antibodies as correlates of protection. Following the Prentice criteria (*47*), we categorized antibody measures as potential correlates of protection if they were significantly univariately predictive of log10 sgRNA as a measure of viral replication, and if the addition of log10 dose to the linear model did not significantly improve prediction, as assessed by a likelihood ratio test. Univariate and multivariate linear models were also used to explore the combination of T cell responses and antibody responses in prediction of sgRNA. Comparisons between pre-challenge and post-challenge were assessed using paired *t* tests, based on the last measurement (day 28 post-challenge when available, day 14 when not). Intracellular cytokines likely response labels were derived using the MIMOSA (*48*) package. Analyses were performed in R version 4.0.2 and Prism v9.0.2.

REFERENCES AND NOTES

1. E. Dong, H. Du, L. Gardner, An interactive web-based dashboard to track COVID-19 in real time. *Lancet Infect. Dis.* **20**, 533–534 (2020). doi:10.1016/S1473-3099(20)30120-1 Medline
2. J. Pallesen, N. Wang, K. S. Corbett, D. Wrapp, R. N. Kirchdoerfer, H. L. Turner, C. A. Cottrell, M. M. Becker, L. Wang, W. Shi, W.-P. Kong, E. L. Andres, A. N. Kettenbach, M. R. Denison, J. D. Chappell, B. S. Graham, A. B. Ward, J. S. McLellan, Immunogenicity and structures of a rationally designed prefusion MERS-CoV spike antigen. *Proc. Natl. Acad. Sci. U.S.A.* **114**, E7348–E7357 (2017). doi:10.1073/pnas.1707304114 Medline
3. D. Wrapp, N. Wang, K. S. Corbett, J. A. Goldsmith, C.-L. Hsieh, O. Abiona, B. S. Graham, J. S. McLellan, Cryo-EM structure of the 2019-nCoV spike in the prefusion conformation. *Science* **367**, 1260–1263 (2020). doi:10.1126/science.abb2507 Medline
4. F. P. Polack, S. J. Thomas, N. Kitchin, J. Absalon, A. Gurtman, S. Lockhart, J. L. Perez, G. Pérez Marc, E. D. Moreira, C. Zerbini, R. Bailey, K. A. Swanson, S. Roychoudhury, K. Koury, P. Li, W. V. Kalina, D. Cooper, R. W. Frenck Jr., L. L. Hammitt, Ö. Türeci, H. Nell, A. Schaefer, S. Ünal, D. B. Tresnan, S. Mather, P. R. Dormitzer, U. Şahin, K. U. Jansen, W. C. Gruber; C4591001 Clinical Trial Group, Safety and efficacy of the BNT162b2 mRNA Covid-19 vaccine. *N. Engl. J. Med.* **383**, 2603–2615 (2020). doi:10.1056/NEJMoa2034577 Medline
5. L. R. Baden, H. M. El Sahly, B. Essink, K. Kotloff, S. Frey, R. Novak, D. Diemert, S. A. Spector, N. Rouphael, C. B. Creech, J. McGettigan, S. Khetan, N. Segall, J. Solis, A. Brosz, C. Fierro, H. Schwartz, K. Neuzil, L. Corey, P. Gilbert, H. Janes, D. Follmann, M. Marovich, J. Mascola, L. Polakowski, J. Ledgerwood, B. S. Graham, H. Bennett, R. Pajon, C. Knightly, B. Leav, W. Deng, H. Zhou, S. Han, M. Ivarsson, J. Miller, T. Zaks; COVE Study Group, Efficacy and safety of the mRNA-1273 SARS-CoV-2 vaccine. *N. Engl. J. Med.* **384**, 403–416 (2021). doi:10.1056/NEJMoa2035389 Medline
6. M. Voysey, S. A. C. Clemens, S. A. Madhi, L. Y. Weckx, P. M. Folegatti, P. K. Aley, B. Angus, V. L. Baillie, S. L. Barnabas, Q. E. Bhorat, S. Bibi, C. Briner, P. Cicconi, A. M. Collins, R. Colin-Jones, C. L. Cutland, T. C. Darton, K. Dheda, C. J. A. Duncan, K. R. W. Emary, K. J. Ewer, L. Fairlie, S. N. Faust, S. Feng, D. M. Ferreira, A. Finn, A. L. Goodman, C. M. Green, C. A. Green, P. T. Heath, C. Hill, H. Hill, I. Hirsch, S. H. C. Hodgson, A. Izu, S. Jackson, D. Jenkin, C. C. D. Joe, S. Kerridge, A. Koen, G. Kwatra, R. Lazarus, A. M. Lawrie, A. Lelliott, V. Libri, P. J. Lillie, R. Mallory, A. V. A. Mendes, E. P. Milan, A. M. Minassian, A. McGregor, H. Morrison, Y. F. Mujadidi, A. Nana, P. J. O'Reilly, S. D. Padayachee, A. Pittella, E. Plested, K. M. Pollock, M. N. Ramasamy, S. Rhead, A. V. Schwarzbold, N. Singh, A. Smith, R. Song, M. D. Snape, E. Sprinz, R. K. Sutherland, R. Tarrant, E. C. Thomson, M. E. Török, M. Toshner, D. P. J. Turner, J. Vekemans, T. L. Villafana, M. E. E. Watson, C. J. Williams, A. D. Douglas, A. V. S. Hill, T. Lambe, S. C. Gilbert, A. J. Pollard, Oxford COVID Vaccine Trial Group, Safety and efficacy of the ChAdOx1 nCoV-19 vaccine (AZD1222) against SARS-CoV-2: An interim analysis of four randomised controlled trials in Brazil, South Africa, and the UK. *Lancet* **397**, 99–111 (2021). doi:10.1016/S0140-6736(20)32661-1 Medline
7. D. Y. Logunov, I. V. Dolzhikova, D. V. Shcheblyakov, A. I. Tukhvatulin, O. V. Zubkova, A. S. Dzharullaeva, A. V. Kovyrshina, N. L. Lubenets, D. M. Grousova, A. S. Erokhova, A. G. Botikov, F. M. Izhaeva, O. Popova, T. A. Ozharovskaya, I. B. Esmagambetov, I. A. Favorskaya, D. I. Zrelkin, D. V. Voronina, D. N. Shcherbinin, A. S. Semikhin, Y. V. Simakova, E. A. Tokarskaya, D. A. Egorova, M. M. Shmarov,

N. A. Nikitenko, V. A. Gushchin, E. A. Smolyarchuk, S. K. Zyryanov, S. V. Borisevich, B. S. Naroditsky, A. L. Gintsburg; Gam-COVID-Vac Vaccine Trial Group, Safety and efficacy of an rAd26 and rAd5 vector-based heterologous prime-boost COVID-19 vaccine: An interim analysis of a randomised controlled phase 3 trial in Russia. *Lancet* **397**, 671–681 (2021). doi:10.1016/S0140-6736(21)00234-8 Medline
8. G. Forni, A. Mantovani; COVID-19 Commission of Accademia Nazionale dei Lincei, Rome, COVID-19 vaccines: Where we stand and challenges ahead. *Cell Death Differ.* **28**, 626–639 (2021). doi:10.1038/s41418-020-00720-9 Medline
9. S. A. Plotkin, P. B. Gilbert, Nomenclature for immune correlates of protection after vaccination. *Clin. Infect. Dis.* **54**, 1615–1617 (2012). doi:10.1093/cid/cis238 Medline
10. P. J. Klasse, D. F. Nixon, J. P. Moore, Immunogenicity of clinically relevant SARS-CoV-2 vaccines in nonhuman primates and humans. *Sci. Adv.* **7**, eabe8065 (2021). doi:10.1126/sciadv.abe8065 Medline
11. J. Yu, L. H. Tostanoski, L. Peter, N. B. Mercado, K. McMahan, S. H. Mahrokhian, J. P. Nkolola, J. Liu, Z. Li, A. Chandrashekar, D. R. Martinez, C. Loos, C. Atyeo, S. Fischinger, J. S. Burke, M. D. Slein, Y. Chen, A. Zuiani, F. J. N. Lelis, M. Travers, S. Habibi, L. Pessaint, A. Van Ry, K. Blade, R. Brown, A. Cook, B. Finneyfrock, A. Dodson, E. Teow, J. Velasco, R. Zahn, F. Wegmann, E. A. Bondzie, G. Dagotto, M. S. Gebre, X. He, C. Jacob-Dolan, M. Kirilova, N. Kordana, Z. Lin, L. F. Maxfield, F. Nampanya, R. Nityanandam, J. D. Ventura, H. Wan, Y. Cai, B. Chen, A. G. Schmidt, D. R. Wesemann, R. S. Baric, G. Alter, H. Andersen, M. G. Lewis, D. H. Barouch, DNA vaccine protection against SARS-CoV-2 in rhesus macaques. *Science* **369**, 806–811 (2020). doi:10.1126/science.abc6284 Medline
12. N. B. Mercado, R. Zahn, F. Wegmann, C. Loos, A. Chandrashekar, J. Yu, J. Liu, L. Peter, K. McMahan, L. H. Tostanoski, X. He, D. R. Martinez, L. Rutten, R. Bos, D. van Manen, J. Vellinga, J. Custers, J. P. Langedijk, T. Kwaks, M. J. G. Bakkers, D. Zuijdgeest, S. K. Rosendahl Huber, C. Atyeo, S. Fischinger, J. S. Burke, J. Feldman, B. M. Hauser, T. M. Caradonna, E. A. Bondzie, G. Dagotto, M. S. Gebre, E. Hoffman, C. Jacob-Dolan, M. Kirilova, Z. Li, Z. Lin, S. H. Mahrokhian, L. F. Maxfield, F. Nampanya, R. Nityanandam, J. P. Nkolola, S. Patel, J. D. Ventura, K. Verrington, H. Wan, L. Pessaint, A. Van Ry, K. Blade, A. Strasbaugh, M. Cabus, R. Brown, A. Cook, S. Zouantchangadou, E. Teow, H. Andersen, M. G. Lewis, Y. Cai, B. Chen, A. G. Schmidt, R. K. Reeves, R. S. Baric, D. A. Lauffenburger, G. Alter, P. Stoffels, M. Mammen, J. Van Hoof, H. Schuitemaker, D. H. Barouch, Single-shot Ad26 vaccine protects against SARS-CoV-2 in rhesus macaques. *Nature* **586**, 583–588 (2020). doi:10.1038/s41586-020-2607-z Medline
13. K. S. Corbett, B. Flynn, K. E. Foulds, J. R. Francica, S. Boyoglu-Barnum, A. P. Werner, B. Flach, S. O'Connell, K. W. Bock, M. Minai, B. M. Nagata, H. Andersen, D. R. Martinez, A. T. Noe, N. Douek, M. M. Donaldson, N. N. Nji, G. S. Alvarado, D. K. Edwards, D. R. Flebbe, E. Lamb, N. A. Doria-Rose, B. C. Lin, M. K. Louder, S. O'Dell, S. D. Schmidt, E. Phung, L. A. Chang, C. Yap, J. M. Todd, L. Pessaint, A. Van Ry, S. Browne, J. Greenhouse, T. Putman-Taylor, A. Strasbaugh, T.-A. Campbell, A. Cook, A. Dodson, K. Steingrebe, W. Shi, Y. Zhang, O. M. Abiona, L. Wang, A. Pegu, E. S. Yang, K. Leung, T. Zhou, I.-T. Teng, A. Widge, I. Gordon, L. Novik, R. A. Gillespie, R. J. Loomis, J. I. Moliva, G. Stewart-Jones, S. Himansu, W.-P. Kong, M. C. Nason, K. M. Morabito, T. J. Ruckwardt, J. E. Ledgerwood, M. R. Gaudinski, P. D. Kwong, J. R. Mascola, A. Carfi, M. G. Lewis, R. S. Baric, A. McDermott, I. N. Moore, N. J. Sullivan, M. Roederer, R. A. Seder, B. S. Graham, Evaluation of the mRNA-1273 Vaccine against SARS-CoV-2 in Nonhuman Primates. *N. Engl. J. Med.* **383**, 1544–1555 (2020). doi:10.1056/NEJMoa2024671 Medline
14. V. J. Munster, F. Feldmann, B. N. Williamson, N. van Doremalen, L. Pérez-Pérez, J. Schulz, K. Meade-White, A. Okumura, J. Callison, B. Brumbaugh, V. A. Avanzato, R. Rosenke, P. W. Hanley, G. Saturday, D. Scott, E. R. Fischer, E. de Wit, Respiratory disease in rhesus macaques inoculated with SARS-CoV-2. *Nature* **585**, 268–272 (2020). doi:10.1038/s41586-020-2324-7 Medline
15. K. McMahan, J. Yu, N. B. Mercado, C. Loos, L. H. Tostanoski, A. Chandrashekar, J. Liu, L. Peter, C. Atyeo, A. Zhu, E. A. Bondzie, G. Dagotto, M. S. Gebre, C. Jacob-Dolan, Z. Li, F. Nampanya, S. Patel, L. Pessaint, A. Van Ry, K. Blade, J. Yalley-Ogunro, M. Cabus, R. Brown, A. Cook, E. Teow, H. Andersen, M. G. Lewis, D. A. Lauffenburger, G. Alter, D. H. Barouch, Correlates of protection against SARS-CoV-2 in rhesus macaques. *Nature* **590**, 630–634 (2021). doi:10.1038/s41586-020-03041-6 Medline
16. K. Wu, A. P. Werner, M. Koch, A. Choi, E. Narayanan, G. B. E. Stewart-Jones, T. Colpitts, H. Bennett, S. Boyoglu-Barnum, W. Shi, J. I. Moliva, N. J. Sullivan, B. S. Graham, A. Carfi, K. S. Corbett, R. A. Seder, D. K. Edwards, Serum neutralizing activity elicited by mRNA-1273 vaccine. *N. Engl. J. Med.* **384**, 1468–1470 (2021). doi:10.1056/NEJMc2102179 Medline
17. X. Shen, H. Tang, R. Pajon, G. Smith, G. M. Glenn, W. Shi, B. Korber, D. C. Montefiori, Neutralization of SARS-CoV-2 variants B.1.429 and B.1.351. *N. Engl. J. Med.* **384**, 2352–2354 (2021). doi:10.1056/NEJMc2103740 Medline
18. V. V. Edara, C. Norwood, K. Floyd, L. Lai, M. E. Davis-Gardner, W. H. Hudson, G. Mantus, L. E. Nyhoff, M. W. Adelman, R. Fineman, S. Patel, R. Byram, D. N. Gomes, G. Michael, H. Abdullahi, N. Beydoun, B. Panganiban, N. McNair, K. Hellmeister, J. Pitts, J. Winters, J. Kleinhenz, J. Usher, J. B. O'Keefe, A. Piantadosi, J. J. Waggoner, A. Babiker, D. S. Stephens, E. J. Anderson, S. Edupuganti, N. Rouphael, R. Ahmed, J. Wrammert, M. S. Suthar, Infection- and vaccine-induced antibody binding and neutralization of the B.1.351 SARS-CoV-2 variant. *Cell Host Microbe* **29**, 516–521.e3 (2021). doi:10.1016/j.chom.2021.03.009 Medline
19. B. Huang, L. Dai, H. Wang, Z. Hu, X. Yang, W. Tan, G. F. Gao, Serum sample neutralisation of BBIBP-CorV and ZF2001 vaccines to SARS-CoV-2 501Y.V2. *Lancet Microbe* **2**, e285 (2021). doi:10.1016/S2666-5247(21)00082-3 Medline
20. Y. Liu, J. Liu, H. Xia, X. Zhang, C. R. Fontes-Garfias, K. A. Swanson, H. Cai, R. Sarkar, W. Chen, M. Cutler, D. Cooper, S. C. Weaver, A. Muik, U. Sahin, K. U. Jansen, X. Xie, P. R. Dormitzer, P.-Y. Shi, Neutralizing activity of BNT162b2-elicited serum. *N. Engl. J. Med.* **384**, 1466–1468 (2021). doi:10.1056/NEJMc2102017 Medline
21. D. Frampton, T. Rampling, A. Cross, H. Bailey, J. Heaney, M. Byott, R. Scott, R. Sconza, J. Price, M. Margaritis, M. Bergstrom, M. J. Spyer, P. B. Miralhes, P. Grant, S. Kirk, C. Valerio, Z. Mangera, T. Prabhahar, J. Moreno-Cuesta, N. Arulkumaran, M. Singer, G. Y. Shin, E. Sanchez, S. M. Paraskevopoulou, D. Pillay, R. A. McKendry, M. Mirfenderesky, C. F. Houlihan, E. Nastouli, Genomic characteristics and clinical effect of the emergent SARS-CoV-2 B.1.1.7 lineage in London, UK: A whole-genome sequencing and hospital-based cohort study. *Lancet Infect. Dis.* S1473-3099(21)00170-5 (2021). doi:10.1016/S1473-3099(21)00170-5 Medline
22. N. G. Davies, S. Abbott, R. C. Barnard, C. I. Jarvis, A. J. Kucharski, J. D. Munday, C. A. B. Pearson, T. W. Russell, D. C. Tully, A. D. Washburne, T. Wenseleers, A. Gimma, W. Waites, K. L. M. Wong, K. van Zandvoort, J. D. Silverman, K. Diaz-Ordaz, R. Keogh, R. M. Eggo, S. Funk, M. Jit, K. E. Atkins, W. J. Edmunds; CMMID COVID-19 Working Group; COVID-19 Genomics UK (COG-UK) Consortium, Estimated transmissibility and impact of SARS-CoV-2 lineage B.1.1.7 in England. *Science* **372**, eabg3055 (2021). doi:10.1126/science.abg3055 Medline
23. H. Tegally, E. Wilkinson, M. Giovanetti, A. Iranzadeh, V. Fonseca, J. Giandhari, D. Doolabh, S. Pillay, E. J. San, N. Msomi, K. Mlisana, A. von Gottberg, S. Walaza, M. Allam, A. Ismail, T. Mohale, A. J. Glass, S. Engelbrecht, G. Van Zyl, W. Preiser, F. Petruccione, A. Sigal, D. Hardie, G. Marais, M. Hsiao, S. Korsman, M.-A. Davies, L. Tyers, I. Mudau, D. York, C. Maslo, D. Goedhals, S. Abrahams, O. Laguda-Akingba, A. Alisoltani-Dehkordi, A. Godzik, C. K. Wibmer, B. T. Sewell, J. Lourenço, L. C. J. Alcantara, S. L. Kosakovsky Pond, S. Weaver, D. Martin, R. J. Lessells, J. N. Bhiman, C. Williamson, T. de Oliveira, Emergence and rapid spread of a new severe acute respiratory syndrome-related coronavirus 2 (SARS-CoV-2) lineage with multiple spike mutations in South Africa. medRxiv 2020.2012.2021.20248640 [Preprint]. 22 December 2020. https://doi.org/10.1101/2020.12.21.20248640.
24. D. Planas, T. Bruel, L. Grzelak, F. Guivel-Benhassine, I. Staropoli, F. Porrot, C. Planchais, J. Buchrieser, M. M. Rajah, E. Bishop, M. Albert, F. Donati, M. Prot, S. Behillil, V. Enouf, M. Maquart, M. Smati-Lafarge, E. Varon, F. Schortgen, L. Yahyaoui, M. Gonzalez, J. De Sèze, H. Péré, D. Veyer, A. Sève, E. Simon-Lorière, S. Fafi-Kremer, K. Stefic, H. Mouquet, L. Hocqueloux, S. van der Werf, T. Prazuck, O. Schwartz, Sensitivity of infectious SARS-CoV-2 B.1.1.7 and B.1.351 variants to neutralizing antibodies. *Nat. Med.* **27**, 917–924 (2021). doi:10.1038/s41591-021-01318-5 Medline
25. K. S. Corbett, D. Edwards, S. R. Leist, O. M. Abiona, S. Boyoglu-Barnum, R. A. Gillespie, S. Himansu, A. Schäfer, C. T. Ziwawo, A. T. DiPiazza, K. H. Dinnon, S. M. Elbashir, C. A. Shaw, A. Woods, E. J. Fritch, D. R. Martinez, K. W. Bock, M. Minai, B. M. Nagata, G. B. Hutchinson, K. Bahl, D. Garcia-Dominguez, L. Ma, I. Renzi, W. P. Kong, S. D. Schmidt, L. Wang, Y. Zhang, L. J. Stevens, E. Phung, L. A. Chang, R. J. Loomis, N. E. Altaras, E. Narayanan, M. Metkar, V. Presnyak, C. Liu, M. K. Louder, W. Shi, K. Leung, E. S. Yang, A. West, K. L. Gully, N. Wang, D. Wrapp, N. A. Doria-Rose, G. Stewart-Jones, H. Bennett, M. C. Nason, T. J. Ruckwardt, J. S. McLellan, M. R. Denison, J. D. Chappell, I. N. Moore, K. M. Morabito, J. R. Mascola, R. S. Baric, A. Carfi, B. S. Graham, SARS-CoV-2 mRNA vaccine development enabled by

prototype pathogen preparedness. *bioRxiv* 2020.06.11.145920 (2020). Medline
26. N. Pardi, M. J. Hogan, M. S. Naradikian, K. Parkhouse, D. W. Cain, L. Jones, M. A. Moody, H. P. Verkerke, A. Myles, E. Willis, C. C. LaBranche, D. C. Montefiori, J. L. Lobby, K. O. Saunders, H.-X. Liao, B. T. Korber, L. L. Sutherland, R. M. Scearce, P. T. Hraber, I. Tombácz, H. Muramatsu, H. Ni, D. A. Balikov, C. Li, B. L. Mui, Y. K. Tam, F. Krammer, K. Karikó, P. Polacino, L. C. Eisenlohr, T. D. Madden, M. J. Hope, M. G. Lewis, K. K. Lee, S.-L. Hu, S. E. Hensley, M. P. Cancro, B. F. Haynes, D. Weissman, Nucleoside-modified mRNA vaccines induce potent T follicular helper and germinal center B cell responses. *J. Exp. Med.* **215**, 1571–1588 (2018). doi:10.1084/jem.20171450 Medline
27. L. Zou, F. Ruan, M. Huang, L. Liang, H. Huang, Z. Hong, J. Yu, M. Kang, Y. Song, J. Xia, Q. Guo, T. Song, J. He, H.-L. Yen, M. Peiris, J. Wu, SARS-CoV-2 viral load in upper respiratory specimens of infected patients. *N. Engl. J. Med.* **382**, 1177–1179 (2020). doi:10.1056/NEJMc2001737 Medline
28. D. Kim, J.-Y. Lee, J.-S. Yang, J. W. Kim, V. N. Kim, H. Chang, The architecture of SARS-CoV-2 transcriptome. *Cell* **181**, 914–921.e10 (2020). doi:10.1016/j.cell.2020.04.011 Medline
29. V. J. Munster, F. Feldmann, B. N. Williamson, N. van Doremalen, L. Pérez-Pérez, J. Schulz, K. Meade-White, A. Okumura, J. Callison, B. Brumbaugh, V. A. Avanzato, R. Rosenke, P. W. Hanley, G. Saturday, D. Scott, E. R. Fischer, E. de Wit, Respiratory disease in rhesus macaques inoculated with SARS-CoV-2. *Nature* **585**, 268–272 (2020). doi:10.1038/s41586-020-2324-7 Medline
30. A. Chandrashekar, J. Liu, A. J. Martinot, K. McMahan, N. B. Mercado, L. Peter, L. H. Tostanoski, J. Yu, Z. Maliga, M. Nekorchuk, K. Busman-Sahay, M. Terry, L. M. Wrijil, S. Ducat, D. R. Martinez, C. Atyeo, S. Fischinger, J. S. Burke, M. D. Slein, L. Pessaint, A. Van Ry, J. Greenhouse, T. Taylor, K. Blade, A. Cook, B. Finneyfrock, R. Brown, E. Teow, J. Velasco, R. Zahn, F. Wegmann, P. Abbink, E. A. Bondzie, G. Dagotto, M. S. Gebre, X. He, C. Jacob-Dolan, N. Kordana, Z. Li, M. A. Lifton, S. H. Mahrokhian, L. F. Maxfield, R. Nityanandam, J. P. Nkolola, A. G. Schmidt, A. D. Miller, R. S. Baric, G. Alter, P. K. Sorger, J. D. Estes, H. Andersen, M. G. Lewis, D. H. Barouch, SARS-CoV-2 infection protects against rechallenge in rhesus macaques. *Science* **369**, 812–817 (2020). doi:10.1126/science.abc4776 Medline
31. B. Rockx, T. Kuiken, S. Herfst, T. Bestebroer, M. M. Lamers, B. B. Oude Munnink, D. de Meulder, G. van Amerongen, J. van den Brand, N. M. A. Okba, D. Schipper, P. van Run, L. Leijten, R. Sikkema, E. Verschoor, B. Verstrepen, W. Bogers, J. Langermans, C. Drosten, M. Fentener van Vlissingen, R. Fouchier, R. de Swart, M. Koopmans, B. L. Haagmans, Comparative pathogenesis of COVID-19, MERS, and SARS in a nonhuman primate model. *Science* **368**, 1012–1015 (2020). doi:10.1126/science.abb7314 Medline
32. M. Imai, K. Iwatsuki-Horimoto, M. Hatta, S. Loeber, P. J. Halfmann, N. Nakajima, T. Watanabe, M. Ujie, K. Takahashi, M. Ito, S. Yamada, S. Fan, S. Chiba, M. Kuroda, L. Guan, K. Takada, T. Armbrust, A. Balogh, Y. Furusawa, M. Okuda, H. Ueki, A. Yasuhara, Y. Sakai-Tagawa, T. J. S. Lopes, M. Kiso, S. Yamayoshi, N. Kinoshita, N. Ohmagari, S. I. Hattori, M. Takeda, H. Mitsuya, F. Krammer, T. Suzuki, Y. Kawaoka, Syrian hamsters as a small animal model for SARS-CoV-2 infection and countermeasure development. *Proc. Natl. Acad. Sci. U.S.A.* **117**, 16587–16595 (2020). doi:10.1073/pnas.2009799117 Medline
33. R. Wölfel, V. M. Corman, W. Guggemos, M. Seilmaier, S. Zange, M. A. Müller, D. Niemeyer, T. C. Jones, P. Vollmar, C. Rothe, M. Hoelscher, T. Bleicker, S. Brünink, J. Schneider, R. Ehmann, K. Zwirglmaier, C. Drosten, C. Wendtner, Virological assessment of hospitalized patients with COVID-2019. *Nature* **581**, 465–469 (2020). doi:10.1038/s41586-020-2196-x Medline
34. D. Shan, J. M. Johnson, S. C. Fernandes, H. Suib, S. Hwang, D. Wuelfing, M. Mendes, M. Holdridge, E. M. Burke, K. Beauregard, Y. Zhang, M. Cleary, S. Xu, X. Yao, P. P. Patel, T. Plavina, D. H. Wilson, L. Chang, K. M. Kaiser, J. Nattermann, S. V. Schmidt, E. Latz, K. Hrusovsky, D. Mattoon, A. J. Ball, N-protein presents early in blood, dried blood and saliva during asymptomatic and symptomatic SARS-CoV-2 infection. *Nat. Commun.* **12**, 1931 (2021). doi:10.1038/s41467-021-22072-9 Medline
35. J. Fajnzylber, J. Regan, K. Coxen, H. Corry, C. Wong, A. Rosenthal, D. Worrall, F. Giguel, A. Piechocka-Trocha, C. Atyeo, S. Fischinger, A. Chan, K. T. Flaherty, K. Hall, M. Dougan, E. T. Ryan, E. Gillespie, R. Chishti, Y. Li, N. Jilg, D. Hanidziar, R. M. Baron, L. Baden, A. M. Tsibris, K. A. Armstrong, D. R. Kuritzkes, G. Alter, B. D. Walker, X. Yu, J. Z. Li; Massachusetts Consortium for Pathogen Readiness, SARS-CoV-2 viral load is associated with increased disease severity and mortality. *Nat. Commun.* **11**, 5493 (2020). doi:10.1038/s41467-020-19057-5 Medline
36. D. E. Dimcheff, A. L. Valesano, K. E. Rumfelt, W. J. Fitzsimmons, C. Blair, C. Mirabelli, J. G. Petrie, E. T. Martin, C. Bhambhani, M. Tewari, A. S. Lauring, SARS-CoV-2 total and subgenomic RNA viral load in hospitalized patients. medRxiv 2021.02.25.21252493 [Preprint]. 1 March 2021. https://doi.org/10.1101/2021.02.25.21252493.
37. S. C. Adenyi-Jones, H. Faden, M. B. Ferdon, M. S. Kwong, P. L. Ogra, Systemic and local immune responses to enhanced-potency inactivated poliovirus vaccine in premature and term infants. *J. Pediatr.* **120**, 686–689 (1992). doi:10.1016/S0022-3476(05)80228-8 Medline
38. P. L. Ogra, Mucosal immune response to poliovirus vaccines in childhood. *Rev. Infect. Dis.* **6** (Suppl 2), S361–S368 (1984). doi:10.1093/clinids/6.Supplement_2.S361 Medline
39. P. L. Ogra, D. T. Karzon, F. Righthand, M. MacGillivray, Immunoglobulin response in serum and secretions after immunization with live and inactivated poliovaccine and natural infection. *N. Engl. J. Med.* **279**, 893–900 (1968). doi:10.1056/NEJM196810242791701 Medline
40. I. M. Onorato, J. F. Modlin, A. M. McBean, M. L. Thoms, G. A. Losonsky, R. H. Bernier, Mucosal immunity induced by enhance-potency inactivated and oral polio vaccines. *J. Infect. Dis.* **163**, 1–6 (1991). doi:10.1093/infdis/163.1.1 Medline
41. G. Zhaori, M. Sun, P. L. Ogra, Characteristics of the immune response to poliovirus virion polypeptides after immunization with live or inactivated polio vaccines. *J. Infect. Dis.* **158**, 160–165 (1988). doi:10.1093/infdis/158.1.160 Medline
42. J. R. Francica, B. J. Flynn, K. E. Foulds, A. T. Noe, A. P. Werner, I. N. Moore, M. Gagne, T. S. Johnston, C. Tucker, R. L. Davis, B. Flach, S. O'Connell, S. F. Andrew, E. Lamb, D. R. Flebbe, S. T. Nurmukhambetova, M. M. Donaldson, J.-P. M. Todd, A. L. Zhu, C. Atyeo, S. Fischinger, M. J. Gorman, S. Shin, V. Viswanadh Edara, K. Floyd, L. Lai, A. Tylor, E. McCarthy, V. Lecouturier, S. Ruiz, C. Berry, T. Tibbitts, H. Andersen, A. Cook, A. Dodson, L. Pessaint, A. Van Ry, M. Koutsoukos, C. Gutzeit, I.-T. Teng, T. Zhou, D. Li, B. F. Haynes, P. D. Kwong, A. McDermott, M. G. Lewis, T. M. Fu, R. Chicz, R. van der Most, K. S. Corbett, M. S. Suthar, G. Alter, M. Roederer, N. J. Sullivan, D. C. Douek, B. S. Graham, D. Casimiro, R. A. Seder, Vaccination with SARS-CoV-2 spike protein and AS03 adjuvant induces rapid anamnestic antibodies in the lung and protects against virus challenge in nonhuman primates. bioRxiv 433390 [Preprint]. 2 March 2021. https://doi.org/10.1101/2021.03.02.433390.
43. L. A. Jackson, E. J. Anderson, N. G. Rouphael, P. C. Roberts, M. Makhene, R. N. Coler, M. P. McCullough, J. D. Chappell, M. R. Denison, L. J. Stevens, A. J. Pruijssers, A. McDermott, B. Flach, N. A. Doria-Rose, K. S. Corbett, K. M. Morabito, S. O'Dell, S. D. Schmidt, P. A. Swanson 2nd, M. Padilla, J. R. Mascola, K. M. Neuzil, H. Bennett, W. Sun, E. Peters, M. Makowski, J. Albert, K. Cross, W. Buchanan, R. Pikaart-Tautges, J. E. Ledgerwood, B. S. Graham, J. H. Beigel; mRNA-1273 Study Group, An mRNA vaccine against SARS-CoV-2: Preliminary report. *N. Engl. J. Med.* **383**, 1920–1931 (2020). doi:10.1056/NEJMoa2022483 Medline
44. M. A. Whitt, Generation of VSV pseudotypes using recombinant ΔG-VSV for studies on virus entry, identification of entry inhibitors, and immune responses to vaccines. *J. Virol. Methods* **169**, 365–374 (2010). doi:10.1016/j.jviromet.2010.08.006 Medline
45. A. Vanderheiden, V. V. Edara, K. Floyd, R. C. Kauffman, G. Mantus, E. Anderson, N. Rouphael, S. Edupuganti, P.-Y. Shi, V. D. Menachery, J. Wrammert, M. S. Suthar, Development of a rapid focus reduction neutralization test assay for measuring SARS-CoV-2 neutralizing antibodies. *Curr. Protoc. Immunol.* **131**, e116 (2020). doi:10.1002/cpim.116 Medline
46. L. C. Katzelnick, A. Coello Escoto, B. D. McElvany, C. Chávez, H. Salje, W. Luo, I. Rodriguez-Barraquer, R. Jarman, A. P. Durbin, S. A. Diehl, D. J. Smith, S. S. Whitehead, D. A. T. Cummings, Viridot: An automated virus plaque (immunofocus) counter for the measurement of serological neutralizing responses with application to dengue virus. *PLOS Negl. Trop. Dis.* **12**, e0006862 (2018). doi:10.1371/journal.pntd.0006862 Medline
47. R. L. Prentice, Surrogate endpoints in clinical trials: Definition and operational criteria. *Stat. Med.* **8**, 431–440 (1989). doi:10.1002/sim.4780080407 Medline
48. G. Finak, A. McDavid, P. Chattopadhyay, M. Dominguez, S. De Rosa, M. Roederer, R. Gottardo, Mixture models for single-cell assays with applications to vaccine studies. *Biostatistics* **15**, 87–101 (2014). doi:10.1093/biostatistics/kxt024 Medline

ACKNOWLEDGMENTS

We thank M. Sriparna for key editing following review of the manuscript. We thank T. Ruckwardt, N. Doria-Rose, and additional members of all included laboratories for critical discussions and advice pertaining to experiments included in the manuscript. We thank J. Stein and M. Young for technology transfer and administrative support, respectively. We thank members of the NIH NIAID VRC Translational Research Program, including C. Case, H. Bao, E. McCarthy, J. Noor, A. Taylor, and R. Woodward, for technical and administrative assistance with animal experiments. We thank H. Mu and M Farzan for the ACE2-overexpressing 293 cells. We thank the laboratory of P. Kwong for providing protein for use in ELISA assays for detection of mucosal antibodies. We thank A. Pekosz for the B.1.351 variant used in FRNT assays and E Boritz for assistance with B.1.351 sequencing and analysis. We thank M. Brunner and Dr. M. Whitt for kind support on recombinant VSV-based SARS-CoV-2 pseudovirus production. **Funding:** Intramural Research Program of the VRC, NIAID, NIH; Department of Health and Human Services, Office of the Assistant Secretary for Preparedness and Response, Biomedical Advanced Research and Development Authority, Contract 75A50120C00034; Undergraduate Scholarship Program, Office of Intramural Training and Education, Office of the Director, NIH (K.S.C.); NIAID Research Participation Program, administered by the Oak Ridge Institute for Science and Education through an interagency agreement between the U.S. Department of Energy and NIAID (R.W.); Emory Executive Vice President for Health Affairs Synergy Fund Award (M.S.S.); Pediatric Research Alliance Center for Childhood Infections and Vaccines and Children's Healthcare of Atlanta (M.S.S.); Woodruff Health Sciences Center 2020 COVID-19 CURE Award (M.S.S.). **Author contributions:** K.S.C., M.C.N, B.F., M.G., S.O., T.S.J., S.N.S., V.V.E., K.F., L.L., C.M., J.F., B.F., K.W., A.C., M.K., A.P.W., J.I.M., O.M.A., S.F.A., M.M.D., J.F., D.R.F., E.L., A.T.N., S.T.N., S.J.P., A.C., A.D., A.F., J.G., S.K., L.P., M.P., K.S., D.V., S.Z., K.W.B., M.M., B.M.N., R.V., H.A., K.E.F., D.K.E., J.R.M., I.N.M., M.G.L., A.C., D.M., M.S.S, A.M., N.J.S., M.R., D.C.D., B.S.G., and R.A.S. designed, completed, and/or analyzed experiments. O.M.A., S.B-B., K.L., W.S., E.S.Y., Y.Z., and L.W. provided critical published reagents/analytic tools. K.S.C., M.C.N., N.J.S., M.R., B.S.G, and R.A.S. wrote the manuscript. K.S.C., M.C.N., M.G., and G.A. prepared figures and tables. All authors contributed to discussions about and editing of the manuscript. **Competing interests:** K.S.C. and B.S.G. are inventors on U.S. Patent No. 10,960,070 B2 and International Patent Application No. WO/2018/081318 entitled "Prefusion Coronavirus Spike Proteins and Their Use." K.S.C., O.M.A., and B.S.G. are inventors on US Patent Application No. 62/972,886 entitled "2019-nCoV Vaccine". M.S.S. is on the Advisory Board of Moderna. D.V. holds equity in Johnson and Johnson and ABBVIE. **Data and materials availability:** All data are available in the main text or the supplementary materials. mRNA1273 was provided under an RCA with Moderna. To discuss access to this reagent, researchers should contact Andrea Carfi or Darin Edwards at Moderna. This work is licensed under a Creative Commons Attribution 4.0 International (CC BY 4.0) license, which permits unrestricted use, distribution, and reproduction in any medium, provided the original work is properly cited. To view a copy of this license, visit https://creativecommons.org/licenses/by/4.0/. This license does not apply to figures/photos/artwork or other content included in the article that is credited to a third party; obtain authorization from the rights holder before using such material.

SUPPLEMENTARY MATERIALS

science.sciencemag.org/cgi/content/full/science.abj0299/DC1
Figs. S1 to S11
Tables S1 to S5
MDAR Reproducibility Checklist

20 April 2021; accepted 27 July 2021
Published online 29 July 2021
10.1126/science.abj0299

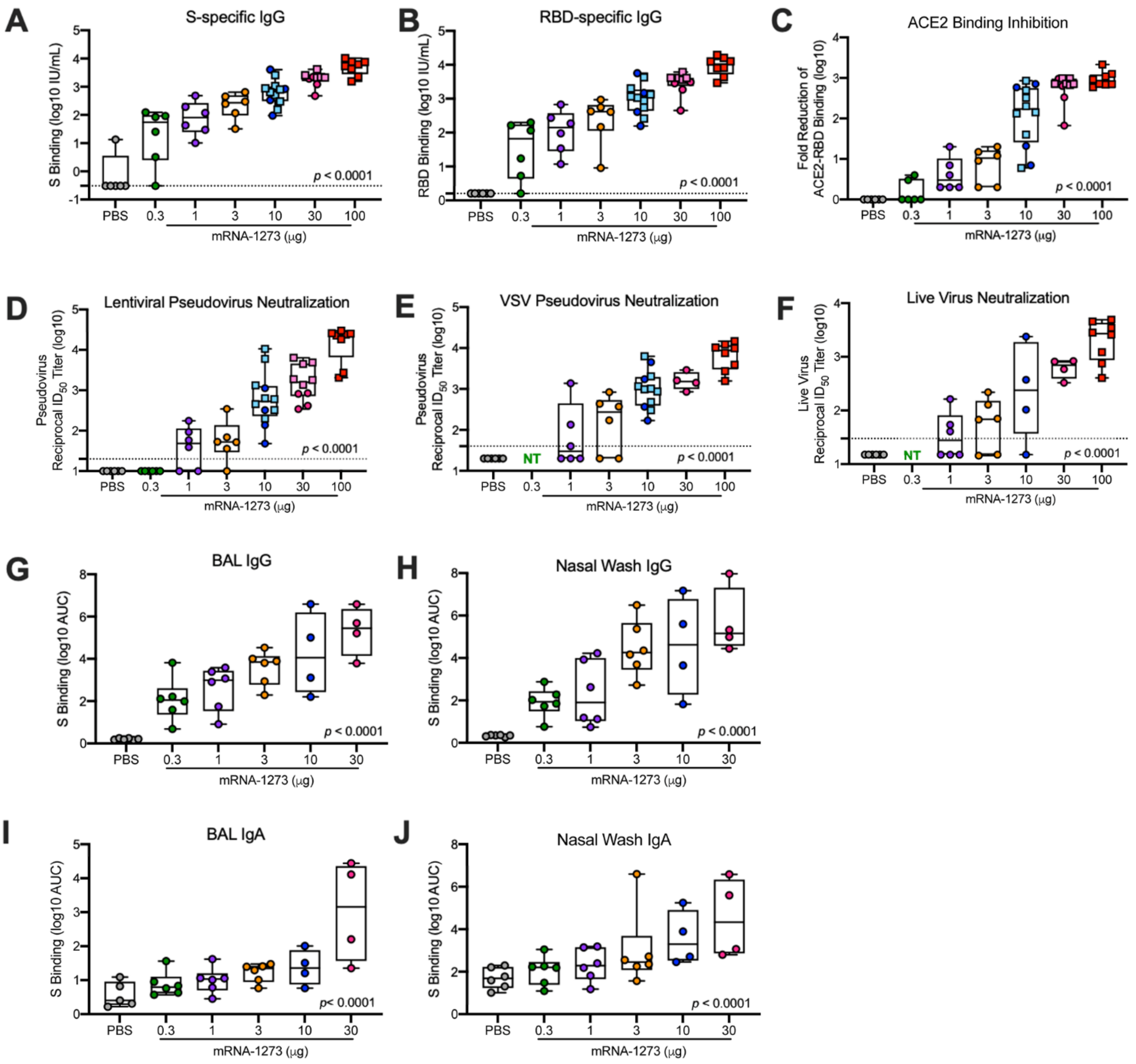

Fig. 1. Antibody responses following mRNA-1273 immunization. (**A** to **F**) Rhesus macaques were immunized according to fig. S1 with PBS (gray) or mRNA-1273 (0.3 μg – green, 1 μg – purple, 3 μg – orange, 10 μg – blue, 30 μg – pink, or 100 μg – red). Sera collected 4 weeks post-boost, immediately before challenge, were assessed for SARS-CoV-2 S-specific (A) and RBD-specific (B) IgG by MULTI-ARRAY ELISA, inhibition of ACE2 binding to RBD (C), SARS-CoV-2 lentiviral-based pseudovirus neutralization (D), SARS-CoV-2 VSV-based pseudovirus neutralization (E), and SARS-CoV-2 EHC-83E focus reduction neutralization (F). (**G** to **J**) BAL (G and I) and nasal washes (H and J) collected 2 weeks post-boost were assessed for SARS-CoV-2 S-specific IgG (G to H) and IgA (I to J) by MULTI-ARRAY ELISA. Squares represent NHPs in previous experiments (S1A, VRC-20-857.1 and S1B, VRC-20-857.2) and circles represent individual NHPs in experiment S1C, VRC-20-857.4. Boxes and horizontal bars denote the IQR and medians, respectively. Whisker endpoints are equal to the maximum and minimum values. Dotted lines indicate assay limits of detection, where applicable. NT: not tested. All measures were significantly correlated with dose ($P<0.0001$), as determined by a test of Spearman's correlation.

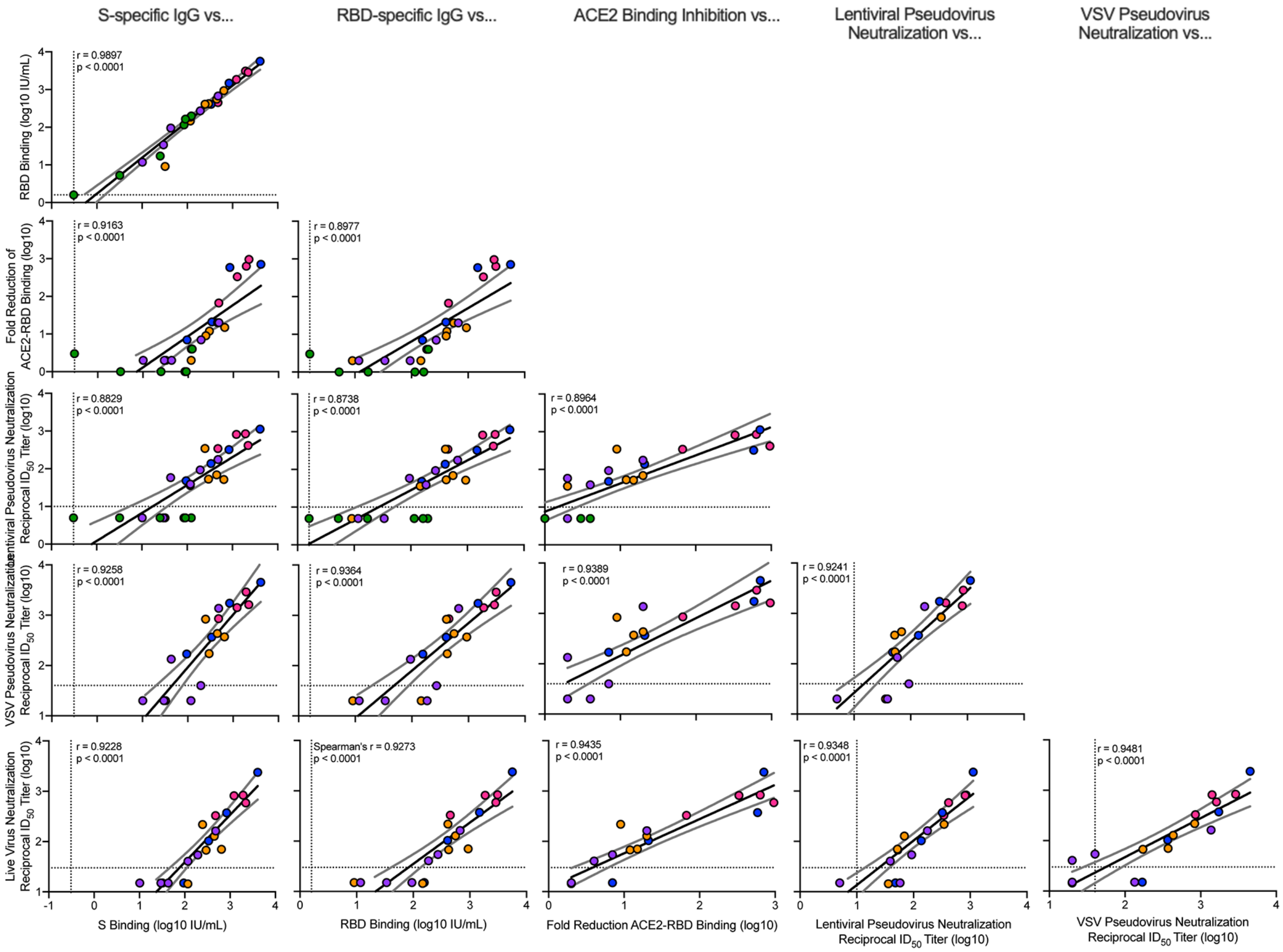

Fig. 2. Correlations of humoral antibody analyses. Rhesus macaques were immunized according to fig. S1C. Plots show correlations between SARS-CoV-2 S-specific IgG, RBD-specific IgG, ACE2 binding inhibition, lentiviral-based pseudovirus neutralization, VSV-based pseudovirus neutralization, and EHC-83E focus reduction neutralization at 4 weeks post-boost. Circles represent individual NHPs, where colors indicate the mRNA-1273 dose as defined in fig. S1C. Dotted lines indicate assay limits of detection. Black and gray lines indicate linear regression and 95% confidence interval, respectively. "r" represents Spearman's correlation coefficients and "*P*" represents the corresponding *P*-values.

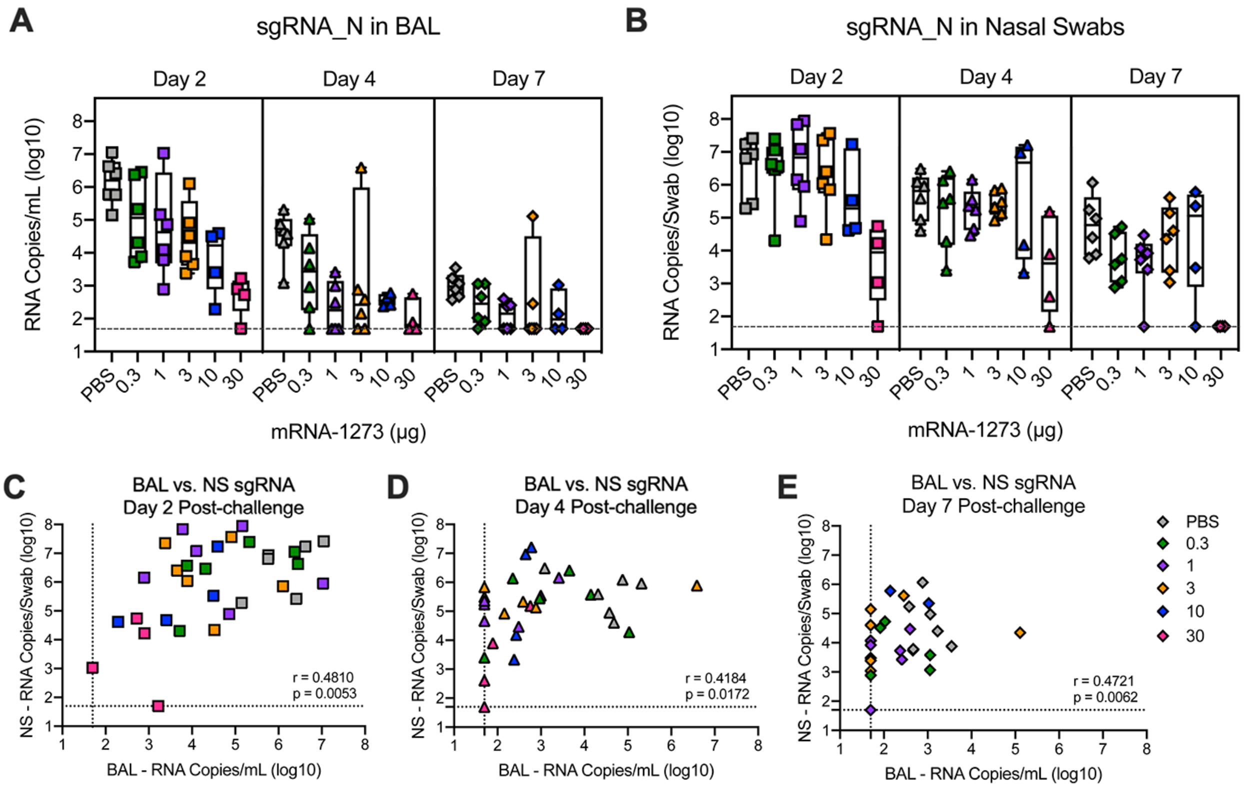

Fig. 3. Efficacy of mRNA-1273 against upper and lower respiratory viral replication. (**A** and **B**) Rhesus macaques were immunized and challenged as described in fig. S1C. BAL (A) and nasal swabs (NS) (B) were collected on days 2 (squares), 4 (triangles), and 7 (diamonds) post-challenge, and viral replication was assessed by detection of SARS-CoV-2 N-specific sgRNA. (A and B) Boxes and horizontal bars denote the IQR and medians, respectively. Whisker endpoints are equal to the maximum and minimum values. (**C** to **E**) Correlations shown between BAL and NS sgRNA at days 2 (C), 4 (D), and 7 (E) post-challenge are Spearman's correlation coefficients (r) and corresponding *P*-values. Symbols represent individual NHP and may overlap, i.e,. n=6 animals plotted at assay limit (dotted line) for both BAL and NS in (E).

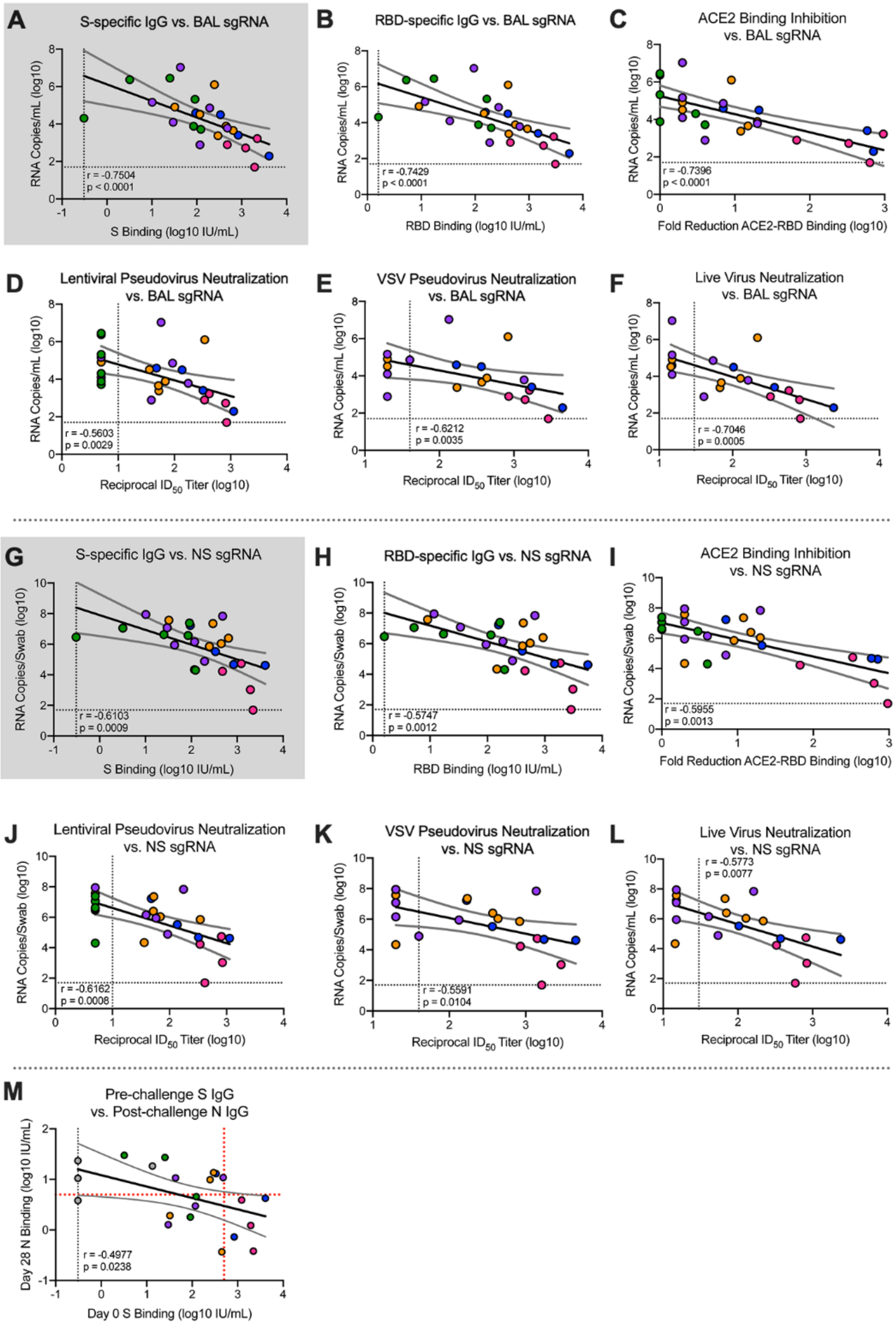

Fig. 4. Antibody correlates of protection. Rhesus macaques were immunized and challenged as described in fig. S1C. Plots show correlations between SARS-CoV-2 N-specific sgRNA in BAL (**A** to **F**) and NS (**G** to **L**) at day 2 post-challenge and pre-challenge (week 4 post-boost) SARS-CoV-2 S-specific IgG (A and G), RBD-specific IgG (B and H), ACE2 binding inhibition (C and I), SARS-CoV-2 lentiviral-based pseudovirus neutralization (D and J), SARS-CoV-2 VSV-based pseudovirus neutralization (E and K) and SARS-CoV-2 EHC-83E focus reduction neutralization (F and L). Gray shading for S-specific IgG represents the use of this assessment as primary predictor of protection outcome as stated in primary hypothesis. (M) Plot shows correlation between pre-challenge (week 4 post-boost) SARS-CoV-2 S-specific IgG with day 28 post-challenge SARS-CoV-2 N-specific IgG. Circles represent individual NHPs, where colors indicate the mRNA-1273 dose. Dotted lines indicate assay limits of detection. Black and gray lines indicate linear regression and 95% confidence interval, respectively. In (**M**), a red dotted horizontal line represents 6, the maximum of all pre-challenge values across all groups, and a red dotted vertical line represents a reciprocal S-specific IgG titer of 500, above which none of the animals had day 28 N Binding titers above 6. "r" represents Spearman's correlation coefficients and "*P*" represents the corresponding *P*-values.

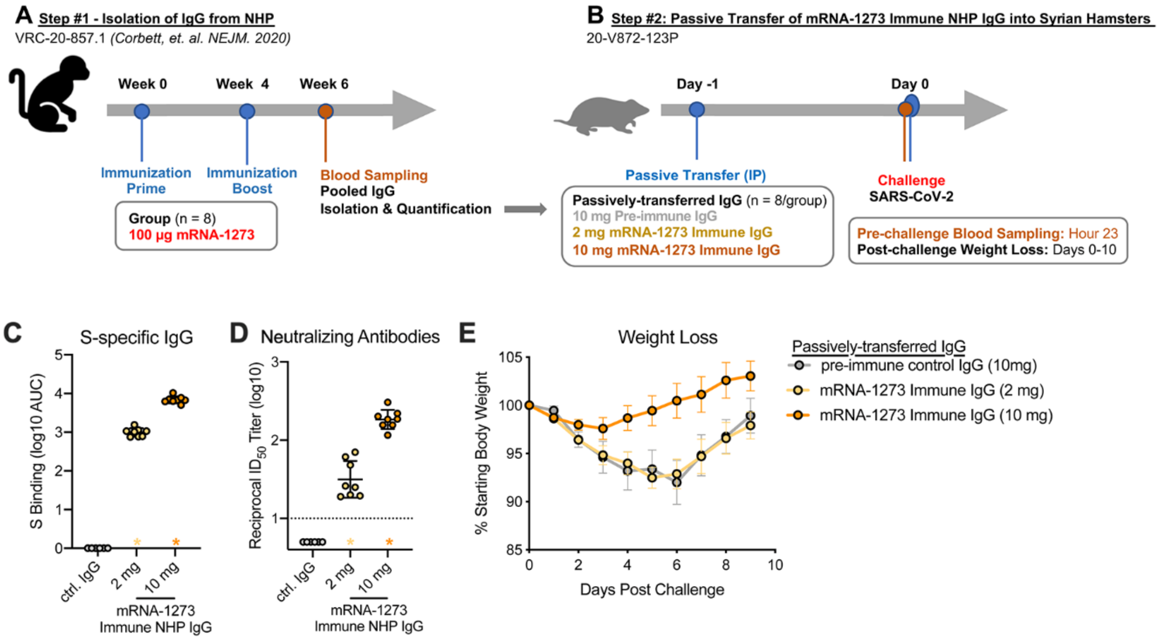

Fig. 5. Passive transfer of mRNA-1273 immune NHP IgG into Syrian hamsters. (**A**) Sera were pooled from all NHP that received 100 µg of mRNA-1273 in a primary vaccination series. (**B**) mRNA-1273 immune NHP IgG (2 mg, yellow or 10 mg, orange) or pre-immune NHP IgG (10 mg, gray) was passively transferred to Syrian hamsters (n=8/group) 24 hours prior to SARS-CoV-2 challenge. (**C** and **D**) Twenty-three hours post-immunization, hamsters were bled to quantify circulating S-specific IgG (C) and SARS-CoV-2 pseudovirus-neutralizing antibodies (D). (**E**) Following challenge, hamsters were monitored for weight loss. In (C) and (D), circles represent individual NHP. Bars and error bars represent GMT and geometric SD, respectively. Asterisks at the axis represent animals that did not receive adequate IgG via passive transfer and were thus excluded from weight loss analyses. (D) The dotted line indicates the neutralization assay limit of detection. (E) Circles and error bars represent means and SEM, respectively.
