# Supplementary material for: Immune correlates of protection by mRNA-1273 vaccine against SARS-CoV-2 in nonhuman primates
Source: Science. 2021 Jul 29;373(6561):eabj0299. doi: 10.1126/science.abj0299 (PMC8449013; doi:10.1126/science.abj0299)
Supplement: Supplementary file 2 — Figs. S1 to S11 Tables S1 to S5 [file science.abj0299_sm.pdf]

Science
AAAS

Supplementary Materials for

Immune correlates of protection by mRNA-1273 vaccine against SARS-CoV-2 in nonhuman primates

Kizzmekia S. Corbett *et al.*

Corresponding authors: Barney S. Graham, bgraham@nih.gov; Robert A. Seder, rseder@mail.nih.gov

Science **373**, eabj0299 (2021)
DOI: 10.1126/science.abj0299

The PDF file includes:

Figs. S1 to S11
Tables S1 to S5

Other Supplementary Material for this manuscript includes the following:

MDAR Reproducibility Checklist

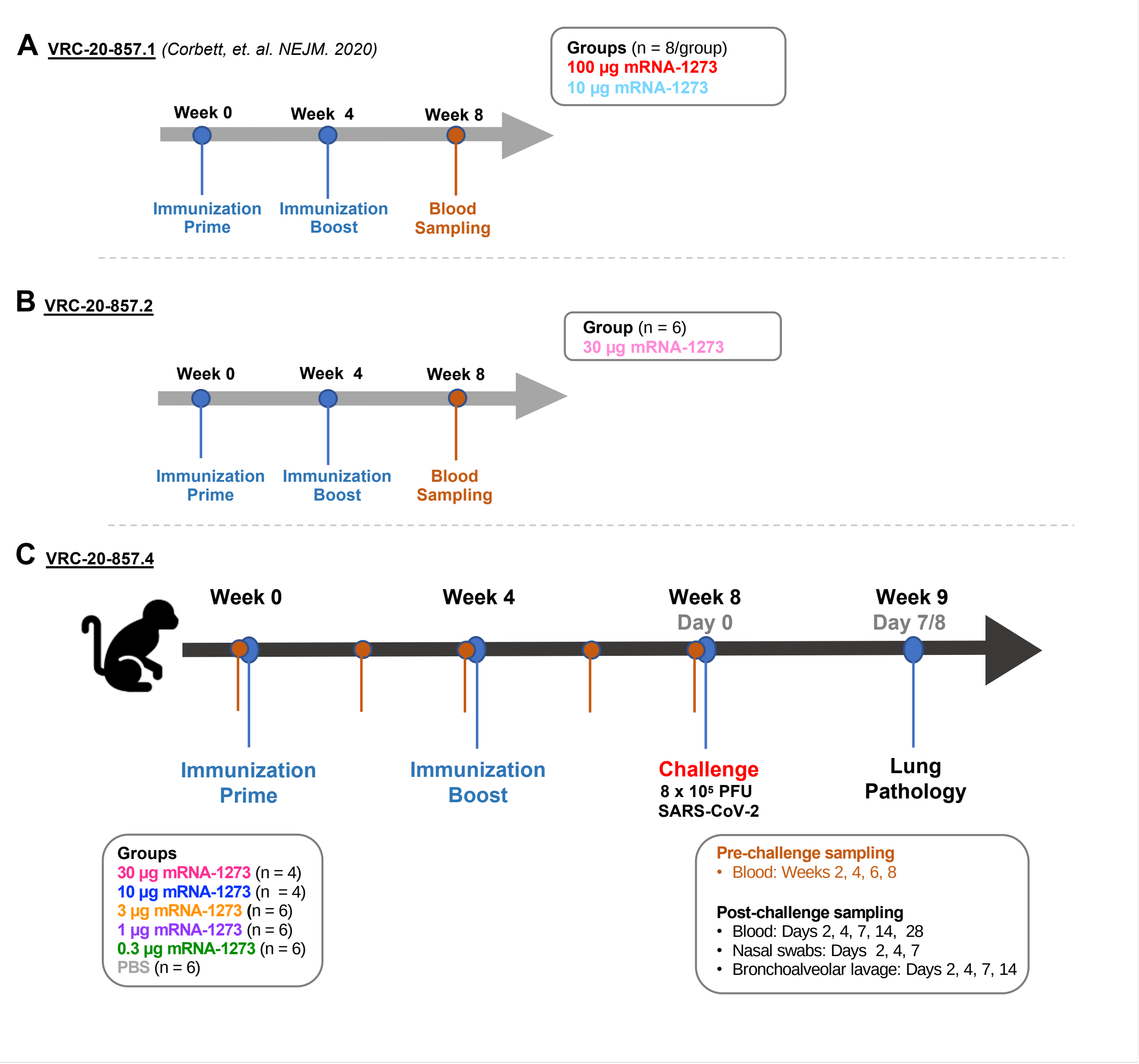

Fig. S1. Study design: Evaluation of immune correlates of protection of mRNA-1273 in rhesus macaques. (A and B) The design of the correlates of protection study (C) was informed by previous studies to assess the immunogenicity of various doses of mRNA-1273 in NHPs. (C) To assess immune correlates of protection, Rhesus macaques were immunized at 0 and 4 weeks with PBS or various doses of mRNA-1273 and challenged 4 weeks post-boost with a total of 8×10^5 PFU of SARS-CoV-2. The viral inoculum was administered as 6×10^5 PFU in 3 ml intratracheally and 2×10^5 PFU in 1 ml intranasally (0.5 ml into each nostril). Sera were collected pre-

immunization and bi-weekly post-prime and post-boost. Sera, nasal washes, and bronchoalveolar lavages were collected post-challenge on days 2, 4, 7, 14, and 28. Lung pathology was assessed on days 7 and 8 post-challenge in a subset of animals (n=1 or 2 per group). Pre-challenge serological assessments are overlayed within applicable figures where squares represent experiments VRC-20-857.1 (A) and VRC-20-857.2 (B), and circles represent experiment VRC-20-857.4 (C).

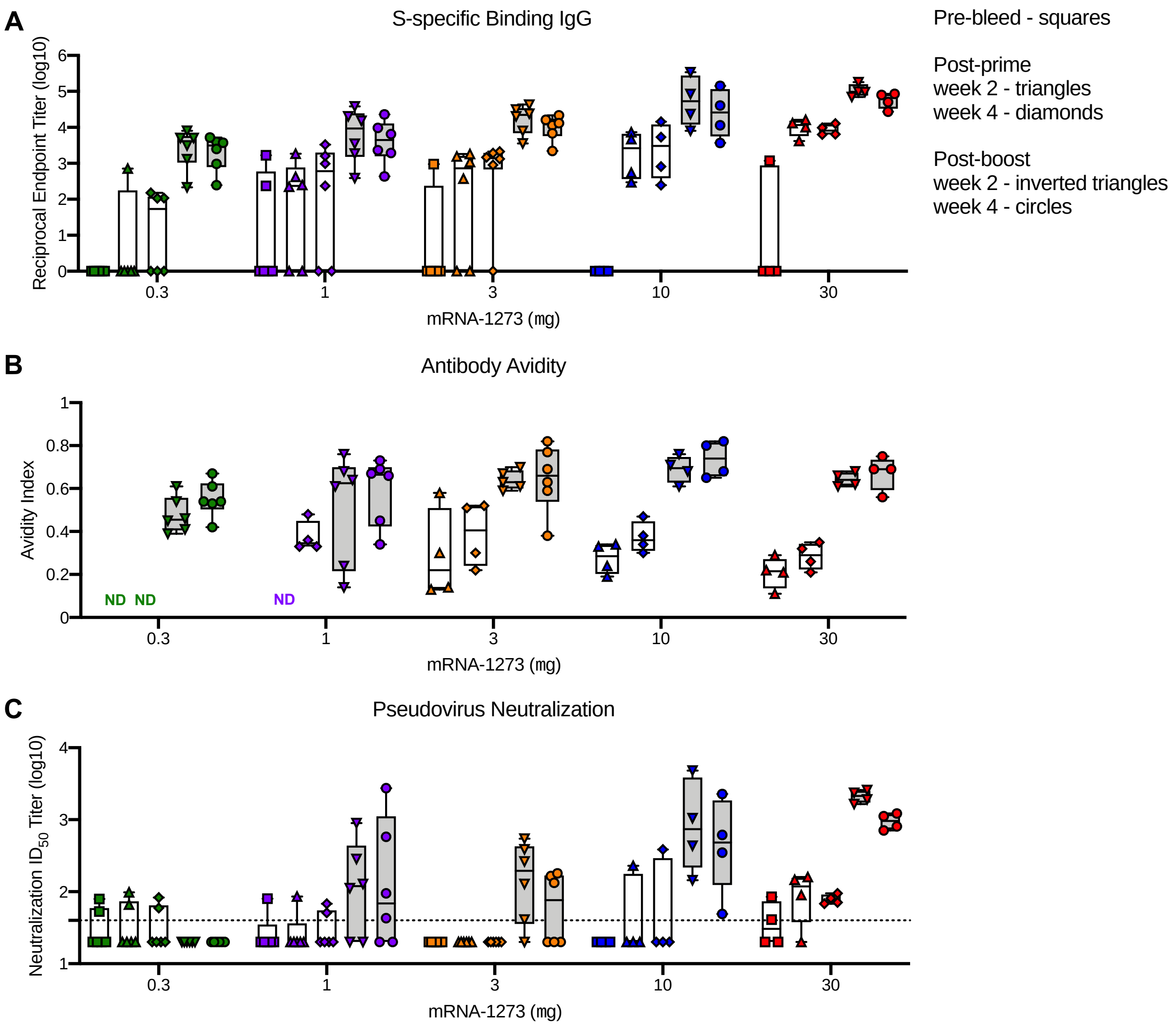

<u>Fig. S2</u>. Temporal serum antibody responses following mRNA-1273 immunization. Rhesus macaques were immunized according to Fig. S1C. Sera collected at week 0 (pre-bleed, unfilled bars) and 2- and 4-weeks post-prime (unfilled bars) and post-boost (filled bars) and subsequently assessed for SARS-CoV-2 S-specific IgG (A), antibody avidity (B), and SARS-CoV-2 D614G lentiviral-based pseudovirus neutralization (C). Symbols represent individual NHPs. Boxes and horizontal bars denote interquartile ranges (IQR) and medians, respectively. Whisker endpoints are equal to the maximum and minimum values. Dotted lines indicate assay limit of detection, where applicable. ND: not determined due to low-level S-specific binding antibody titers.

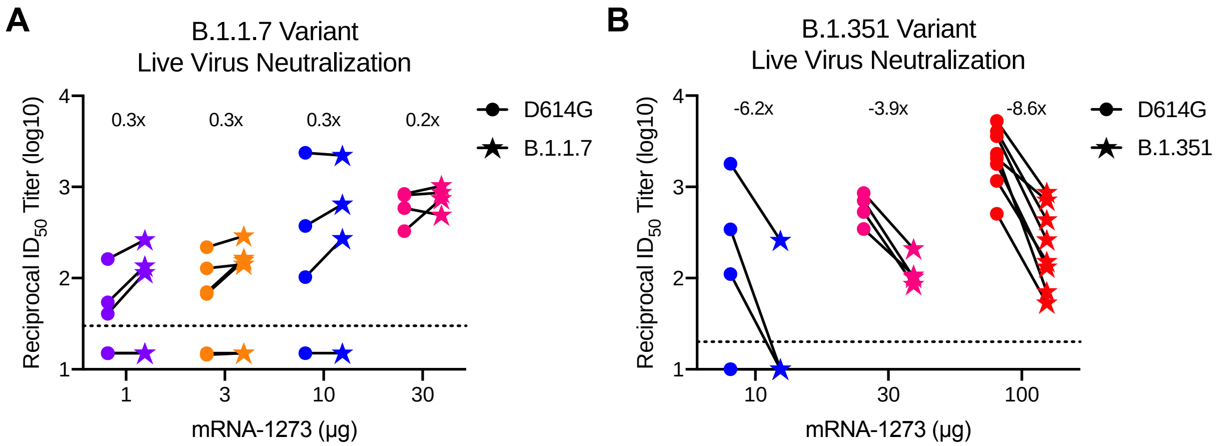

<u>Fig. S3</u>. Ability of mRNA-1273 immune NHP serum to neutralize global variants. Rhesus macaques were immunized according to Fig. S1C. Sera collected 4-weeks post-boost, were assessed for focus reduction neutralization using D614G-encompassing SARS-CoV-2 EHC-83E compared to B.1.1.7 (A) and B.1.351 (B) SARS-CoV-2 variants. Symbols represent individual NHPs. For each immunogen group, the geometric mean titer (GMT) for each variant was compared to D614G and the fold difference is indicated above respective distributionsd.

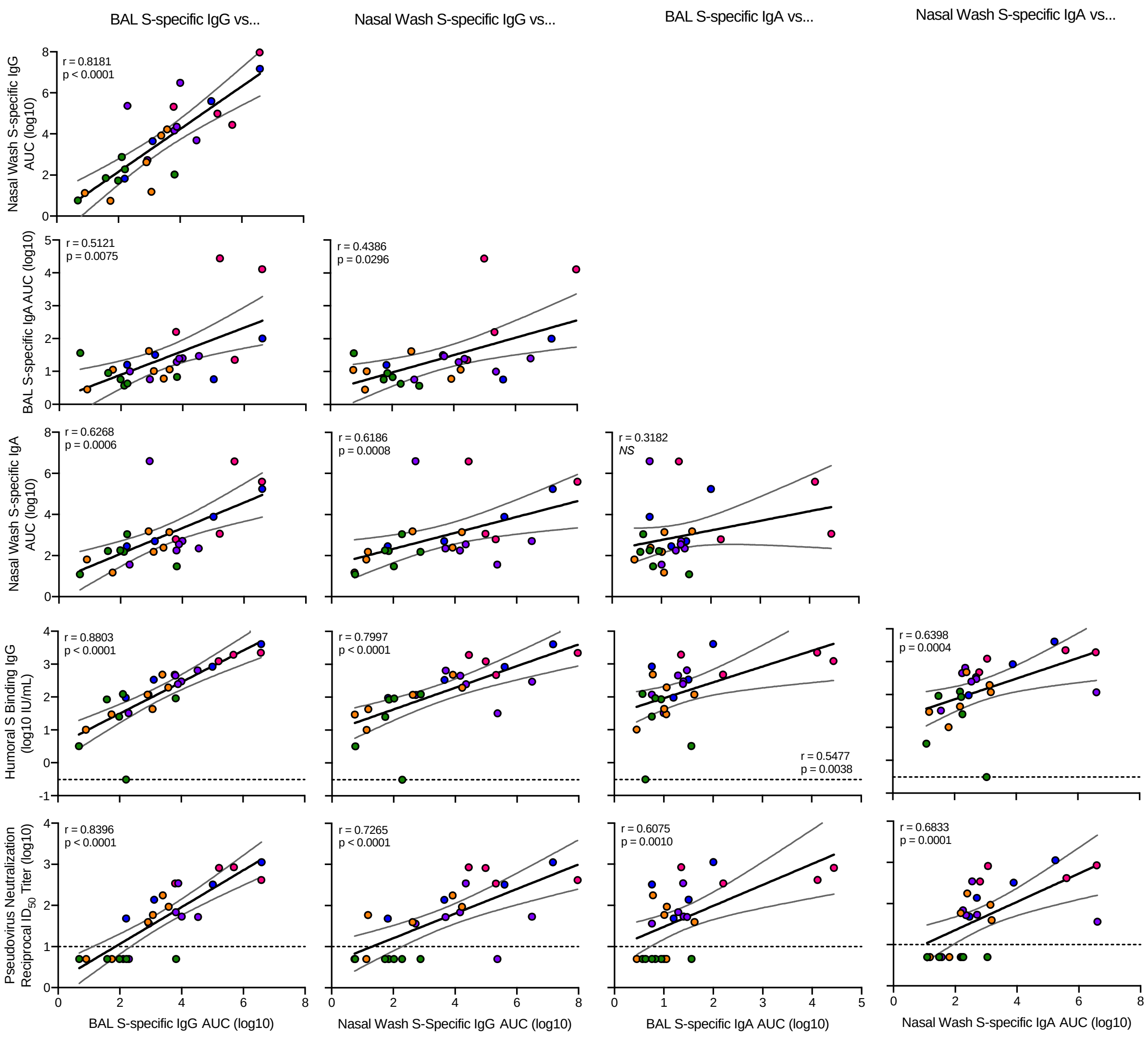

Fig. S4. **Correlations of mucosal antibody responses to humoral antibody responses.** Rhesus macaques were immunized according to Fig. S1C. Plots show correlations between S-specific BAL IgG, nasal wash IgG, BAL IgA, and nasal wash IgA at week 2 post-boost with each other and with humoral S-specific IgG and lentiviral-based pseudovirus neutralization at week 4 post-boost. Circles represent individual NHPs, where colors indicate mRNA-1273 dose. Dotted lines indicate assay limits of detection. Black and gray lines indicate linear regression and 95% confidence interval, respectively. “r” represents Spearman’s correlation coefficients, and “*P*” the corresponding *P*-values.

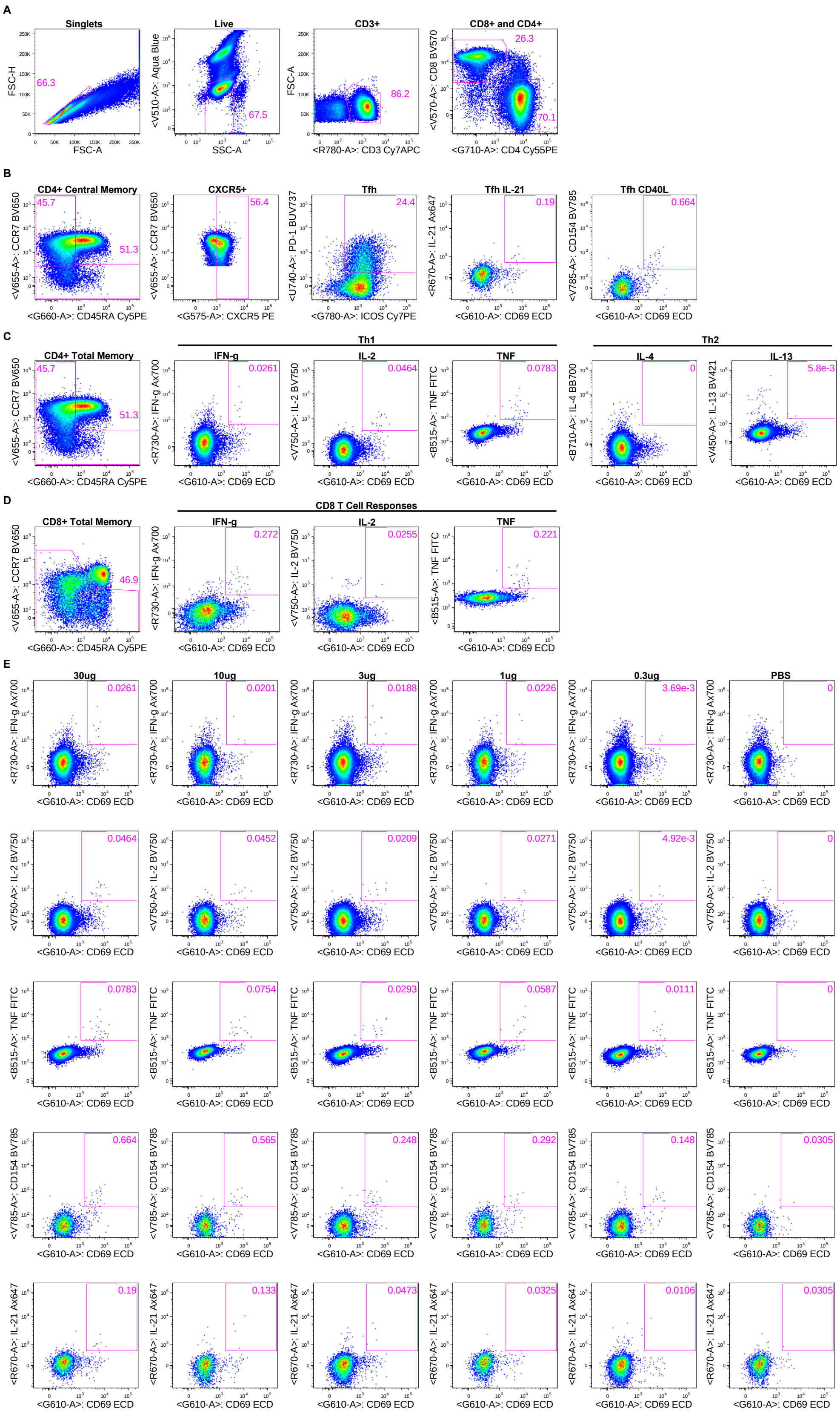
A
Singlets
Live
CD3+
CD8+ and CD4+
B
CD4+ Central Memory
CXCR5+
Tfh
Tfh IL-21
Tfh CD40L
C
CD4+ Total Memory
Th1
IFN-g
IL-2
TNF
Th2
IL-4
IL-13
D
CD8+ Total Memory
CD8 T Cell Responses
IFN-g
IL-2
TNF
E
30ug
10ug
3ug
1ug
0.3ug
PBS

Fig S5. Gating tree and representative staining for S-specific Th1, Th2, Tfh and CD8 responses. Flow cytometry plots from a representative NHP that showed Th1 and CD8 T cell cytokine responses to best display staining of all populations and production of all cytokines (A-D). (A) T cell populations were selected by subsequent gating: first single cells, followed by live/SSC low cells, then $CD3^+$/FSC low cells, and finally $CD4^+$ and $CD8^+$ T cells. (B) To identify Tfh, CD4 T cells were gated first on central memory (CM) T cells ($CCR7^+CD45RA^-$) then $CXCR5^+$ CM cells followed by $PD\text{-}1^+ICOS^+$ cells. Then, to measure IL-21 production and CD40L (CD154) expression from Tfh, $CD69^+IL\text{-}21^+$ and $CD69^+CD154^+$ cells were gated. (C and D) Cytokine production from total memory CD4 and CD8 T cells was measured by first gating on total memory T cells (central memory ($CCR7^+CD45RA^-$), effector memory ($CCR7^-CD45RA^-$), and terminal effector memory ($CCR7^-CD45RA^+$) T cells), then $CD69^+$cytokine$^+$ cells. (E) Representative Th1 and Tfh plots from a one animal in each mRNA 1273 vaccine dose group.

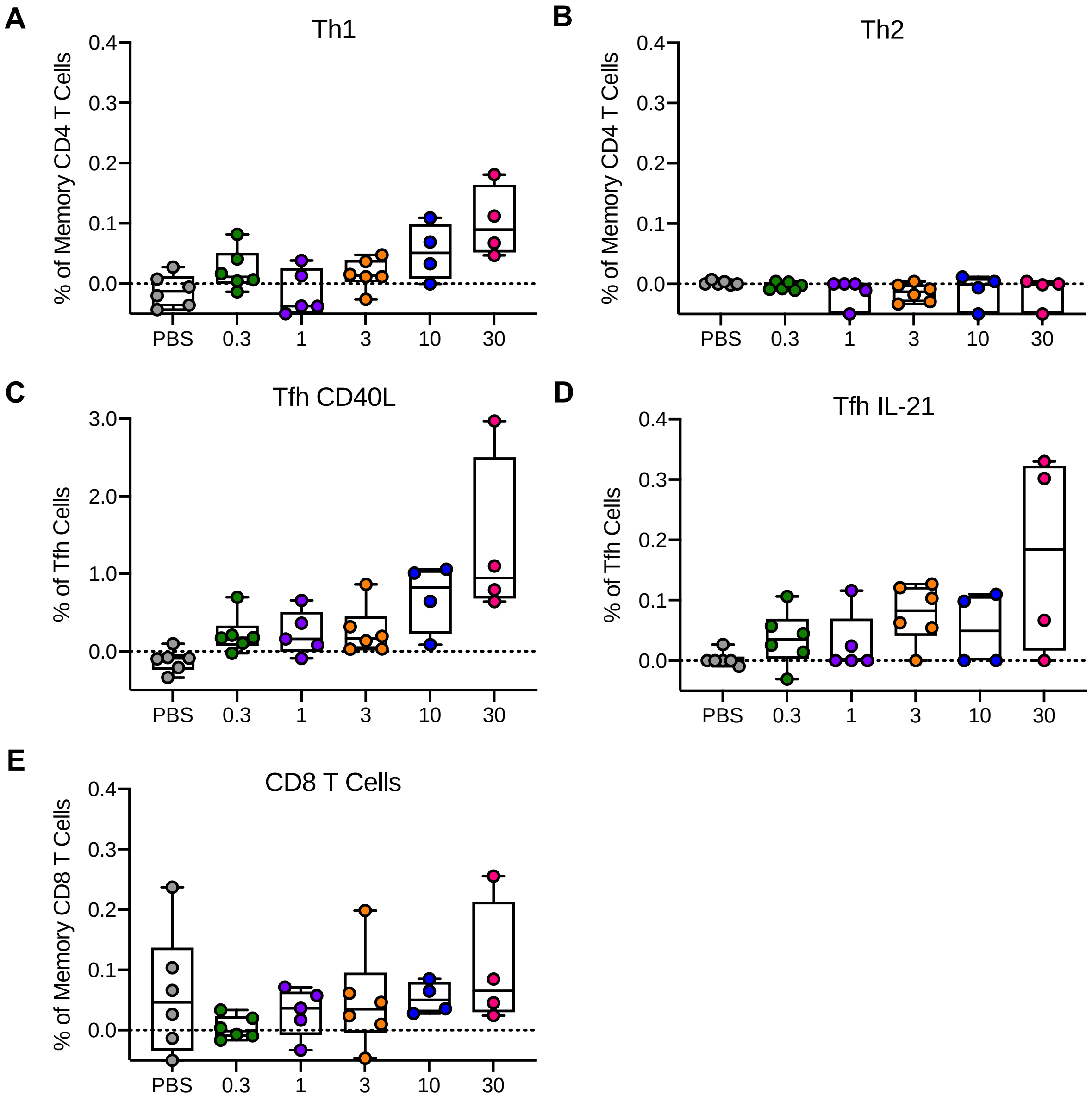

Fig. S6. T cell responses following mRNA-1273 immunization. Rhesus macaques were immunized according to Fig. S1C. Intracellular staining was performed on PBMCs as described in Fig.S5Methods at week 6 (2 weeks following boost), to assess T cell responses to SARS-CoV-2 S S1 and S2 peptide pools. Background-subtracted responses to S1 and S2 individual peptide pools were summed. (A) Th1 responses (IFNg, IL-2, or TNF), (B) Th2 responses (IL-4 or IL-13), (C) Tfh CD40L upregulation (peripheral follicular helper T cells (Tfh) were gated on central

memory $CXCR5^+PD\text{-}1^+ICOS^+$ CD4 T cells), (D) Tfh IL-21, and (E) CD8 T cell responses (IFNg, IL-2, or TNF) were assessed. Boxes and horizontal bars denote IQR and medians, respectively. Whisker endpoints are equal to the maximum and minimum values. Circles represent individual NHPs. Dotted lines are set to 0%.

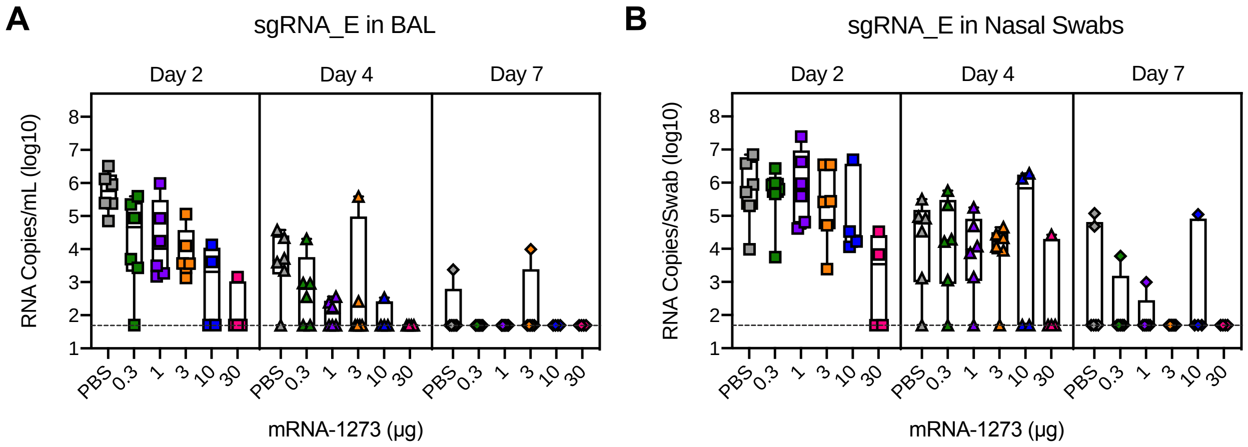

<u>Fig. S7</u>. Efficacy of mRNA-1273 against upper and lower respiratory viral replication. Rhesus macaques were immunized and challenged as described in Fig. S1C. BAL (A) and NS (B) were collected on days 2 (squares), 4 (triangles), and 7 (diamonds) post-challenge, and viral replication was assessed by detection of SARS-CoV-2 E-specific sgRNA. Boxes and horizontal bars denote the IQR and medians, respectively; whisker end points are equal to the maximum and minimum values. Symbols represent individual NHPs and overlap for equal values where constrained. Dotted lines indicate assay limits of detection.

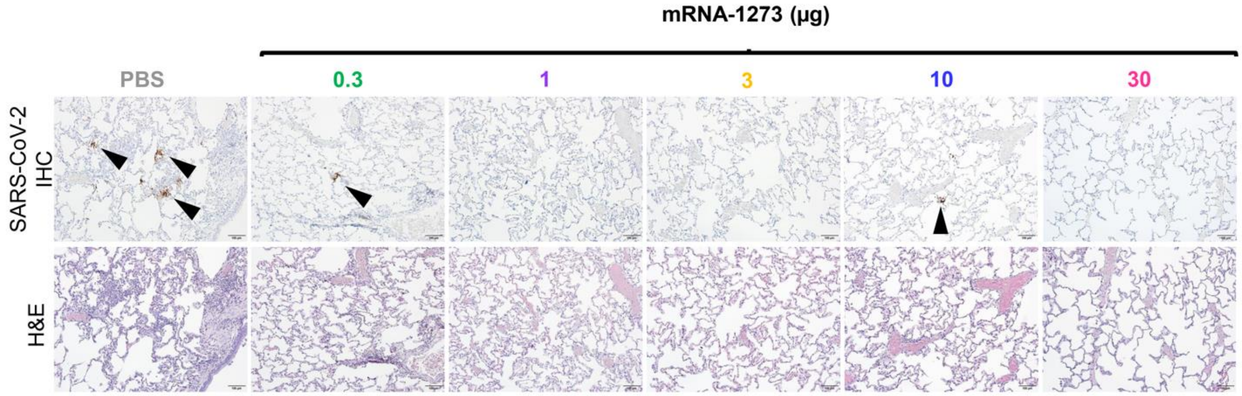

<u>Fig. S8</u>. Post-challenge lung histopathological analysis and viral detection. Rhesus macaques were immunized and challenged as described in Fig. S1C. Seven days after challenge, lung samples (n=1 per group) were evaluated for evidence of virus infection (top) and the presence of inflammation (bottom). Representative images show the location and distribution of SARS-CoV-2 virus antigen by IHC in serial lung tissue sections. Arrows indicate areas positive for viral antigen. Each image is taken at 10X magnification. Scale bars represent 100 µm.

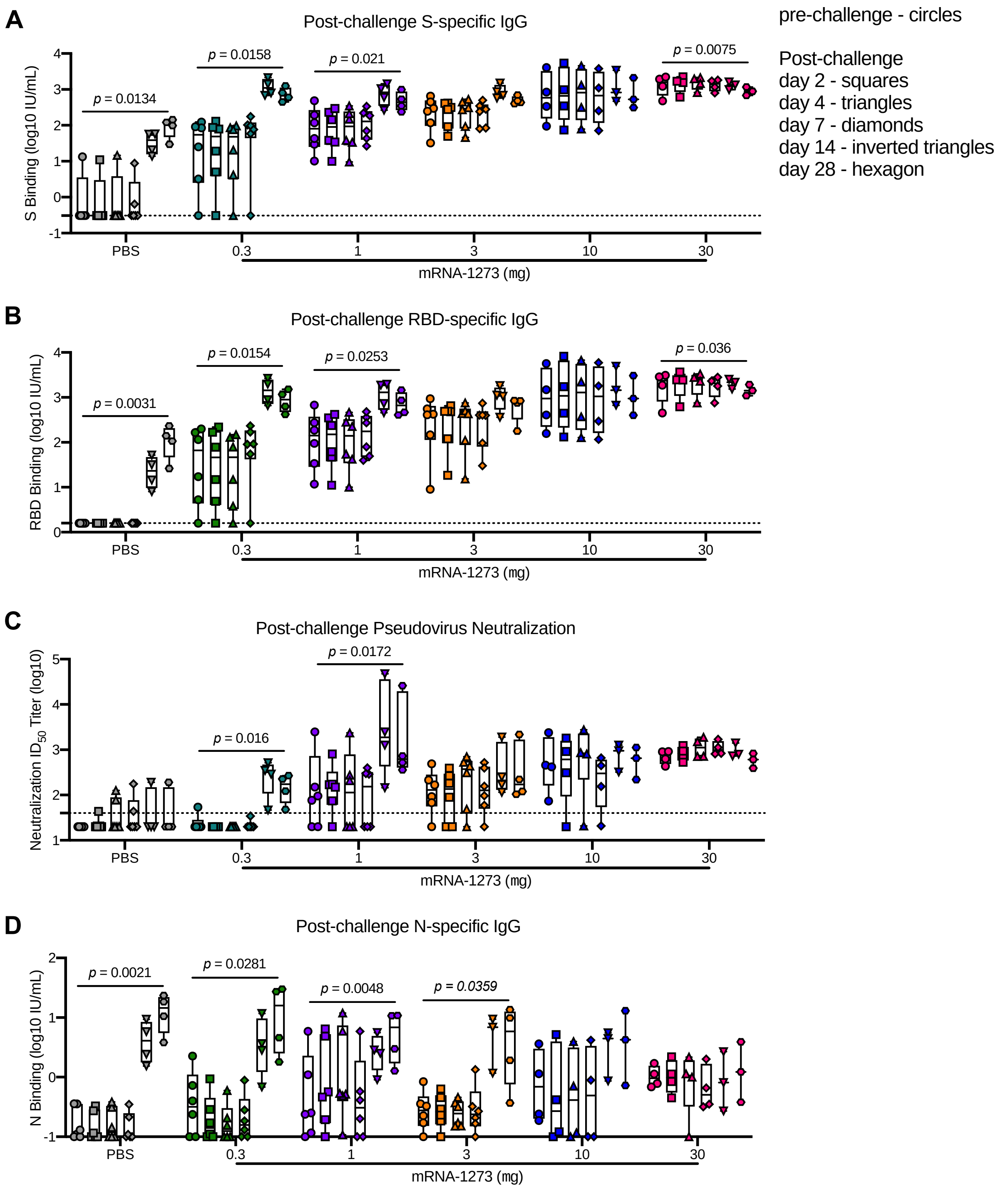

<u>**Fig. S9**</u>**. Humoral antibody responses following SARS-CoV-2 challenge in mRNA-1273-immunized NHPs.** Rhesus macaques were immunized and challenged according to Fig. S1C. Sera collected 4 weeks post-boost, immediately prior to challenge, and days 2, 4, 7, 14, and 28 post-

challenge were assessed for SARS-CoV-2 S-specific (A), RBD-specific (B), and N-specific (D) IgG by MULTI-ARRAY ELISA and SARS-CoV-2 D614G lentiviral-based pseudovirus neutralization (C). Boxes and horizontal bars denote the IQR and medians, respectively. Whisker endpoints are equal to the maximum and minimum values. Symbols represent individual NHPs. Dotted lines indicate assay limits of detection. Significance between pre-challenge and the final timepoint post-challenge determined by paired *t* tests.

pre-challenge - circles

Post-challenge
day 2 - squares
day 4 - triangles
day 7 - diamonds
day 14 - inverted triangles

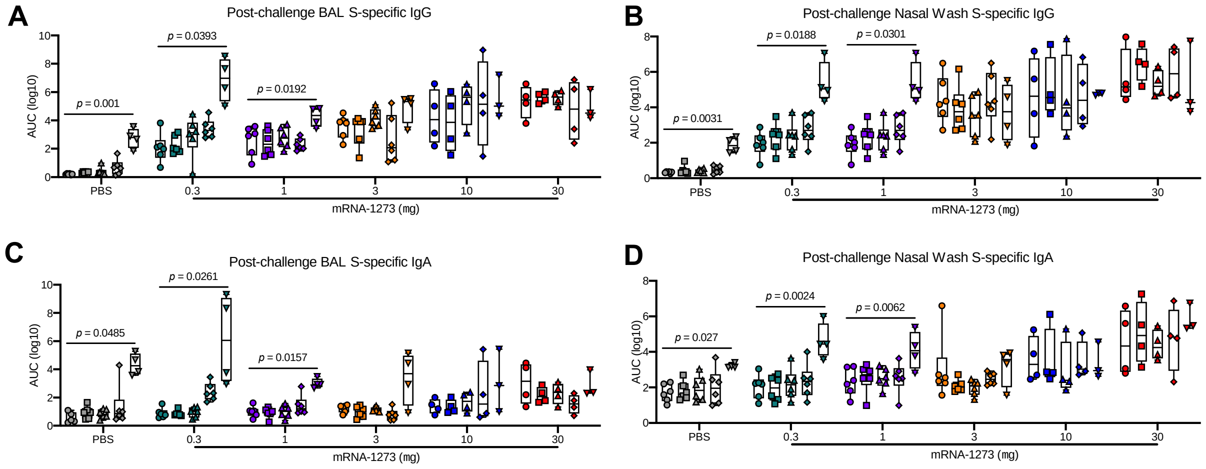

<u>Fig. S10</u>. Mucosal antibody responses following SARS-CoV-2 challenge in mRNA-1273-immunized NHPs. Rhesus macaques were immunized and challenged according to Fig. S1C. BAL (A and C) and nasal washes (B and D) collected 2 weeks post-boost and days 2, 4, 7, and 14 post-challenge were assessed for SARS-CoV-2 S-specific IgG (A and B) and IgA (C and D) by MULTI-ARRAY ELISA. Boxes and horizontal bars denote the IQR and medians, respectively. Whisker endpoints are equal to the maximum and minimum values. Circles represent individual NHPs. Significance between pre-challenge and the final timepoint post-challenge determined by paired *t* tests.

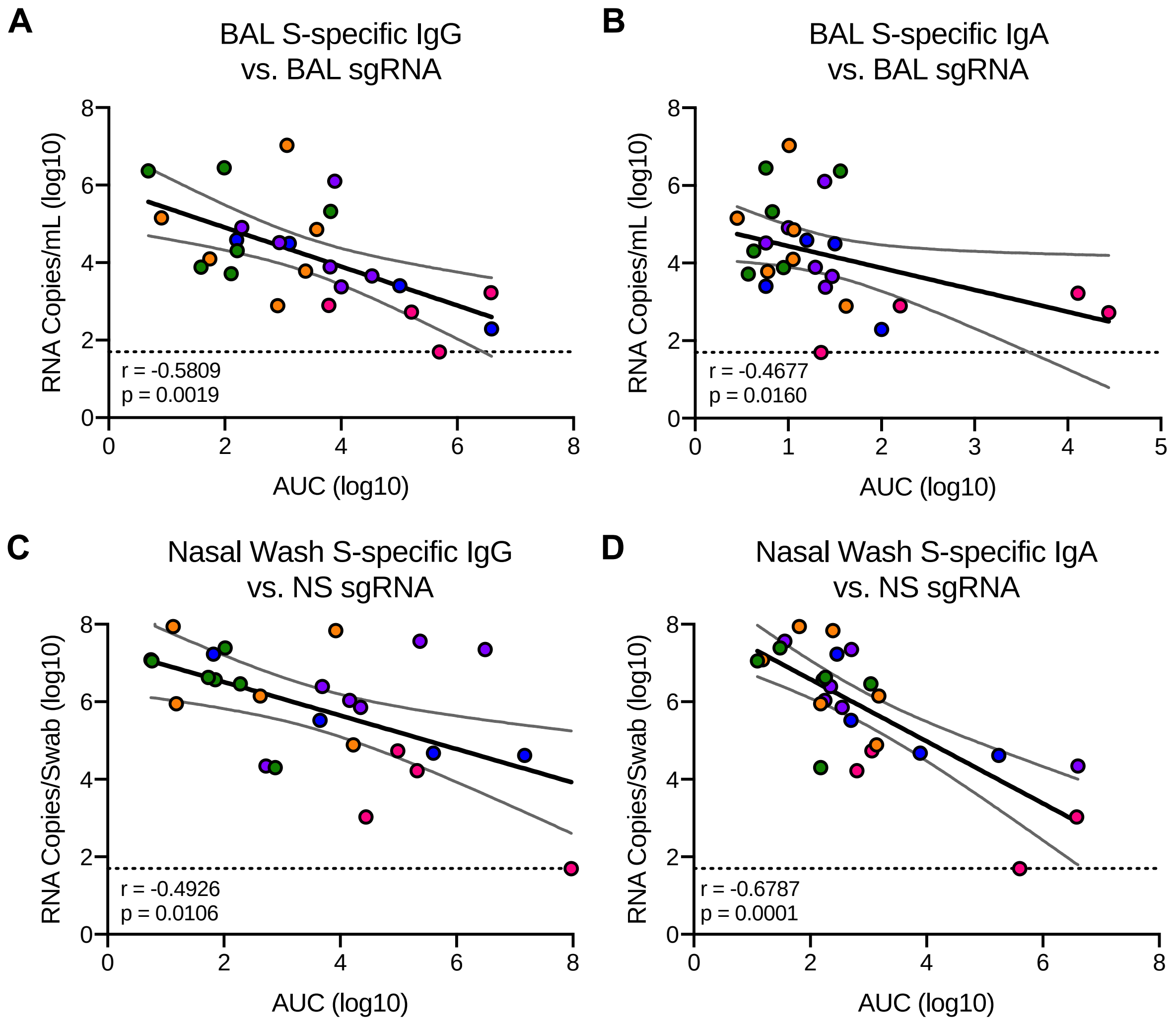

<u>Fig. S11</u>. Correlations of mucosal antibody responses and viral replication. Rhesus macaques were immunized and challenged according to Fig. S1C. Plots show correlations between BAL (A and B) and NS (C and D) S-specific IgG (A and C) and IgA (B and D) at week 2 post-boost with SARS-CoV-2 N-specific sgRNA in BAL (A and B) and NS (C and D) at day 2 post-challenge. Circles represent individual NHPs, where colors indicate mRNA-1273 dose. Dotted lines indicate PCR limit of detection. Black and gray lines indicate linear regression and 95% confidence interval, respectively. "r" represents Spearman's correlation coefficients, and "*P*" the corresponding *P*-values.

NHP ID	Post-challenge (Day)	mRNA-1273 (µg)	Inflammation (H&E)[1]	SARS-CoV-2 Ag[2] (Lc[3]; Rmid[4]; Rc[5])
**15D043**	**7**	**PBS**	**++**	**−; +; +/−**
**A15V047**	**8**	**PBS**	**++**	**+/−; −; +/−**
**LP73**	**7**	**0.3**	**+**	**−; −; +/ −**
**G45R**	**8**	**0.3**	**++**	**−; −; −**
**A16V036**	**7**	**1**	**+**	**−; −; −**
**A15V075**	**8**	**1**	**+**	**+/−; −; −**
**16C282**	**7**	**3**	**+**	**−; −; −**
**15D025**	**8**	**3**	**+**	**−; −; +/−**
**A15V037**	**7**	**10**	**+/−**	**−; −; +/−**
**16C225**	**7**	**30**	**+/−**	**−; −; −**

Table S1. Assessment of lung inflammation and viral antigen following SARS-CoV-2 challenge of mRNA-1273-immunized NHPs.

Lung tissue was evaluated for the presence of inflammation and SARS-CoV-2 viral antigen.

[1] Inflammation scoring: +/− = minimal to mild, + = mild to moderate, ++ = moderate to severe

[2] Immunohistochemistry SARS-CoV-2 antigen (Ag): − = no detection of virus Ag, +/− = rare/occasional Ag^+ foci, + = multiple Ag^+ foci

[3] Lc: left caudal lung lobe

[4] Rmid: right middle lung lobe

[5] Rc: right caudal lung lobe

A

Linear Regression

Outcome	Week 4 Post-boost Antibody Measurement	Univariate: Outcome ~ Antibody measure — Beta Antibody	*P*-value Antibody	AdjR^2	Multivariate: Outcome ~ Antibody measure + dose — Beta Antibody	*P*-value Antibody	Beta dose	*P*-value dose	Adj R^2	Antibody measurement meets criteria for Potential CoP?
BAL Day 2 sgRNA_N	S-specific IgG	-0.885	0.001	0.35	-0.581	0.093	-0.554	0.214	0.36	Yes
	RBD-specific IgG	-0.936	<0.001	0.38	-0.659	0.050	-0.513	0.223	0.39	Yes
	ACE2 Binding Inhibition	-0.957	<0.001	0.49	-1.029	0.008	0.122	0.812	0.47	Yes
	Lentivirus Pseudovirus Neutralization	-0.844	0.005	0.25	-0.252	0.606	-0.849	0.153	0.29	Yes
	VSV Pseudovirus Neutralization	-0.758	0.031	0.19	-0.256	0.553	-1.066	0.103	0.27	Yes
	Live Virus Neutralization	-1.218	0.001	0.41	-0.933	0.053	-0.515	0.371	0.41	Yes
NS Day 2 sgRNA_N	S-specific IgG	-0.966	0.003	0.29	-0.499	0.222	-0.851	0.115	0.34	Yes
	RBD-specific IgG	-1.047	0.001	0.34	-0.645	0.105	-0.745	0.142	0.37	Yes
	ACE2 Binding Inhibition	-1.098	<0.001	0.47	-1.065	0.021	-0.056	0.928	0.44	Yes
	Lentivirus Pseudovirus Neutralization	-1.157	0.001	0.36	-0.770	0.168	-0.555	0.399	0.35	Yes
	VSV Pseudovirus Neutralization	-1.027	0.023	0.22	-0.133	0.789	-1.898	0.017	0.41	No
	Live Virus Neutralization	-1.497	0.003	0.37	-0.718	0.208	-1.41	0.056	0.46	marginal

B

		Linear Regression								
		Univariate<br>Outcome ~ T-cell measure			Multivariate<br>Outcome ~ T-cell measure + S-specific IgG					T-cell measurement predictive after adjusting for S-specific IgG?
**Outcome**	**Week 2 Post-boost T cell Measurement**	**Beta predictor**	**p-value predictor**	**Adj R^2**	**Beta predictor**	***P*-value predictor**	**Beta S-IgG**	***P*-value S-IgG**	**Adj R^2**	
BAL Day 2 sgRNA_N	IL-21	-4.527	0.080	0.09	-0.471	0.812	-1.326	<0.001	0.54	No
	CD40L	-1.284	0.008	0.24	-0.313	0.459	-1.226	<0.001	0.55	No
	Any Th1	-17.106	0.004	0.28	-4.874	0.364	-1.184	0.001	0.55	No
	Any Th2	-74.749	0.011	0.22	-12.775	0.622	-1.265	<0.001	0.54	No
NS Day 2 sgRNA_N	IL-21	-10.403	0.000	0.46	-7.801	0.002	-0.851	0.011	0.59	Yes
	CD40L	-2.151	0.000	0.53	-1.638	0.002	-0.647	0.071	0.57	Yes
	Any Th1	-22.922	0.001	0.38	-14.489	0.045	-0.816	0.051	0.45	marginal
	Any Th2	-67.906	0.055	0.11	-4.649	0.898	-1.291	0.007	0.34	No

Table S2. **Summary of linear regression models examining relationships.** (A) Antibody measurements at week 4 post-boost and sgRNA in BAL or NS, with or without adjustment for dose. (B) T cell subsets at week 2 post-boost and sgRNA in BAL or NS, with or without adjustment for S-specific IgG. In all models, sgRNA, antibody measures, and dose are modeled on the $\log_{10}$ scale. Antibody measures are considered to meet the criteria for potential correlates of protection from high sgRNA if they are significantly associated with sgRNA univariately and if dose is not statistically significant after adjustment for that potential antibody measure in a multivariate regression model. Gray shading for S-specific IgG represents the pre-specified primary correlate of interest.

A

Variant	MUTLI-ARRAY ELISA
**WA-1**	*No mutations*

B

Variant	Pseudovirus	
	**Lentiviral**	**VSV**
**WA-1**	*N/A*	*N/A*
**D614G**	614G	614G

C

Variant	Live Virus
**WA-1**	*N/A*
**D614G**	614G
**B.1.1.7**	Δ69-70, Δ144, 501Y, 570D, 614G, 681H, 716I, 982A, 1118H
**B.1.351**	18F, 80A, 215G, Δ242-244, 417N, 484K, 501Y, 614G, 701V

<u>Table S3</u>. Spike mutations included in MULTI-ARRAY ELISA (A), pseudovirus (B) and live-virus (C) assay reagents as compared to Wuhan-1 strain (Genbank #: MN908947.3).

[1]N/A: not applicable

Table S4. Glossary of Terms

Abbreviation	Definition
**ACE2**	Angiotensin-converting enzyme 2; host receptor for SARS-CoV-2
**AUC**	Area under the curve
**B.1.1.7**	Alpha variant of SARS-CoV-2; specific mutations denoted in Table S3
**B.1.351**	Beta variant of SARS-CoV-2; specific mutations denoted in Table S3
**BAL**	Bronchoalveolar lavage
**BNT162b2**	mRNA vaccine by Pfizer/BioNTech
**CD40L**	CD4+ T cell marker
**COVID-19**	Coronavirus disease 2019
**D614G**	Mutation of residue 614 in SARS-CoV-2 spike protein from aspartate to glycine
**E**	Viral envelope gene
**EHC-83E**	D614G variant of SARS-CoV-2, isolated at Emory University in Georgia USA
**ELISA**	Enzyme Linked Immunosorbent Assay
**IgA**	Immunoglobulin A
**IgG**	Immunoglobulin G
**IHC**	Immunohistochemistry
**IL-21**	Interleukin-21; cytokine driver of B cell proliferation
**IU**	International Units
**mRNA-1273**	mRNA vaccine by Moderna
**N**	Viral nucleocapsid gene
**NHP**	Nonhuman primate
**NS**	Nasal swab; upper airway
**NTD**	N-terminal domain
**PFU**	Plaque forming unit
**qRT-PCR**	Real-time quantitative reverse transcription polymerase chain reaction
**RBD**	Receptor binding domain
**S**	Spike protein of SARS-CoV-2
**S-2P**	Spike ectodomain with 2 proline substitutions that stabilize the prefusion conformation of the spike glycoprotein
**SARS-CoV-2**	Severe acute respiratory syndrome coronavirus 2

**sgRNA**	subgenomic RNA
**sgRNA_N**	subgenomic RNA for viral nucleocapsid gene
**sgRNA_E**	subgenomic RNA for viral envelope gene
**Tfh**	T follicular helper cell; CD4+ T cell that drives B-cell proliferation
**vRNA**	Viral RNA
**VSV**	Vesicular stomatitis virus
**WA-1**	SARS-CoV-2 strain isolated in Washington USA

Tables S5. Reagents Used for Intracellular Staining.

Specificity	Clone	Fluoro-chrome	Vol. per 100∝l	Step	Manufacturer	Purpose
Dead cells		Aqua Blue	5ul of 1:40 in H2O	Pre	Invitrogen	Exclusion
CD3	SP34.2	APC-Cy7	0.156	IC	BD Biosciences	T cells
CD4	S3.5	PE-Cy5.5	1.25	Surface	Invitrogen	
CD8	RPA-T8	BV570	1.25	Surface	BioLegend	
CD45RA	5H9	PE-Cy5	0.04	Surface	BD Biosciences	Memory markers
CCR7 (CD197)	G043H7	BV650	10	Surface	BioLegend	
CXCR3 (CD183)	1C6/CXCR3	BV711	5	Surface	BD Biosciences	Tfh markers
CXCR5 (CD185)	MU5UBEE	PE	10	IC	Thermo Fisher	
PD-1 (CD279)	EH12.2H7	BV785	2.5	Surface	BD Biosciences	
ICOS (CD278)	C398.4A	PE-Cy7	0.156	Surface	BioLegend	
CD69	TP1.55.3	ECD	2.5	IC	Beckman Coulter	Background reduction
IFN-g	B27	Ax700	0.156	IC	BioLegend	Cytokines & Costimulatory ligand
IL-2	MQ1-17H12	BV750	0.625	IC	BD Biosciences	
TNF	Mab11	FITC	1.25	IC	BD Biosciences	
IL-4	MP4-25D2	BB700	5	IC	BD Biosciences	
IL-13	JES10-5A2	BV421	5	IC	BD Biosciences	
IL-17	BL168	BV605	5	IC	BioLegend	
IL-21	3A3-N2.1	Ax647	10	IC	BD Biosciences	
CD154 (CD40L)	24-31		5	IC	BioLegend	

IC = intracellular
