# Supplementary material for: Immune correlates of protection by mRNA-1273 vaccine against SARS-CoV-2 in nonhuman primates
Source: Science. 2021 Jul 29;373(6561):eabj0299. doi: 10.1126/science.abj0299 (PMC8449013; doi:10.1126/science.abj0299)
Supplement: Supplementary file 3 — MDAR Reproducibility Checklist [file science.abj0299_mdar_reproducibility_checklist.pdf]

Materials Design Analysis Reporting (MDAR) Checklist for Authors

The MDAR framework establishes a minimum set of requirements in transparent reporting applicable to studies in the life sciences (see Statement of Task: doi:10.31222/osf.io/9sm4x.). The MDAR checklist is a tool for authors, editors and others seeking to adopt the MDAR framework for transparent reporting in manuscripts and other outputs. Please refer to the MDAR Elaboration Document for additional context for the MDAR framework.

Materials

Antibodies	Yes (indicate where provided: page no/section/legend)	n/a
For commercial reagents, provide supplier name, catalogue number and RRID, if available.	Materials and Methods<br>MSD SULFO-TAGTM anti-human IgG detection antibody<br>p. 22, 4-Plex meso-scale ELISA<br><br>rabbit polyclonal SARS-CoV-2 (GeneTex, GTX135357)<br>p. 22, Histopathology and Immunohistochemistry (IHC)<br><br>Anti-CD28, anti-CD49d BD Biosciences<br>p. 26, Intracellular Cytokine Staining	

Cell materials	Yes (indicate where provided: page no/section/legend)	n/a
**Cell lines:** Provide species information, strain. Provide accession number in repository **OR** supplier name, catalog number, clone number, **OR** RRID	Materials and Methods<br>SARS-CoV-2 USA-WA1/2020 strain (BEI: NR-70038893)<br>p. 20, Rhesus Macaque Model<br><br>USA-WA1/2020 SARS-CoV-2 (BEI, NR-53780)<br>p. 21, Passive Transfer of Purified IgG Into Golden Syrian Hamsters<br><br>Wuhan-1 strain (Genbank #: MN908947.3)<br>p. 24, Lentiviral Pseudovirus Neutralization Assay<br><br>HEK293T/17 cells (ATCC CRL-11268)<br>p. 25, Lentiviral Pseudovirus Neutralization Assay<br><br>BHK21/WI-2 cells (Kerafast, EH1011)<br>p. 25, VSV Pseudovirus Neutralization Assay<br><br>VeroE6 cells (ATCC, #CRL-1586)<br>p. 25, Focus Reduction Neutralization Test<br><br>[EHC-083E (D614G SARS-CoV-2)<br>p. 25, Focus Reduction Neutralization Test	
**Primary cultures:** Provide species, strain, sex of origin, genetic modification status.		N/A

Experimental animals	Yes (indicate where provided: page no/section/legend)	n/a
**Laboratory animals:** Provide species, strain, sex, age, genetic modification status. Provide accession number in repository **OR** supplier name, catalog number, clone number, **OR** RRID	Materials and Methods<br>12 of each female and male Indian-origin rhesus macaques (ages 3-6)<br>p. 20, Rhesus macaque model<br><br>Golden Syrian hamsters, aged 6–8 weeks, were randomized into groups of eight based on weight, with each group containing a 1:1 ratio of males to females.<br>p. 21, Passive Transfer of Purified IgG Into Golden Syrian Hamsters	
**Animal observed in or captured from the field:** Provide species, sex and age where possible		N/A
**Model organisms:** Provide Accession number in repository (where relevant) **OR** RRID		N/A

Plants and microbes	Yes (indicate where provided: page no/section/legend)	n/a
**Plants:** provide species and strain, unique accession number if available, and source (including location for collected wild specimens)		N/A
**Microbes:** provide species and strain, unique accession number if available, and source		N/A

Human research participants	Yes (indicate where provided: page no/section/legend)	n/a
Identify authority granting ethics approval (IRB or equivalent committee(s), provide reference number for approval.		N/A
Provide statement confirming informed consent obtained from study participants.		N/A
Report on age and sex for all study participants.		N/A

Design

Study protocol	Yes (indicate where provided: page no/section/legend)	n/a
For clinical trials, provide the trial registration number **OR** cite DOI in manuscript.		N/A

Laboratory protocol	Yes (indicate where provided: page no/section/legend)	n/a
Provide DOI or other citation details if detailed step-by-step protocols are available.		N/A

Experimental study design (statistics details)	Yes (indicate where provided: page no/section/legend)	n/a
State whether and how the following have been done**, or** if they were not carried out.		
Sample size determination	Supplementary Materials<br>p. 4 Fig. S1, Study design: Evaluation of immune correlates of protection of mRNA-1273 in rhesus macaques<br><br>p. 12 Fig. S7, Post-challenge lung histopathological analysis and viral detection.	
Randomisation	Materials and Methods<br>Stratified randomization based on age and weight<br>p. 20, Rhesus macaque model<br><br>Stratified randomization based on weight<br>p. 21, Passive Transfer of Purified IgG Into Golden Syrian Hamsters	
Blinding		N/A
Inclusion/exclusion criteria		N/A

Sample definition and in-laboratory replication	Yes (indicate where provided: page no/section/legend)	n/a
State number of times the experiment was replicated in laboratory	Materials and Methods<br>p. 14, Antibody responses correlate with protection against SARS-CoV-2 replication	
Define whether data describe technical or biological replicates	Materials and Methods<br>Technical repeats<br>p. 14, Antibody responses correlate with protection against SARS-CoV-2 replication	

Ethics	Yes (indicate where provided: page no/section/legend)	n/a
Studies involving human participants: State details of authority granting ethics approval (IRB or equivalent committee(s), provide reference number for approval.		N/A
Studies involving experimental animals: State details of authority granting ethics approval (IRB or equivalent committee(s), provide reference number for approval.	Materials and Methods<br>Animal experiments approved by Animal Care and Use Committee of the Vaccine Research Center and Bioqual, Inc. (Rockville, MD)<br>p. 20, Rhesus macaque model	
Studies involving specimen and field samples: State if relevant permits obtained, provide details of authority approving study; if none were required, explain why.		N/A

Dual Use Research of Concern (DURC)	Yes (indicate where provided: page no/section/legend)	n/a
If study is subject to dual use research of concern, state the authority granting approval and reference number for the regulatory approval		N/A

Analysis

Attrition	Yes (indicate where provided: page no/section/legend)	n/a
State if sample or data point from the analysis is excluded, and whether the criteria for exclusion were determined and specified in advance.		N/A

Statistics	Yes (indicate where provided: page no/section/legend)	n/a
Describe statistical tests used and justify choice of tests.	Materials and Methods<br>MSD Discovery Workbench Analyses<br>p. 23, 4-Plex meso-scale ELISA<br><br>Geometric means<br>p. 24, ELISA for temporal NHP serum antibodies and hamster serum antibodies<br><br>ID50 Titers non-linear regression models<br>p. 25, Lentiviral pseudovirus neutralization assay<br><br>Antibody neutralization quantified number of foci per sample with Viridot program<br>p. 25, Focus reduction neutralization test (FRNT)<br><br>FRNT50 non-linear regression models<br>p. 26, Focus reduction neutralization test (FRNT)<br><br>Spearman's nonparametric correlation, univariate/multivariate linear analysis, likelihood ratio test, paired t-test<br>p. 26, Correlations and Statistical Analysis	

Data Availability	Yes (indicate where provided: page no/section/legend)	n/a
State whether newly created datasets are available, including protocols for access or restriction on access.		N/A
If data are publicly available, provide accession number in repository or DOI or URL.		N/A
If publicly available data are reused, provide accession number in repository or DOI or URL, where possible.		N/A

Code Availability	Yes (indicate where provided: page no/section/legend)	n/a
For all newly generated code and software essential for replicating the main findings of the study:		
State whether the code or software is available.		N/A
If code is publicly available, provide accession number in repository, or DOI or URL.		NA

Reporting

Adherence to community standards	Yes (indicate where provided: page no/section/legend)	n/a
MDAR framework recommends adoption of discipline-specific guidelines, established and endorsed through community initiatives. Journals have their own policy about requiring specific guidelines and recommendations to complement MDAR.		
State if relevant guidelines (eg., ICMJE, MIBBI, ARRIVE) have been followed, and whether a checklist (eg., CONSORT, PRISMA, ARRIVE) is provided with the manuscript.		N/A
